# Supplementary material for: Density-based longitudinal neuron tracking in high-density electrophysiological recordings
Source: Patterns (N Y). 2026 Jun 17;7(8):101590. doi: 10.1016/j.patter.2026.101590 (PMC13494646; doi:10.1016/j.patter.2026.101590)
Supplement: Supplementary file 1 [file mmc1.pdf]

Patterns, Volume *7*

Supplemental information

Density-based longitudinal neuron tracking in high-density electrophysiological recordings

Yue Huang, Hanbo Wang, Jiaming Cao, Yu Chen, Xuanning Wang, Yujie Zhao, Hengkun Ren, Qiang Zheng, and Jianing Yu

Supplemental Methods

Non-rigid motion correction

Rigid motion correction is often insufficient to capture the full extent of across-session probe displacement. In several datasets, displacement varied markedly with depth along the probe, indicating clear non-rigid motion (Figure S3A,B). For example, in Rat #3, the matched unit pairs identified by DANT exhibited a strong linear relationship between recording depth and relative displacement across sessions (Pearson's r = 0.999; Figure S3B). The estimated displacement difference between the superficial and deep regions exceeded 160 μm (Figure S3C), corresponding to 16.2% tissue expansion. Similar expansion or shrinkage patterns were also observed in other rat and mouse datasets (Figure S3G,H).

Such non-rigid motion can substantially impair neuron tracking because the waveform footprint cannot be accurately corrected with a rigid motion model. In these cases, non-rigid motion correction is therefore required. During spike sorting, a common strategy is to divide the probe into multiple spatial blocks and estimate motion separately within each block. However, this strategy is less suitable for unit-based longitudinal tracking. In contrast to spike-sorting approaches that exploit dense spike activity, cross-session tracking relies on smaller number of matched units, which are often distributed unevenly along the probe. Consequently, block-wise estimation may be unstable in sparsely sampled regions and may not accurately capture smooth depth-dependent motion.

To address these limitations, we developed a modified algorithm for non-rigid motion correction. Following the definition used in rigid motion estimation, we additionally define the depth d^i for the i-th matched unit pair as

$$d^i = \frac{y_A^i + y_B^i}{2}. \tag{1}$$

where y_A^i and y_B^i denote the depths of the matched units in sessions s_A^i and s_B^i, respectively. We then introduce two vectors, $\mathbf{k}$ and $\mathbf{b}$, each of length N_s (the number of sessions), representing depth-dependent motion (slope) and overall probe displacement (offset) across sessions. These parameters are estimated by solving

$$\mathbf{k}^*, \mathbf{b}^* = \underset{\mathbf{k},\mathbf{b}}{\arg\min} \sum_{i=1}^{N_p} [(y_A^i - y_B^i) - [(k_{s_A^i}\, d^i + b_{s_A^i}) - (k_{s_B^i}\, d^i + b_{s_B^i})]]^2. \tag{2}$$

This formulation reduces to rigid correction when all elements of $\mathbf{k}$ are zero. To ensure proper minimization, k_1 is fixed at zero, assuming no non-rigid motion in the first session. The solution

$\mathbf{b}^*$ is then re-centered by subtracting the mean of $\mathbf{k}^*\,\bar{d}+\mathbf{b}^*$, ensuring that the mean predicted motion at the average depth is zero across sessions:

$$\tilde{\mathbf{b}}^* = \mathbf{b}^* - \mathrm{mean}(\mathbf{k}^*\,\bar{d} + \mathbf{b}^*), \tag{3}$$

where

$$\bar{d} = \frac{1}{N_p}\sum_{i}^{N_p} d^i. \tag{4}$$

Unlike the rigid case, in which probe position is represented by a single value per session, the estimated probe position $p^i(d)$ at the depth d in session i is given by

$$p^i(d) = k_i^*\,d + \tilde{b}_i^*. \tag{5}$$

The subsequent waveform correction step proceeds as in the rigid case. Applied to Rat #3, this method revealed greater magnitude of motion in regions distal to the probe tip (Figure S3C). Compared with rigid motion correction, the non-rigid algorithm identified 42.2% more matched unit pairs (rigid: 23121 matches, with each neuron tracked for 8.1 ± 5.2 sessions; non-rigid: 32895 matches, with each neuron tracked for 11.1 ± 5.5 sessions, mean ± SD; Figure S3D–F). The matching probability from session 1 to session 18 increased from 0.17 to 0.38 (Figure S3E,F). Together, these results show that non-rigid motion correction substantially improves tracking performance in datasets with depth-dependent probe motion.

Supplemental Figures

Supplementary Figure 1 Probe motions across animals.

Supplementary Figure 2 Comparison of DREDge and DANT for probe motion estimation.

Supplementary Figure 3 Non-rigid motion correction.

Supplementary Figure 4 Autocuration for quality control of HDBSCAN clustering results.

Supplementary Figure 5 Behavioral paradigm, performance, and example neurons recorded across sessions in an M2 recording.

Supplementary Figure 6 Example neurons recorded across sessions in a DLS recording.

Supplementary Figure 7 Tracking performance across animals.

Supplementary Figure 8 Manual curation of DANT results: an example cluster split into two subclusters with different functional properties.

Supplementary Figure 9 Manual curation of DANT results: an example cluster with consistent functional properties.

Supplementary Figure 10 False positives and false negatives identified by manual inspection.

Supplementary Figure 11 Evaluating runtime performance.

Supplementary Figure 1

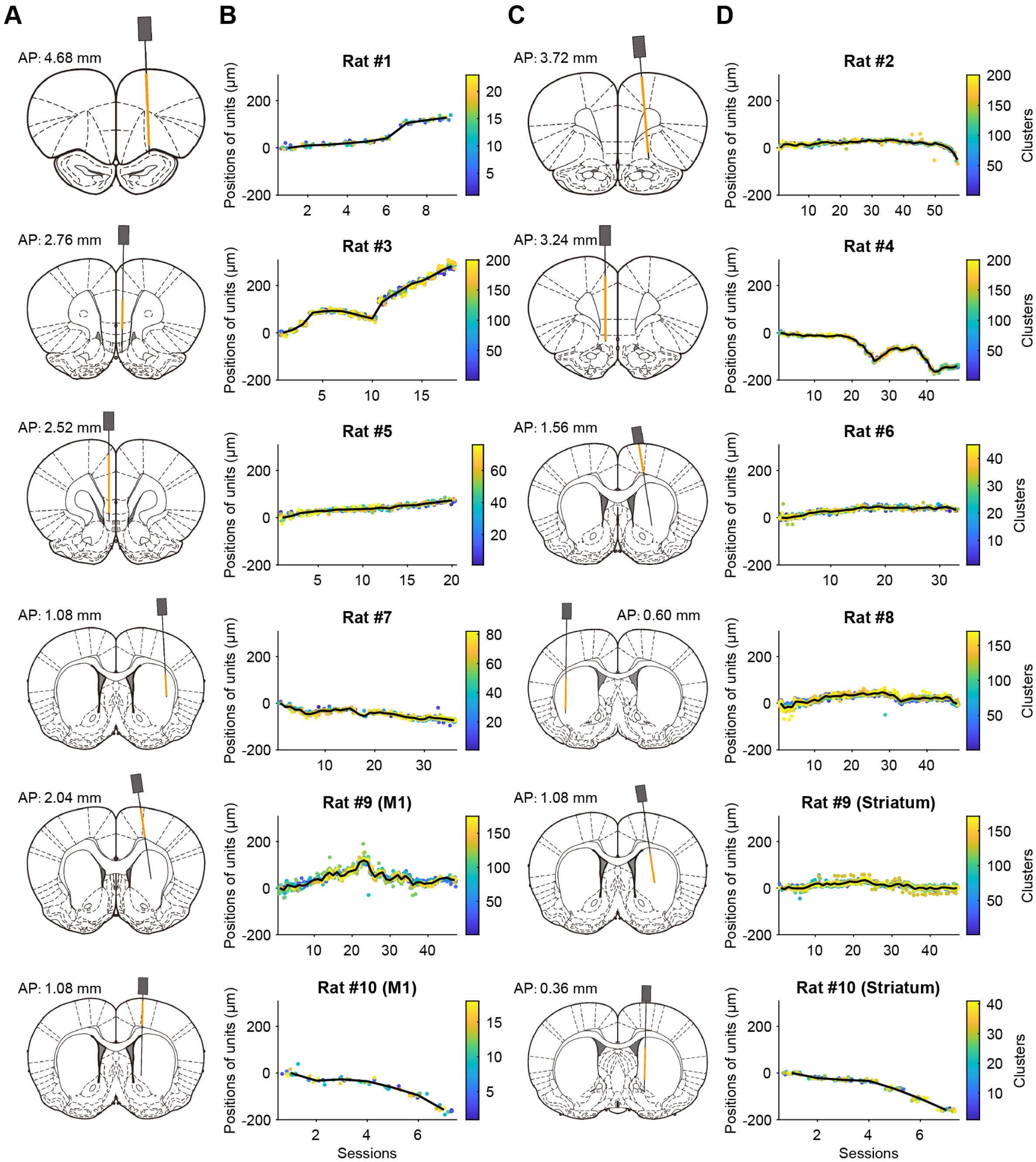

Figure S1. Probe motion across animals. (A, C) Schematics of the recording sites for each dataset. For multi-shank Neuropixels 2.0 probes, only one shank's sites are shown and the other shanks targeted similar regions. Yellow shading denotes the span of recording sites included in the DANT analysis. In two rats (#9 and #10), cortical and striatal recordings were analyzed as separate datasets since they exhibit different relative probe drifts. (B, D) Relative positions of units along the probe across sessions. The black line shows the estimated probe displacement relative to the first session. The positions of units in each detected cluster (up to 200 clusters shown) were normalized to the probe's position in the first session. Colors indicate cluster identity.

Supplementary Figure 2

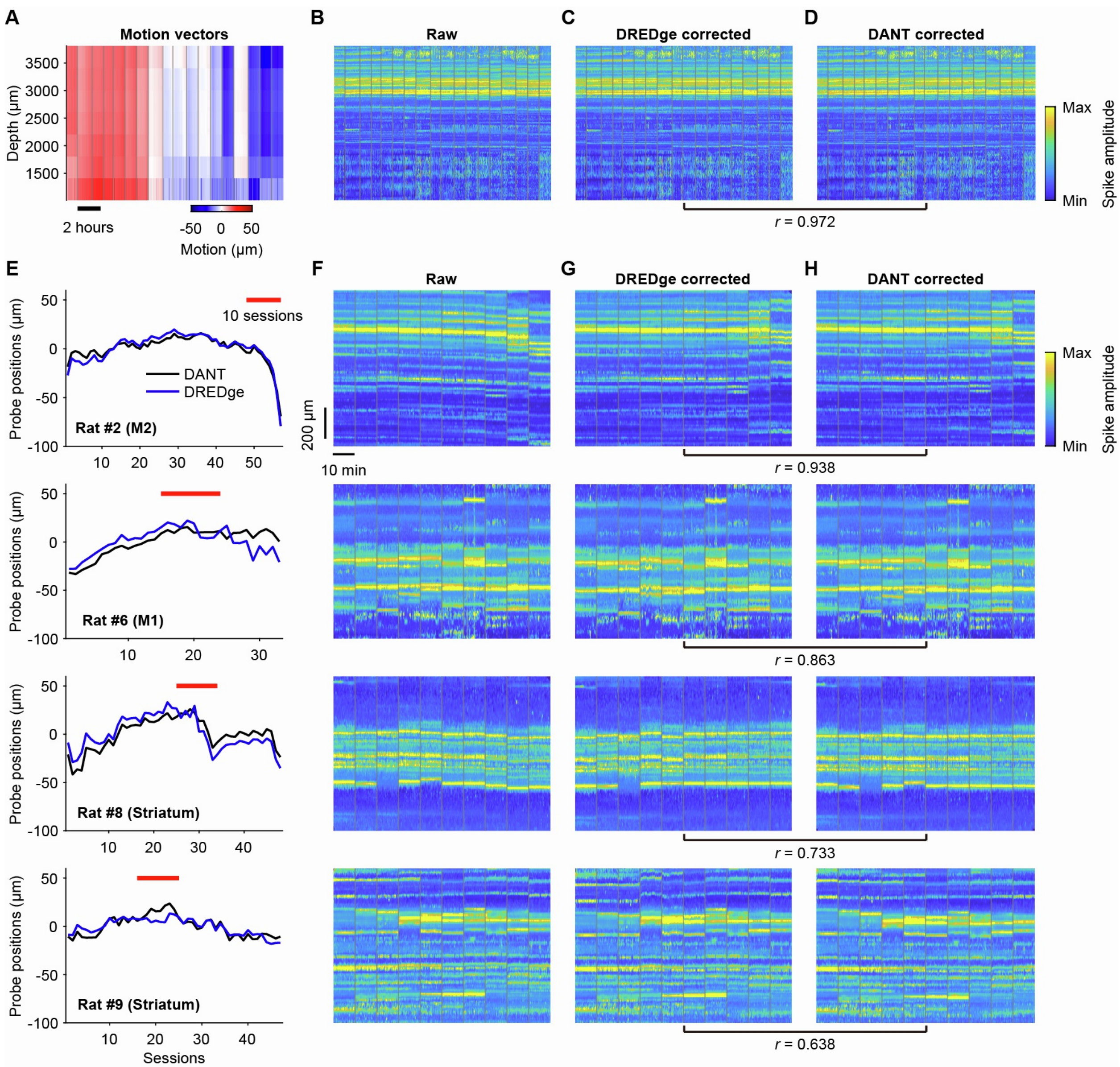

Figure S2. Comparison of DREDge and DANT for probe motion estimation. (A) Heatmap of probe motion estimated by DREDge across depth and time in an example recording spanning 18 sessions (same dataset as in Figure 2C–E), with color indicating displacement magnitude. Gray lines mark session boundaries. Probe position estimates from DREDge and DANT for this dataset are shown in Figure 2D. (B–D) Spike amplitude maps along the probe over time: before motion correction (B), after correction by DREDge (C), and after correction by DANT (D). Colors represent the mean spike amplitude within each spatiotemporal bin (min–max normalized across all sessions); gray lines mark session boundaries. Corrected spike amplitude maps are highly similar between C and D (the Pearson correlation, r, between the two maps is indicated below these panels). (E) Estimated probe positions from DANT (black) and DREDge (blue) in four additional example rats with recordings in cortex or striatum. For DREDge, median values of motion across depths within each session are shown. Red lines indicate the sessions displayed in (F–H). Probe motion is shown relative to the mean position across sessions. (F–H) Same as (B–D) for these four additional rats. Each session consists of a 10-minute recording segment used for motion correction with DREDge.

Supplementary Figure 3

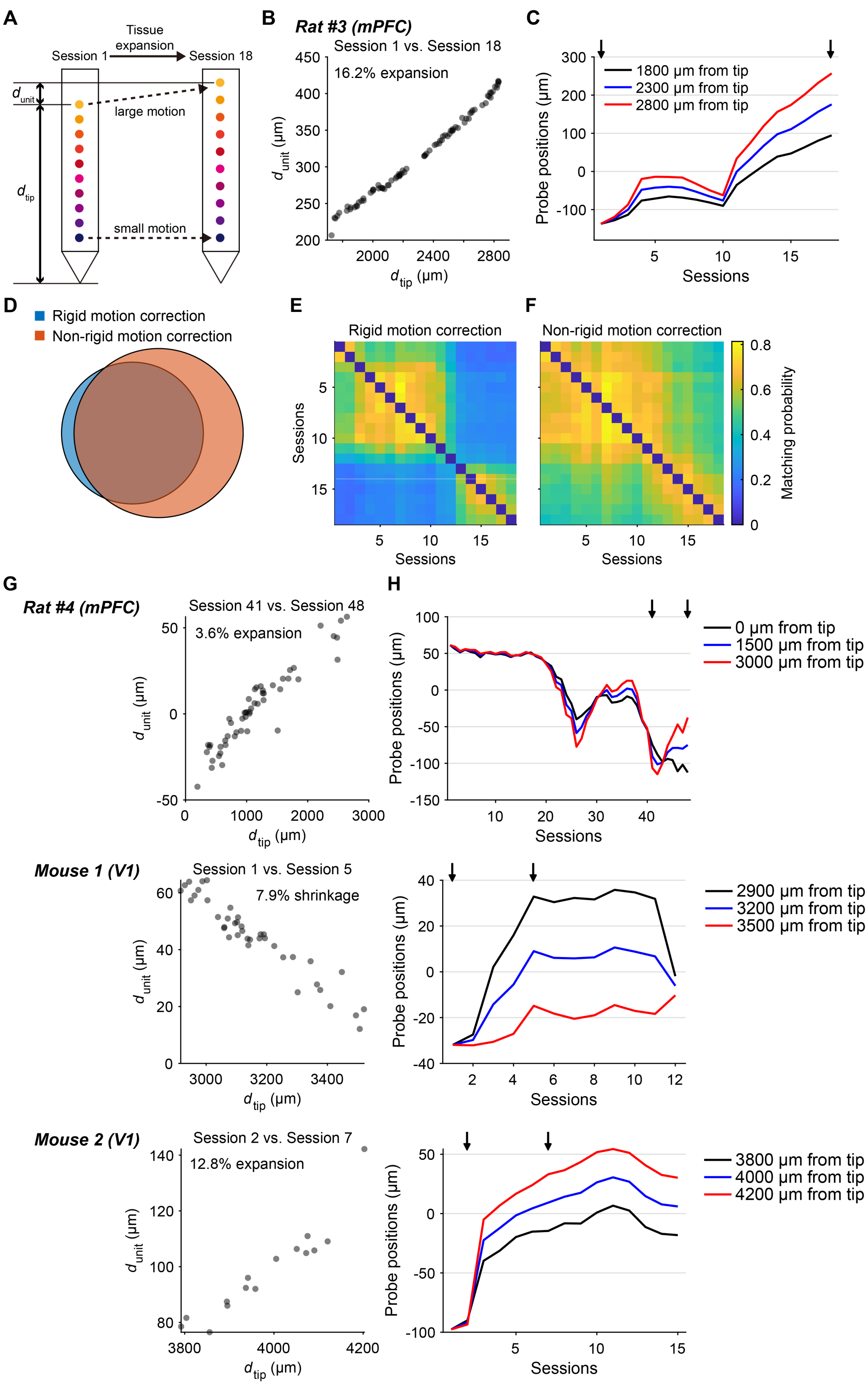

Figure S3. Non-rigid motion correction.

Figure S3 (continued). (A) Schematic illustration of depth-dependent non-rigid motion. (B) Relative motion (y-axis) between session 1 and session 18 for matched unit pairs (each dot is one pair), plotted against the average depth of each pair (x-axis) along a Neuropixels 2.0 probe shank (Rat #3). (C) Estimated depth-dependent probe motion obtained by DANT. (D) Venn diagram showing the overlap of matched units identified by DANT with rigid versus non-rigid motion correction. (E–F) Matching probability matrices showing tracking results of DANT with rigid (E) and non-rigid (F) motion correction. (G, H) Same as (B, C), but for three additional animals. mPFC, medial prefrontal cortex; V1, primary visual cortex.

Supplementary Figure 4

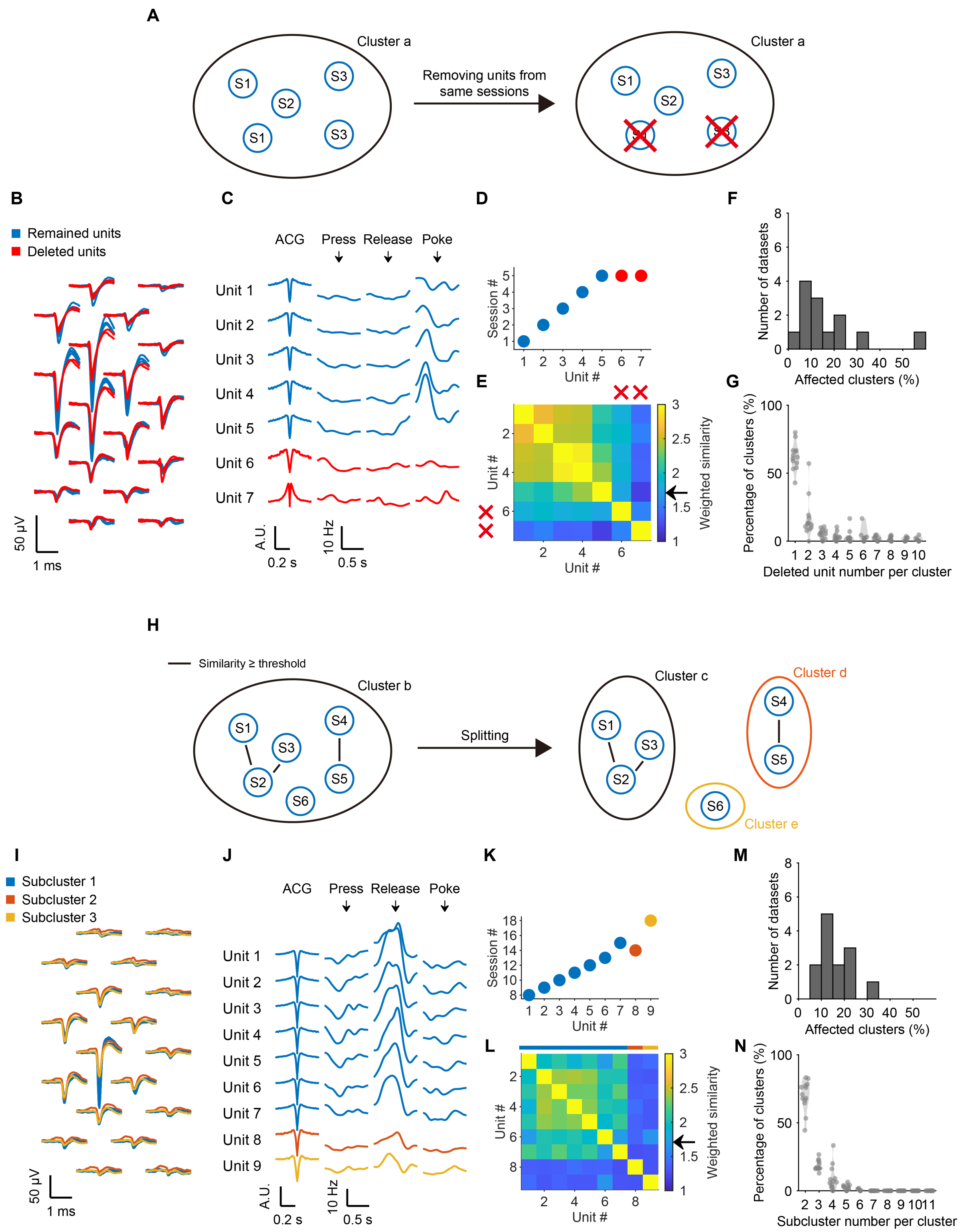

Figure S4. Autocuration for quality control of HDBSCAN clustering results.

Figure S4 (continued). (A) Schematic of Step 1, in which each cluster is constrained to one unit per recording session. When more than one unit from the same session appears, the units with the lowest average similarity to the other units are removed. (B–E) Example cluster containing multiple units from the same session. (B) Overlaid waveforms, with removed units highlighted in red. (C) Smoothed autocorrelograms and PETHs for all units in the cluster. (D) Session origins for each unit. (E) Pairwise similarity matrix; the arrow on the colorbar indicates the threshold derived from LDA. (F) Distribution across datasets of the proportion of clusters affected in Step 1. (G) Distribution across datasets of the number of units deleted per cluster in Step 1. (H) Schematic of Step 2, in which clusters are split into multiple subclusters if subsets of units exhibit higher mutual similarity than to the remaining units, based on the LDA-derived threshold. (I–L) An example cluster that is split into three subclusters in Step 2; panels follow the same layout and notation as in (B–E). (M) Distribution across datasets of the proportion of clusters affected in Step 2. (N) Distribution across datasets of the number of subclusters per original cluster in Step 2.

Supplementary Figure 5

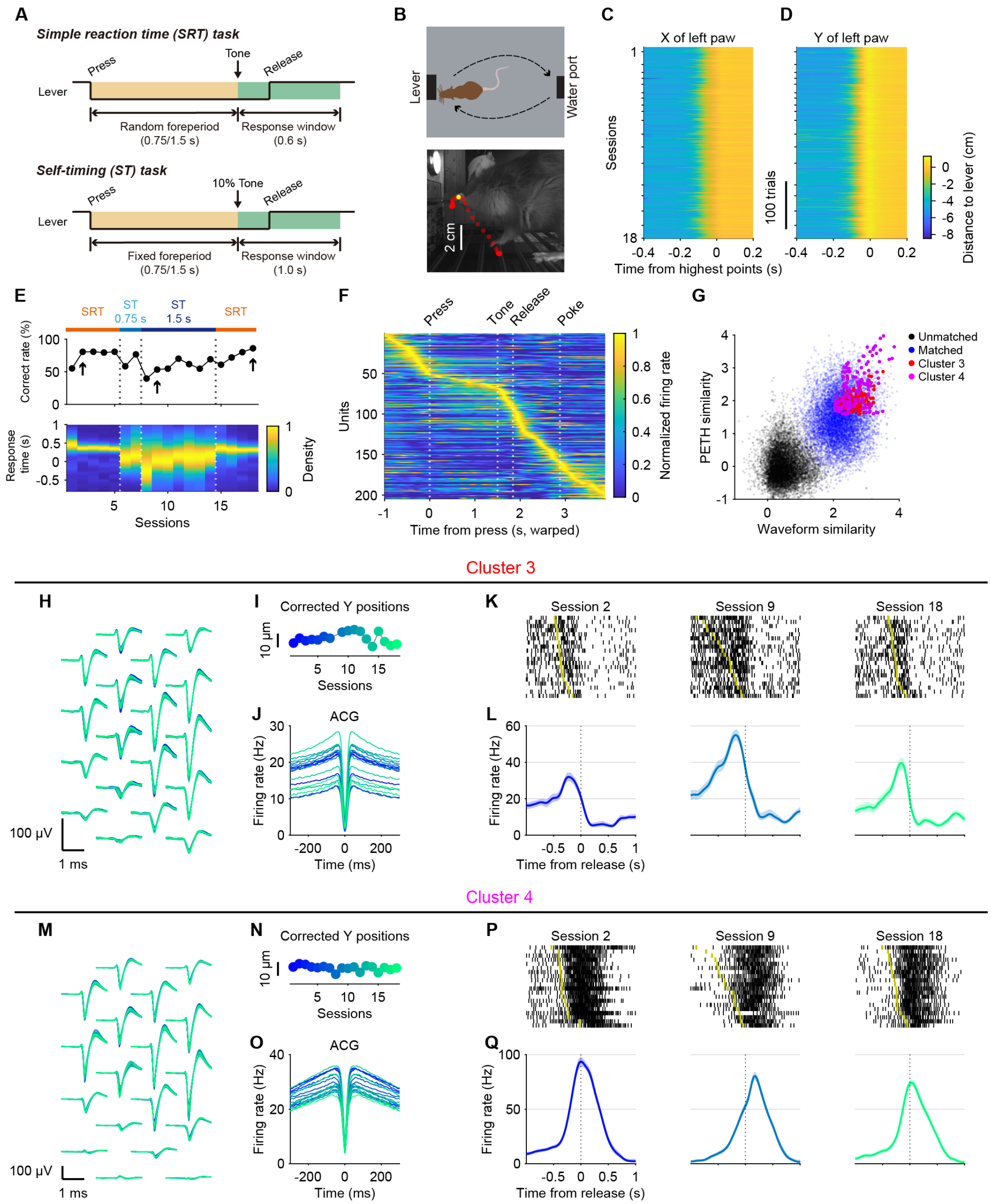

Figure S5. Behavioral paradigm, performance, and example neurons recorded across sessions in an M2 recording. (A) In the simple reaction time (SRT) task, the rat pressed and held a lever for a randomly selected foreperiod (0.75 s or 1.5 s, chosen on each trial) before releasing it within a 0.6-s response window following a brief tone (0.25 s). In the self-timing (ST) task, the rat released the lever after a fixed foreperiod (0.75 s or 1.5 s, constant within a session) within a 1-s response window, with a tone presented in only 10% of trials. (B) Top panel: schematic of the behavioral box in top view. Bottom panel: trajectory of the rat's forepaw during a lever press, with red points indicating left paw positions and a yellow point marking the peak position. The interval between successive points is 10 ms.

Figure S5 (continued). (C, D) Left paw tracking across trials and sessions, with color denoting horizontal (X) or vertical (Y) position relative to the lever. (E) Training protocol and behavioral performance across 18 sessions. Top panel shows the sequence of training phases, with the number beneath ”ST” indicating the fixed foreperiod used in the ST task. Middle panel plots the proportion of correct trials across sessions. Bottom panel displays the distribution of response times, defined as the duration from the end of the foreperiod to lever release (for premature releases, this value is negative). (F) Time-warped population PETHs for 1.5-s foreperiod trials in a single session (session 2 in the SRT task). PETHs were aligned and linearly warped between lever press, lever release, and nose poke, and were min-max normalized to 0 and 1. Units were ordered by the positions of their peak modulation. (G) PETH similarity versus waveform similarity for all pairs of units across 18 sessions, with red and purple dots highlighting unit pairs from the example neurons (153 pairwise comparisons per neuron). (H–J) Same layout as in Figure 4F–H for a different neuron (Cluster 3). Spiking activity is aligned to the time of lever release. (K) Spike raster for 1.5-s foreperiod trials aligned to the lever release. Yellow ticks indicate the end of the foreperiod. (L) PETHs aligned to the time of lever release. Shaded areas indicate the 95% confidence intervals. (M–Q) Same layout as (H–L) for a different neuron (Cluster 4).

Supplementary Figure 6

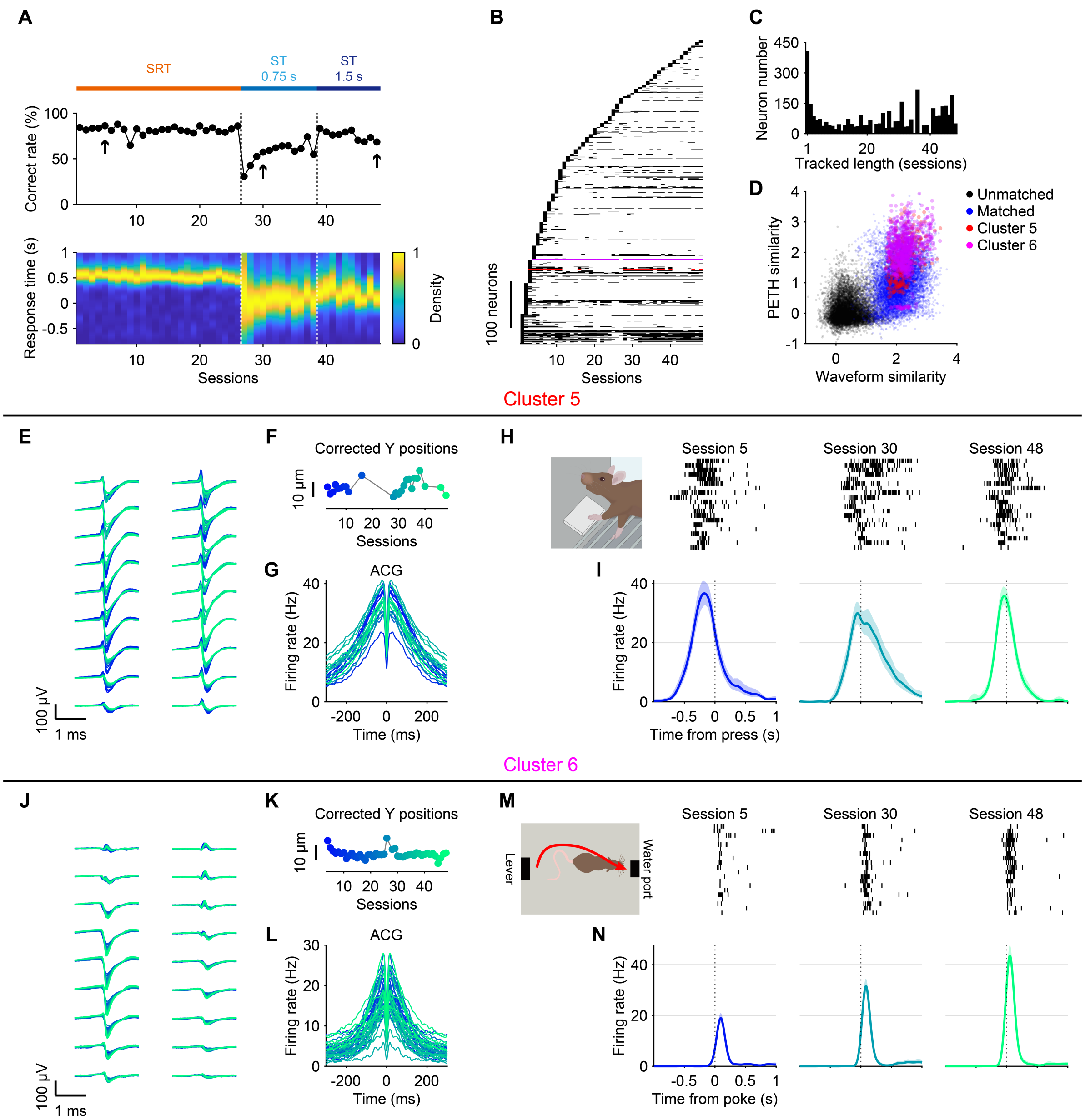

Figure S6. Example neurons recorded across sessions in a DLS recording. (A) Same layout as in Figure S5E for a different animal. (B) Presence of unique neurons across sessions, ordered by first appearance. Red and purple lines indicate the example neurons. (C) Distribution of tracked length (number of sessions in which each neuron was recorded) for all neurons. (D) PETH similarity versus waveform similarity for all pairs of units across 48 sessions, with red and purple dots highlighting unit pairs from the example neurons. (E–I) Same layout as Figure S5H–L for a different neuron (Cluster 5), with spike rasters and PETHs aligned to the time of lever press. (J–N) Same layout as (E–I) for a different neuron (Cluster 6), with spike rasters and PETHs aligned to the time of nose poke.

Supplementary Figure 7

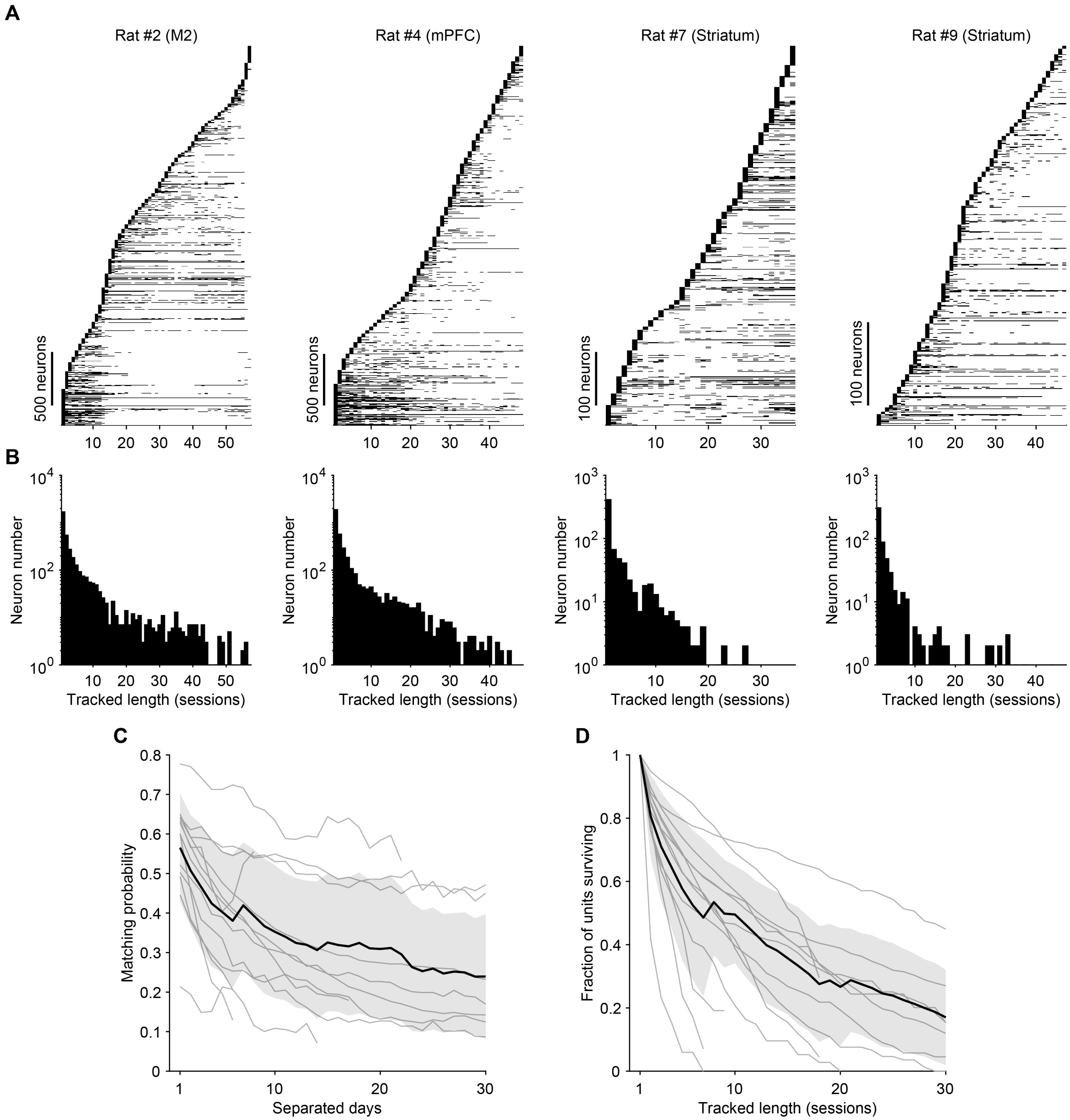

Figure S7. Tracking performance across animals. (A) Presence of unique neurons across sessions detected by DANT in four additional animals. (B) Distribution of tracked length for detected neurons. (C) Matching probability of identifying the same unit between two recordings separated by *n* days across all datasets. Black lines represent mean values, and shaded areas represent standard deviations. (D) Proportion of units that could be tracked for at least *n* sessions across all datasets. Black lines represent mean values, and shaded areas represent standard deviations.

Supplementary Figure 8

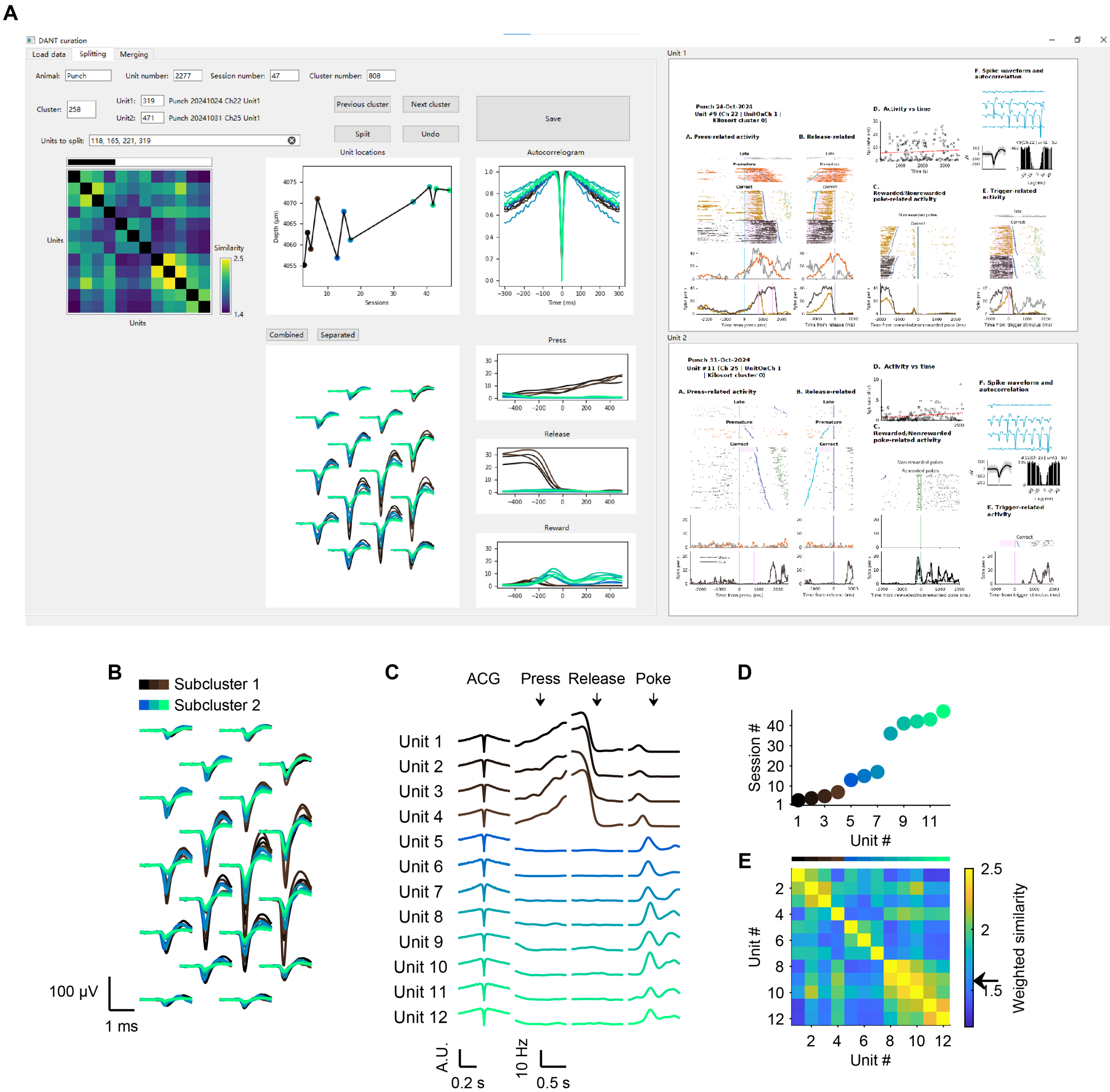

Figure S8. Manual curation of DANT results: an example cluster split into two subclusters with different functional properties. (A) Graphical user interface (GUI) used for manual curation of DANT results. The GUI allows users to visualize and curate the clusters identified by DANT. Displayed features include a similarity matrix between units, unit locations across sessions, motion-corrected waveforms, autocorrelograms, PETHs for three task events, and custom figures illustrating each unit's functional tuning. The example cluster from Rat #9 was manually divided into two subclusters. Subcluster 1 (the first four units displayed in black) exhibited ramping activity after lever press and before lever release, in clear contrast to subcluster 2 (units displayed in a blue-to-green colormap), which was primarily activated prior to nose poke for reward. The two custom figures on the right correspond to the last unit of subcluster 1 and the first unit of subcluster 2. (B–E) The example cluster from panel (A) is shown using the same layout as in Figure S4B–E.

Supplementary Figure 9

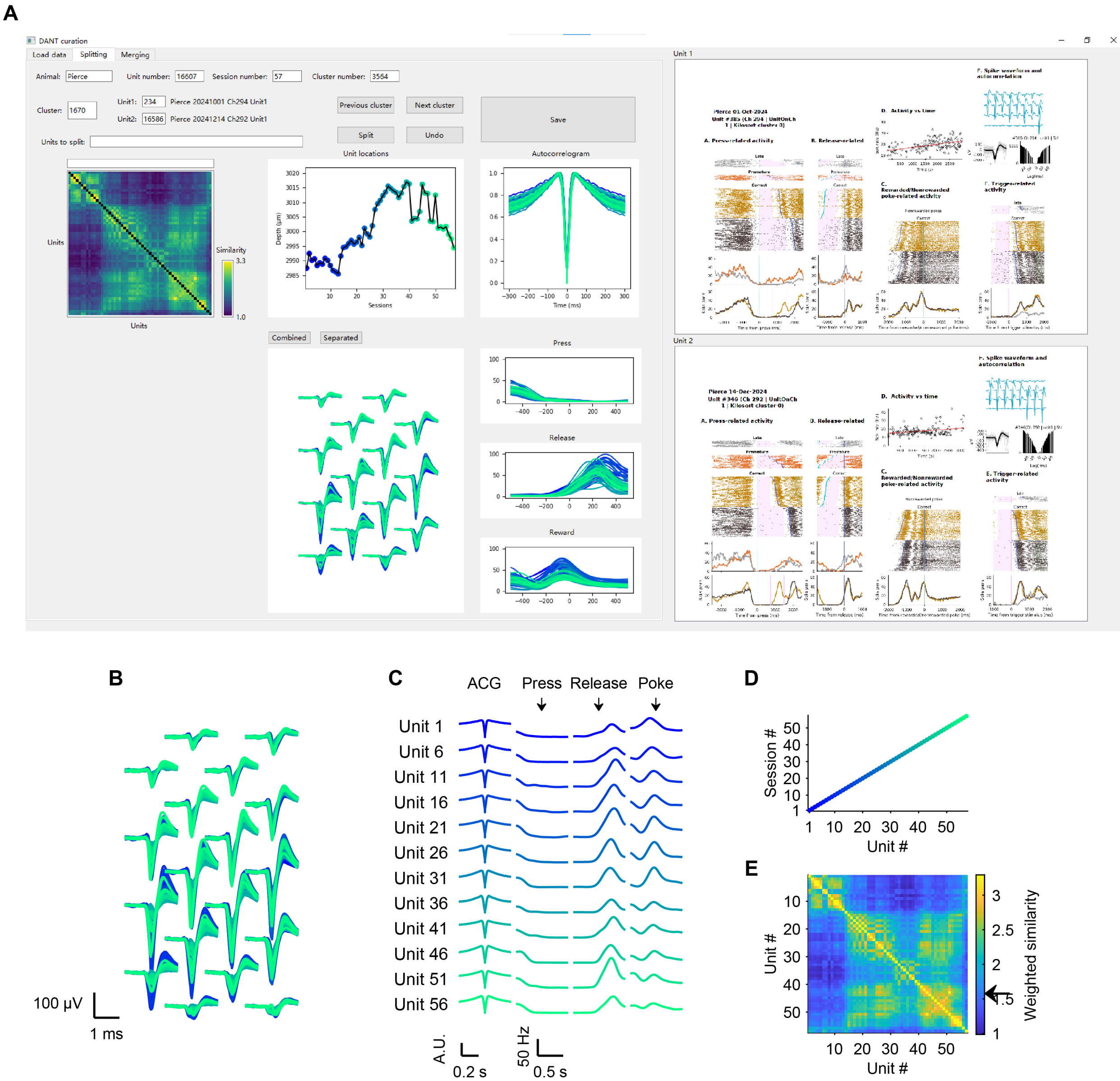

Figure S9. Manual curation of DANT results: an example cluster with consistent functional properties. Same layout as in Figure S8, but showing a cluster from Rat #2 across 57 sessions in which all units passed manual inspection, and no units were excluded.

Supplementary Figure 10

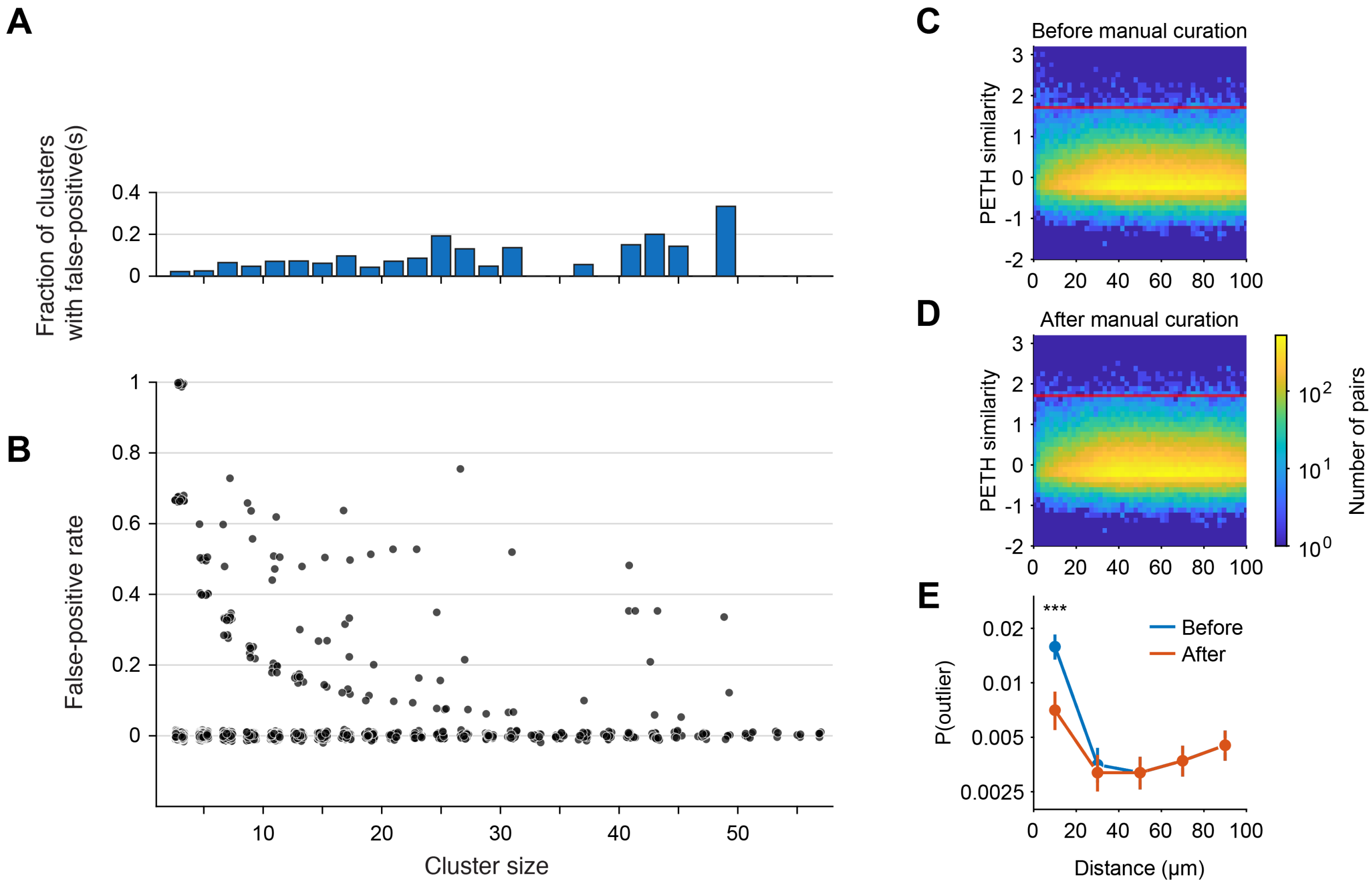

Figure S10. False positives and false negatives identified by manual inspection. (A) Fraction (in each bin) of clusters containing at least one false positive, plotted against cluster size. (B) False-positive rate for each inspected cluster, plotted against cluster size across all datasets. (C) Heatmap of PETH similarity for unmatched unit pairs identified by DANT across consecutive days, plotted as a function of inter-unit distance. Four datasets from motor cortex and dorsolateral striatum recordings were included. Pixel intensity reflects pair counts. The red line represents the threshold used to identify outliers. (D) Same as (C), but after manual curation, including merging clusters that were presumed to be the same neurons. (E) Proportion of outliers among unmatched pairs before and after manual curation, grouped into 20-μm distance bins. Error bars indicate 95% confidence intervals. ***: $p < 0.001$.

Supplementary Figure 11

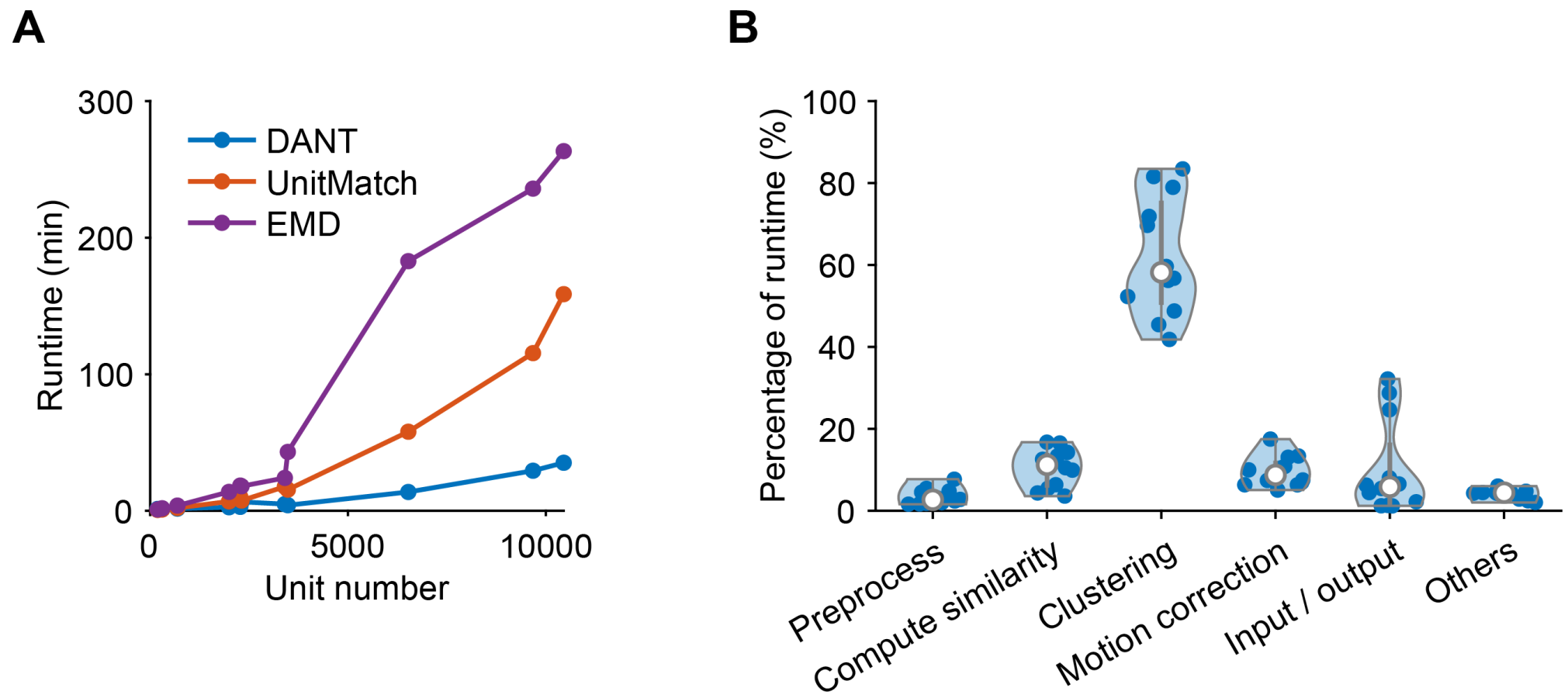

Figure S11. Evaluating runtime performance. (A) Total runtime across 11 datasets for different methods. (B) Breakdown of runtime proportions for individual processes within DANT.
