# Supplementary material for: Density-based longitudinal neuron tracking in high-density electrophysiological recordings
Source: Patterns (N Y). 2026 Jun 17;7(8):101590. doi: 10.1016/j.patter.2026.101590 (PMC13494646; doi:10.1016/j.patter.2026.101590)
Supplement: Supplementary file 2 [file mmc2.pdf]

Article

Patterns

Density-based longitudinal neuron tracking in high-density electrophysiological recordings

Graphical abstract

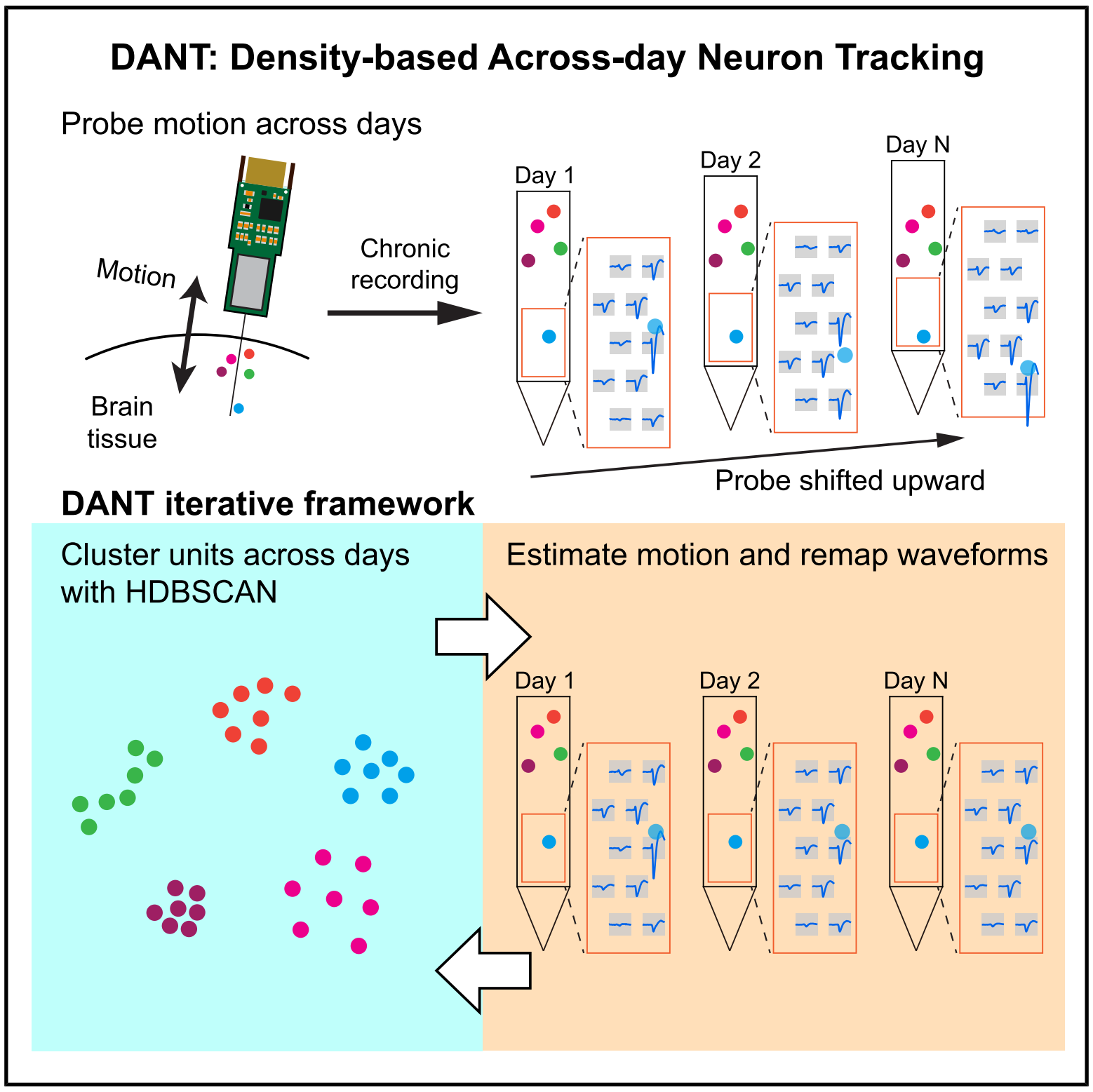

Authors

Yue Huang, Hanbo Wang, Jiaming Cao, ..., Hengkun Ren, Qiang Zheng, Jianing Yu

Correspondence

jianing.yu@gmail.com

In brief

Tracking the same neurons across days or weeks is essential for understanding learning, stability, and plasticity in the brain but remains technically challenging in chronic electrophysiology due to probe drift and unit turnover. Huang et al. present DANT, a framework using density-based clustering for longitudinal neuron tracking that improves matching yield while maintaining accuracy across Neuropixels recordings in cortex and striatum during trained motor behaviors in freely moving rats.

Highlights

- DANT tracks the same neurons across chronic recordings via density-based clustering
- Iterative loop alternates clustering with probe-motion correction
- High match yield and fast runtime
- Validated in rat motor cortex/striatum and mouse visual cortex datasets

Huang et al., 2026, Patterns 7, 101590
August 14, 2026 © 2026 The Author(s). Published by Elsevier Inc.
https://doi.org/10.1016/j.patter.2026.101590

CellPress

Patterns

CellPress
OPEN ACCESS

Article

Density-based longitudinal neuron tracking in high-density electrophysiological recordings

Yue Huang,[1,2] Hanbo Wang,[1,4] Jiaming Cao,[1,4] Yu Chen,[1,2] Xuanning Wang,[1,2] Yujie Zhao,[1,4] Hengkun Ren,[1,4] Qiang Zheng,[1,2] and Jianing Yu[1,2,3,5,*]
[1]State Key Laboratory of Membrane Biology, Peking University, Beijing 100871, China
[2]School of Life Sciences, Peking University, Beijing 100871, China
[3]IDG/McGovern Institute for Brain Research at Peking University, Beijing 100871, China
[4]Academy for Advanced Interdisciplinary Studies, Peking University, Beijing 100871, China
[5]Lead contact
*Correspondence: jianing.yu@gmail.com
https://doi.org/10.1016/j.patter.2026.101590

THE BIGGER PICTURE Understanding how the spiking activity of single neurons relates to sensory input and behavioral output has long been a central goal in neuroscience. Most electrophysiological studies characterize this relationship over relatively short time windows—typically a single session of one to several hours—during which repeated stimuli or behavioral events allow researchers to estimate what neurons represent. Several fundamental questions, however, remain less well understood. First, how stable are these neural representations over days, weeks, or months under the same conditions? Second, how are these representations established and modified during learning and experience? Third, how are they disrupted and reorganized following injury or disease, such as stroke or Parkinson's disease?

Although *in vivo* calcium imaging has been a dominant tool for studying the longitudinal activity of neurons, it cannot resolve millisecond-timescale firing patterns and faces practical limitations in deep brain regions and in freely moving animals. Moreover, the relationship between calcium fluorescence and spike trains is nonlinear, variable across cell types, and difficult to calibrate, and these features make accurate spike inference unreliable in many cases. By contrast, chronically implanted high-density probes such as Neuropixels probes—silicon electrode arrays with hundreds of closely spaced recording sites—capture neural activity with millisecond resolution, including in deep brain regions and in freely moving animals. However, identifying the same neurons across days with Neuropixels remains challenging because the waveforms captured by the probe gradually change over time, primarily due to small relative movements between the probe and brain tissue and the tissue's response to the implant. To address these challenges, clustering-based and iterative strategies can jointly infer putative matches and probe motion with minimal subjective input. By improving sensitivity and scalability, such approaches enable longitudinal studies of how individual neurons' representations evolve over extended periods.

SUMMARY

Tracking neurons across days in high-density extracellular recordings is essential for investigating the mechanisms of learning and representational drift. However, in weeks-long recordings, identifying matches across sessions is hindered by changes in spike waveforms and unit turnover. We introduce DANT (density-based across-day neuron tracking), a framework that iterates between density-based clustering in feature space and probe-motion estimation inferred from provisional matches. The estimated motion is then used to re-register spike waveforms across sessions before clustering is recomputed in the next iteration. Within this loop, DANT learns a decision boundary from match and non-match labels and uses it in post hoc curation. Applied to weeks-long Neuropixels recordings from cortex and striatum in freely moving rats during reaction-time and self-timing tasks, DANT substantially increases match yield while maintaining a low false-positive rate relative to existing approaches. These results establish DANT as a general unsupervised solution for longitudinal tracking in chronic recordings.

This is an open access article under the CC BY license (http://creativecommons.org/licenses/by/4.0/).

INTRODUCTION

High-density electrode arrays such as Neuropixels probes can simultaneously capture the activity of hundreds to thousands of neurons and, when chronically implanted, often record for months.[1–4] Because of brain motion and interactions between the implant and brain tissue,[5] some neurons are recorded across multiple days, whereas others become undetectable, and new units emerge on the probe.[3,6,7] As noted previously,[8] the persistent presence of the same neurons over days in chronic recordings presents "a problem and an opportunity." Longitudinal single-neuron tracking enables studies of representational stability and of how learning, experience, and injury shape neuronal representations.[9] In addition, within a single animal, recordings across multiple sessions with matched behavioral performance are often combined for group-level analysis, such as functional classification or encoding/decoding.[10] Reliable identification of the same neurons across sessions is therefore necessary, both to support such analyses and to avoid double counting.

Historically, same-neuron matching has been performed with data from chronically implanted electrode arrays, including microwire arrays,[11] Utah arrays,[8,12] and tetrode arrays.[13,14] Tracking typically relied on similarity in features such as spike waveforms (1–4 channels), inter-spike intervals, firing rates, stimulus- or event-related spike modulations, and cross-correlations with other neurons. Units recorded across different sessions were identified as the same neuron when their features were highly similar, with confidence established by comparing similarity distributions against null distributions (e.g., neuron pairs from different sites or animals).[8,14] Among these features, waveform similarity is often the most critical, as all other features can change with learning and plasticity. Consequently, matching becomes more reliable when waveforms from multiple closely spaced recording sites are available, as shown for tetrode recordings,[14] multisite silicon probes,[15] and polymer arrays.[16,17]

Neuropixels probes have transformed neural recordings by oversampling spikes from single neurons across many closely spaced (~20 μm) sites.[3,4] Brain movement relative to the probe is typically more pronounced along the probe's axial axis than along its lateral axis. Because Neuropixels' multisite sampling captures waveforms across the axial extent of the neuron, motion can be estimated from waveform shifts across channels and corrected by interpolation methods such as kriging.

Several methods leverage these high-density recordings to track neurons across sessions. One straightforward approach concatenates pairs of sessions and sorts them jointly.[3] This has the advantage of applying the same spike-sorting standard (e.g., the same templates in Kilosort), but its success depends on motion correction, which requires sufficiently similar activity patterns between the paired sessions. In practice, this approach also adds substantial burden to data processing, analysis, and storage, limiting scalability. Two independent alternatives have been proposed: the earth mover's distance (EMD) method, which finds an optimal matching between units across sessions by minimizing a combined cost of physical distance and waveform dissimilarity,[18] and UnitMatch, which uses a similarity score calibrated from within-session data to identify putative matches and non-matches across sessions, which are then used to train a naive Bayes classifier.[7] Both methods operate on pairwise comparisons of sessions.

Here, we introduce DANT (density-based across-day neuron tracking), a clustering approach that leverages HDBSCAN (hierarchical density-based spatial clustering of applications with noise).[19] We convert a weighted sum of similarities in features such as spike waveforms and autocorrelograms into a pairwise distance. Units recorded across sessions from the same neurons are expected to have smaller pairwise distances and to form high-density clusters in feature space, which HDBSCAN identifies. In contrast to pairwise methods, this clustering-based approach groups same-neuron units across all sessions in a single operation. Probe-motion correction and clustering are performed iteratively to enhance matching accuracy. We evaluated DANT on weeks-long Neuropixels recordings from the striatum, motor cortex, and medial prefrontal cortex of freely moving rats performing simple reaction-time (SRT) and self-timing (ST) tasks[20] and further demonstrated its generalizability on publicly available recordings from mouse visual cortex. Our results show that DANT enables longitudinal tracking of the same neurons across dozens of sessions simultaneously, substantially increasing match yield relative to existing approaches while maintaining a low false-positive rate and high computational efficiency.

RESULTS

Pipeline overview

The DANT pipeline is illustrated in Figure 1. After spike sorting each session ($1, 2, \ldots, n$) independently with Kilosort 2.5,[3,21] the well-isolated units are provided as input to DANT. Features for similarity analysis are extracted from each unit (module 1, m1; see Figure 1), including spike waveforms across all channels and, optionally, autocorrelograms (ACGs) and peri-event or peri-stimulus time histograms (PETHs or PSTHs), which capture intrinsic and stimulus- or event-driven spiking properties. Similarity scores between unit pairs are computed using feature-specific weights (default $1/3$ per feature; m2), which are then transformed into pairwise distances. A density-based clustering algorithm, HDBSCAN, is applied to these distances to identify clusters of units hypothesized to originate from the same neuron (m3). Match and non-match labels derived from these provisional clusters are used to train linear discriminant analysis (LDA), which optimizes the feature weights to maximize separation between matched and unmatched pairs (m4), yielding updated similarity scores and distances (m5). Clustering is iterated until the weights stabilize. From the spatial coordinates of matched units, relative probe motion across all sessions is inferred jointly in a single optimization (m6). Spike waveforms are then remapped to the probe's recording sites to correct for motion-induced changes in waveform distributions across channels (m7). After three iterations of m2–m7, the final clustering output (m3) is curated (m8) by removing within-cluster pairs that fall below the LDA-derived similarity threshold. Each cluster is assigned an ID (m9), representing a putative single neuron tracked across 2 to n sessions.

CellPress
OPEN ACCESS

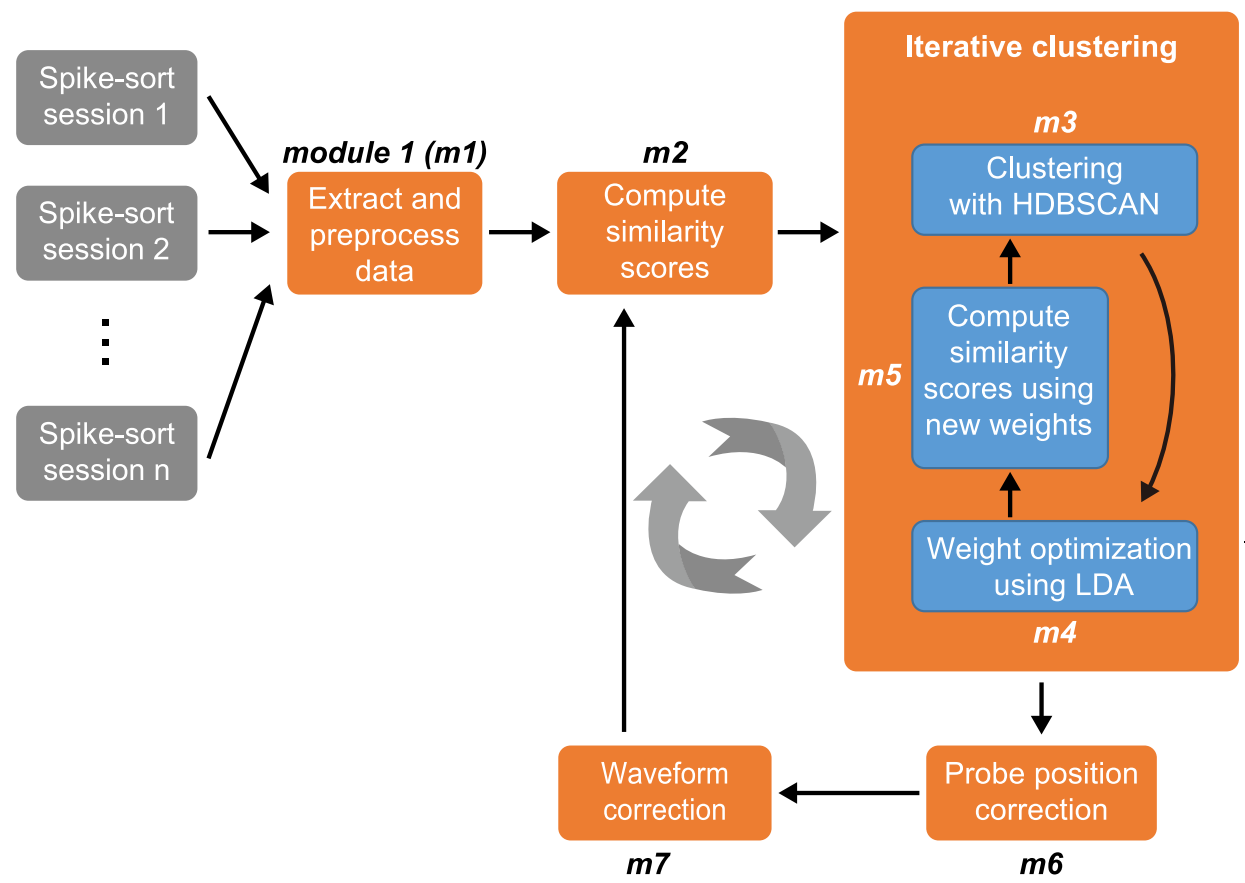

Steps	Features			Iterative clustering	Motion correction	Waveform correction	Quality control
	Waveform	ACG	PETH				
1		✓	✓	✓	✓	✓	
2	✓	✓	✓	✓	✓	✓	
3	✓	✓	✓	✓	✓	✓	
4	✓	✓		✓			✓

Figure 1. Outline of the DANT procedure

The DANT pipeline is illustrated schematically. See the main text for details.

Motion correction

In chronic preparations, despite rigid attachment of the probe to the skull, the probe and recorded neurons still undergo relative displacement due to brain motion and tissue responses to the implant.[5,22,23] These displacements reduce waveform similarity for the same neurons across sessions, complicating long-term tracking. In Neuropixels recordings, axial motion along the probe is captured by the probe's densely packed channels. In the example in Figure 2A (chronic recording in the secondary motor cortex, M2; Figure 2C), the probe shifted upward relative to the recorded neuron (i.e., toward the brain surface) between day 1 and day m, redistributing the waveforms across recording sites (Figure 2A1). Estimating this displacement (Δp_{1m}) allowed us to computationally restore the day-m probe position to its initial vertical location and interpolate spike waveforms onto the original recording sites (Figure 2A2). This correction increased waveform correlations between sessions from 0.89 to 0.97 (Figure 2B).

The three-dimensional (3D) position of recorded units relative to the Neuropixels probe was estimated using a triangulation-based method (Equation 1).[24] However, because the probe's position across sessions was unknown, we treated it as a latent variable and estimated it iteratively. First, without motion correction, we obtained a provisional set of matched units across all sessions (step 1 in Figure 1). Although this initial screening did not capture all matched units, it yielded a subset of highly similar units, forming the basis for probe-motion estimation. Across sessions, matched units from the same neurons exhibited similar but not identical patterns of relative motion (Figure 2D; see also Yuan et al.[18]). Twelve additional datasets are shown in Figure S1, yielding 13 datasets in total (including the example in Figure 2).

We then applied an optimization procedure that minimized the summed squared displacement of all matched unit pairs after correction (Equation 2 and Figure 2E), yielding probe-motion estimates. Our estimated probe motion closely resembled that derived from an alternative method, DREDge,[25] which infers displacement by aligning spike amplitude profiles along the probe (Figures S2A–S2D).

With probe motion inferred, spike waveforms were remapped (Equation 6, end of step 1), similarity scores were recomputed, and clustering was repeated (step 2), yielding a refined set of matched units that further improved the motion estimate. After one additional correction step (step 3), we performed a final clustering step (step 4). Different feature sets were selected for each step (see the table in Figure 1), with details and rationale provided below. After motion correction, the waveform similarity between matched units increased overall (Figure 2F). For the example dataset, Fisher-transformed correlation coefficients increased from 2.18 ± 0.38 to 2.41 ± 0.26 ($n = 66$ matched pairs, mean ± SD) for sessions separated by less than 7 days, and from 1.30 ± 0.34 to 2.08 ± 0.17 ($n = 87$ matched pairs, mean ± SD) for sessions separated by 7 days or more. This increase facilitated classification of matched versus unmatched pairs using spike waveforms.

Because probe motion along a Neuropixels shank can vary across depth, we incorporated non-rigid motion correction into DANT as an optional procedure (Figure S3; see supplemental methods). In this implementation, the estimated motion amplitude varies linearly across recording sites (Figure S3A). This extension improved matching performance when recordings spanned a large depth range on the probe (> 1 mm), as illustrated by two rat medial prefrontal cortex (mPFC) recordings in Figures S3B–S3H.

Feature similarity and density-based clustering

For each pair of units, we computed three feature similarity scores, expressed as Fisher z-transformed Pearson correlation coefficients: (1) waveform similarity, $\mathbf{S}_{WF}$ (Figure 3A): correlation of waveforms across channels closest to the unit (38 channels by default); (2) autocorrelogram similarity, $\mathbf{S}_{ACG}$ (Figure 3B): correlation of spike-time autocorrelograms; (3) functional similarity, $\mathbf{S}_{PETH}$ (Figure 3C): correlation of PETHs or PSTHs. These scores (or a subset of them) were then combined with weights (w_{WF}, w_{ACG}, and w_{PETH}) to form a single weighted similarity score ($\mathbf{S}$, Figure 3D). Weight optimization was carried out iteratively together with clustering (iterative clustering panel in Figures 1, 3H, and 3I).

We applied HDBSCAN to group units highly similar in selected features.[19,26,27] HDBSCAN has relatively few tunable parameters (notably the minimum cluster size and the minimum number

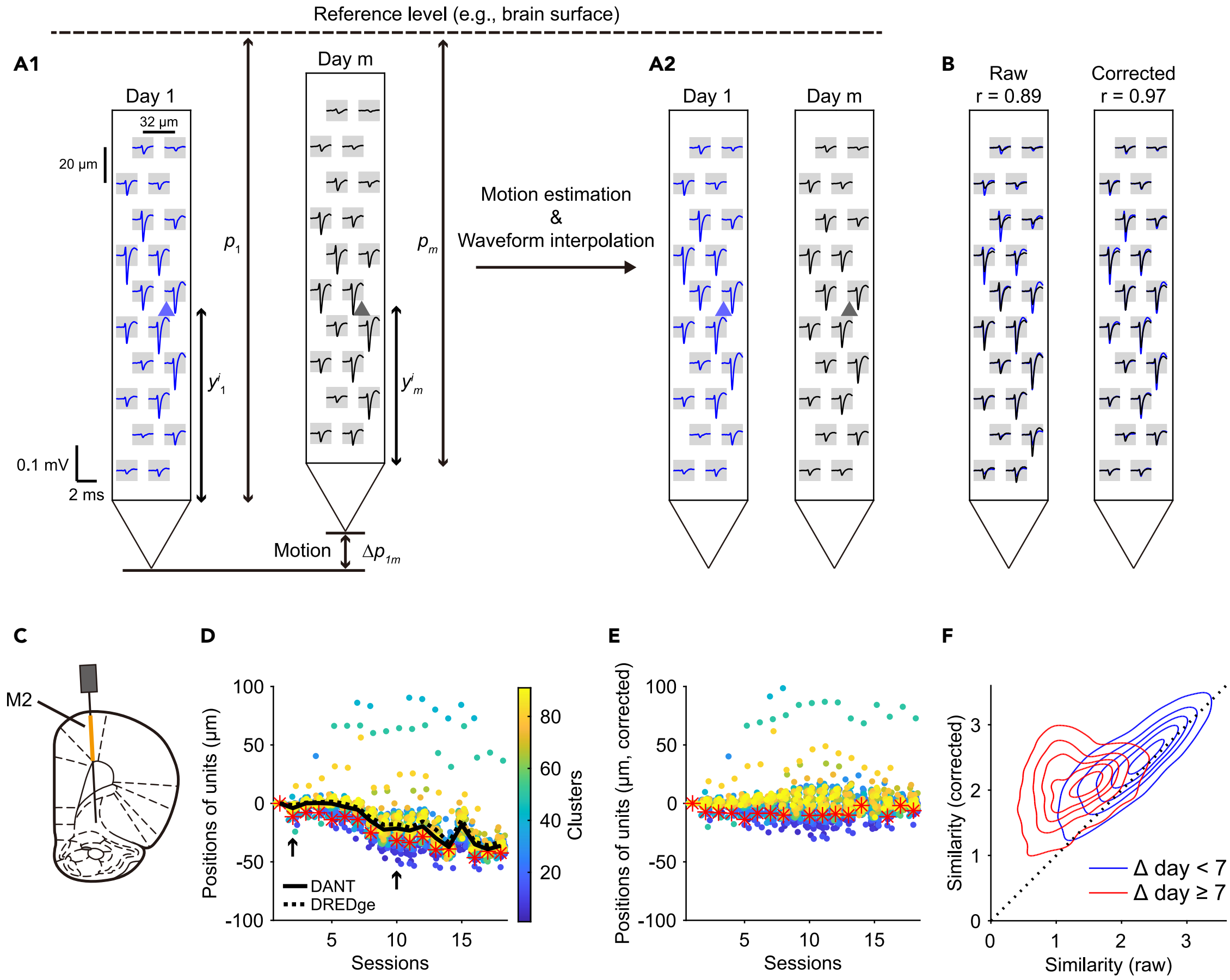

Figure 2. Probe-motion correction

(A) (1) Schematic illustrating the relative displacement between the probe and a recorded neuron (triangles) across two sessions (days 1 and *m*), resulting in a redistribution of waveforms across recording sites. (2) Illustration of waveform remapping following inference of relative probe motion.

(B) Correlation coefficients of spike waveforms with and without motion correction.

(C) Schematic of the recordings from the secondary motor cortex (M2) depicted in (A1) and (A2).

(D) Relative positions of units on the probe. Unit positions in each session are normalized to their position in the first session in which they were detected. The red asterisk marks the unit shown in (A) and (B); arrows indicate two example sessions in (A1). The thick black curve and dashed black curve denote probe position estimates by DANT and DREDge, respectively. Negative values indicate a decrease in unit positions relative to the probe tip, implying upward probe movement relative to the brain.

(E) Motion correction minimizes the summed squared displacement of relative positions of all units.

(F) Motion correction increases waveform similarity for matched unit pairs. Contours represent five density levels from 10% to 90% of the data.

of samples per core point). It makes no explicit assumptions about cluster shape while accommodating clusters with different densities. To convert similarity into a distance metric for clustering, the similarity score (**S**) was mapped to a tanh-transformed distance using $(1+\tanh(\mathbf{S}))^{-1}$ (Figure 3E). Highly similar pairs corresponded to small distances and dissimilar pairs to large distances in $[0.5, \infty)$. The resulting distance matrix served as the input to HDBSCAN, which grouped units into clusters (Figure 3F), forming groups of units likely from the same neuron across sessions (Figure 3G, dashed boxes).

The quality of clustering was visualized in two dimensions using uniform manifold approximation and projection (UMAP),[28] which approximately preserves pairwise distance relationships among units (Figure 3H). The clustering labeled each pair of units as matched or unmatched, allowing us to apply LDA to find the feature weights that maximally separate the two groups (Figure 3I). The weights, which were initially set to equal values, were updated based on the results of LDA (Figure 3I), similarity scores and pairwise distances were recomputed, and clustering was repeated.

Clustering and weight updates were iterated until weights stabilized, which often occurred after just two iterations. Contributions to the weighted similarity scores varied across features. In step 3 (Figure 1), the weights across 13 datasets were $w_{WF} = 0.51 \pm 0.12$, $w_{ACG} = 0.13 \pm 0.05$, and $w_{PETH} = 0.36 \pm 0.11$ (mean ± SD). In the final clustering step, the weights were $w_{WF} = 0.81 \pm 0.07$ and $w_{ACG} = 0.19 \pm 0.07$. Excluding $\mathbf{S}_{PETH}$ prevents functional changes across sessions from being mistaken for cross-session identity differences.

Because HDBSCAN groups points based on their relative density rather than a global threshold,[26] points that are relatively close but not truly from the same neuron can still be grouped,

CellPress
OPEN ACCESS

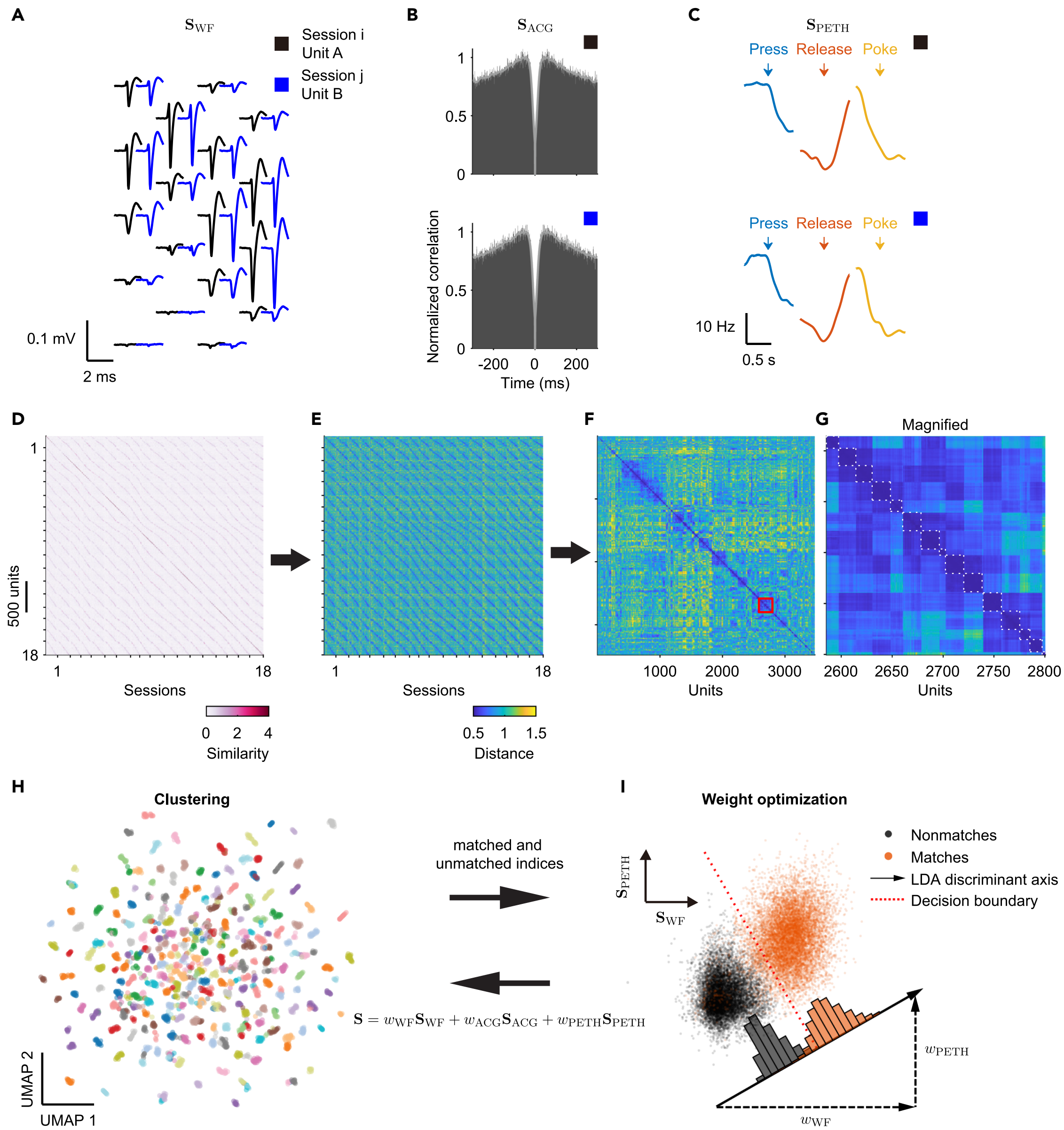

Figure 3. Density-based clustering

(A) Spike waveforms of two units from two different sessions.

(B) Smoothed autocorrelograms of these two units, scaled to $[0, 1]$.

(C) PETHs of two units, with neural activity aligned to behavioral events (lever press, lever release, and reward port nose poke) in a reaction-time task.

(D) Similarity scores for all unit pairs, computed as the weighted sum of similarity scores from selected features.

(E) Transformation of similarity scores into a distance matrix.

(F) Density-based clustering via HDBSCAN identifies clusters of units with small distances (high similarity).

(G) Zoomed-in view of the region marked by the red square in (F). Dashed squares highlight clusters identified by DANT that contain unit pairs with small pairwise distances (high similarity), indicating putative matches across sessions.

(H) UMAP embedding visualizes unit clustering using pairwise distances derived from similarity scores; each point corresponds to a unit from one session. Colors indicate distinct clusters that DANT assigns to the same putative neuron across sessions. Only 20 colors are used, so the same color may appear in more than one cluster.

(I) LDA separates matched (orange) from unmatched (black) unit pairs, optimizing feature weights to maximize discrimination. Dashed arrow lengths denote the LDA-derived weight for each feature (autocorrelogram similarity omitted for 2D visualization).

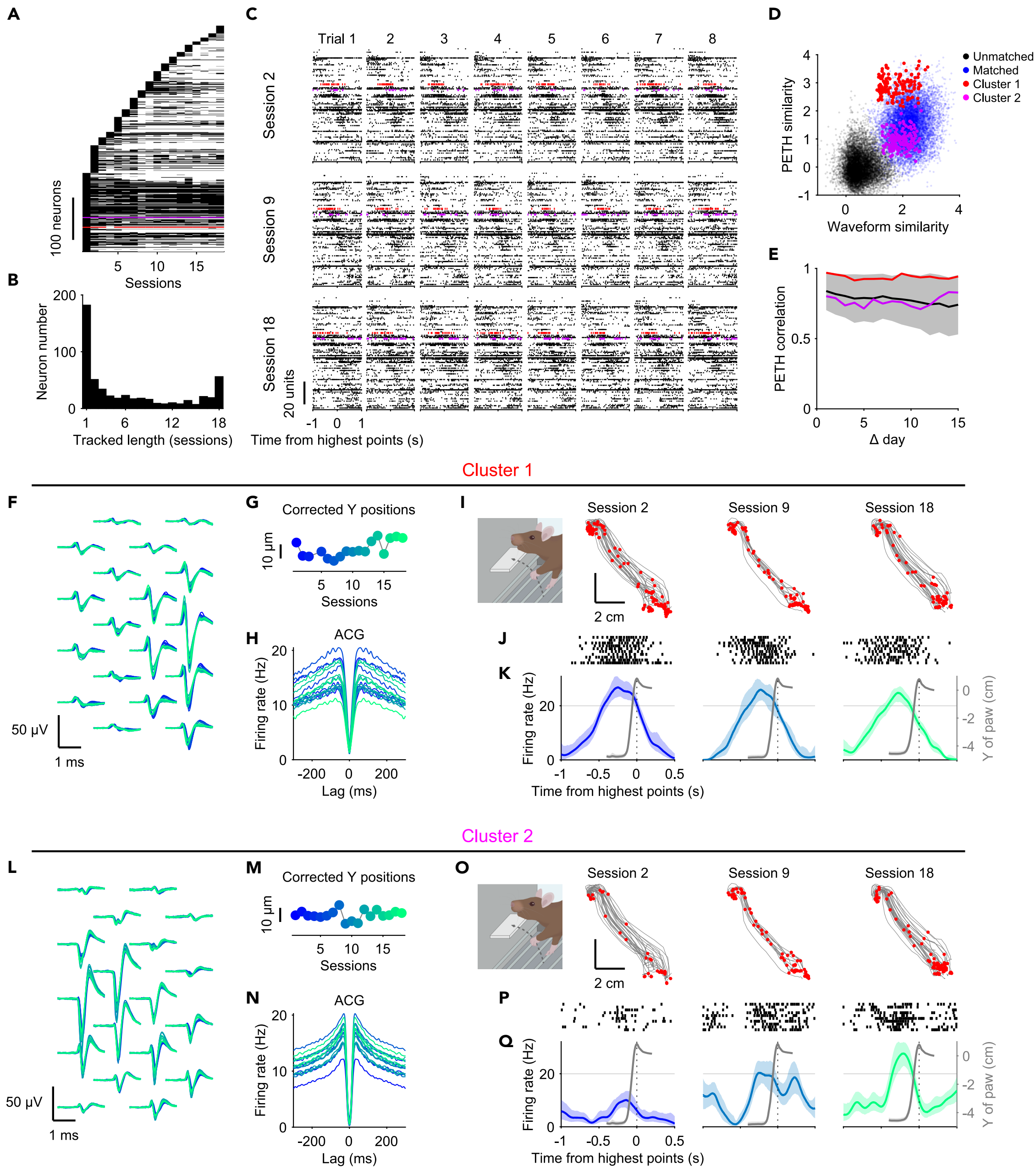

Figure 4. Example same-neuron groups in M2 recordings identified by DANT

(A) Presence of unique neurons across sessions, ordered by first appearance. Each row corresponds to a same-neuron group. Red and purple lines indicate two example neurons highlighted in subsequent panels.

(B) Distribution of tracked duration (number of sessions) for all neurons.

(C) Spike rasters of the same 99 neurons for eight consecutive example trials from each of three sessions. Only neurons tracked across these three sessions are shown, sorted by the time of their PETH peak in session 2.

(D) PETH similarity versus waveform similarity for all pairs identified by DANT, with red and purple dots highlighting unit pairs from the two example neuron groups.

(E) PETH correlation around the time of maximal paw height (lift-highest window: −1 to 0.5 s) plotted against the number of days separating the sessions. Data include neurons ($n = 60$) detected in at least ten sessions with a mean firing rate above 5 Hz during the lift window. The black line is the grand average of all 60 neurons. The shaded area indicates the standard deviation of the average correlation for each day separation. Red and purple lines represent the correlation profiles for unit pairs within the two example same-neuron groups.

(F) Overlaid waveforms after motion correction for an example neuron (cluster 1 in D).

(G) Corrected unit position relative to the probe tip.

(H) Smoothed autocorrelograms of spike times for the same neuron in individual sessions.

(legend continued on next page)

producing false-positive matches. DANT further curates HDBSCAN outputs by imposing a similarity threshold defined by the LDA discriminant boundary (m8, quality control), which we used to address two primary sources of errors. First, to avoid merging units from the same session, we enforced a constraint that each cluster contains at most one unit from any given session. Additional units from the same session exhibiting lower similarity scores were excluded (Figures S4A–S4E). Across 13 datasets, an average of 17.4% ± 14.1% of clusters (mean ± SD) contained at least one such excluded unit (2.4 ± 0.8 units per affected cluster, mean ± SD; Figures S4F and S4G). Second, in some clusters (16.3% ± 7.3%, mean ± SD; Figures S4H–S4L), units formed subgroups with substantially higher within-subgroup similarity than between-subgroup similarity. We split these clusters into subclusters using an LDA-derived boundary. On average, these affected clusters were divided into 2.4 ± 0.2 subclusters (mean ± SD; Figures S4M and S4N).

Stable and unstable functional representations in freely moving task-performing rats

We applied DANT to recordings from 11 rats performing lever-release SRT or ST tasks (Figure S5A). In the SRT paradigm, rats pressed and held a lever for a randomly selected foreperiod (0.75/1.5 s or 0.5/1/1.5 s), whereas in the ST paradigm, rats held the lever for at least the fixed foreperiod (0.75 or 1.5 s) and released it within a 1-s response window without an external cue. Although release initiation differed (tone-evoked in SRT versus self-initiated in ST), both tasks involved the same sequence of behavioral events—lever reach, press, release, and port nose poke—providing a common structure for validating cross-day neuron matching based on functional properties. We recorded in consecutive sessions using Neuropixels probes, captured task events digitally, and synchronized high-speed videography (top and side views; side view shown in Figure S5B) via an infrared light-emitting diode triggered by the stimulus or correct lever release in the ST paradigm.

For the example M2 recording (Figure 2C), the Neuropixels probe was implanted after the rat had learned the SRT task. After probe implantation, neural recordings were performed while the rat was performing the SRT task (sessions 1–5), during subsequent training on the ST task with two durations (sessions 6–14), and during retraining on the SRT task (sessions 15–18) (Figure S5E). Recordings spanned 18 sessions, during which neurons exhibited rich task-dependent modulation (Figures 4 and S5F). We tracked the rat's forelimb position during lever reaching (Figures S5C and S5D)[29] and applied DANT to examine the functional stability of neurons recorded across sessions (Figures 4A–4E). Units from the same neurons could appear and disappear in different sessions (Figure 4A). We recorded a total of 3,479 well-isolated units from 18 sessions (193.3 ± 23.3 units per session, mean ± SD) and identified 533 unique neurons. Of these neurons, 56 (10.5%) were recorded throughout all 18 sessions. The distribution of tracked length across neurons is shown in Figure 4B.

When we focused on the lever-reaching stage—from 1 s before to 0.5 s after the peak of the reach trajectory—the spike patterns exhibited overall functional stability in these neurons. In this time window, PETH correlation coefficients of matched units (60 unique neurons, 971 units in total) remained above 0.75 across more than 2 weeks of recordings. A stable neuron is shown in Figures 4F–4K (cluster 1); this neuron fired spikes before the initiation of forelimb lift, peaking just before movement onset (Figure 4K). Across different sessions (e.g., sessions 2, 9, and 18), PETHs aligned to the time of maximal reach were similar (correlation coefficient between sessions 2 and 18 = 0.950). Some units, however, showed changes in their functional properties over days. In a second example (cluster 2, Figures 4L–4Q), the coupling between spiking activity and forelimb lift was weak in session 2 but became stronger by session 18, yielding a PETH correlation of only 0.762 between those sessions.

Figures S5G–S5Q illustrate two additional neurons from the same rat that fired during lever release. Although response modulation depth exhibited modest session-to-session variability, the tracked neurons maintained consistent coupling to lever release, supporting the accuracy of our tracking method. Another recording example from the dorsolateral striatum (DLS) is shown in Figure S6 (rat #8 in Figure S1). Neural activity was recorded while the rat performed the SRT task and was later trained on the ST task with two target durations (Figure S6A). Across 48 sessions, a total of 646 unique neurons were identified from 3,404 recorded units (70.9 ± 14.6 units per session, mean ± SD; Figure S6B). Individual neurons were tracked for 24.5 ± 16.3 sessions (mean ± SD; Figures S6B and S6C). Figures S6E–S6I illustrate a neuron that increased its firing rate 0.5 s before lever press and peaked just before lever press. Figures S6J–S6N show another neuron that fired in a short time window following nose poke. These examples illustrate that DANT identifies functionally consistent matches across cortical and striatal regions in freely moving rats during behavioral tasks.

We applied DANT to 13 datasets (with four additional example datasets in Figures S7A and S7B) and evaluated the persistence of tracked neurons across time using two metrics: (1) the matching probability and (2) the tracked length. The matching probability $P_{\text{match}}(m)$ quantifies the likelihood that the same neuron can still be identified across an m-day interval. For each pair of sessions separated by m days, we computed $P_{\text{match}}(m)$ as the fraction n_{i+m}/n_i, where n_i is the number of units recorded in session i and n_{i+m} is the number of units recorded in session $i+m$ that were matched to units in session i (Figure S7C). Across datasets, the matching probability was 41.9% ± 15.9% (mean ± SD, n = 13 datasets) for m = 7 (1 week) and 30.6% ± 17.3% for m = 14 (2 weeks). The tracked length t_i of neuron i was defined as the

(I) Left paw trajectories during lever reaches for ten example trials across three sessions. The left panel shows a schematic of the rat lifting its paw to reach the lever. Red points mark spikes of the example neuron at the corresponding paw positions.
(J) Spike rasters of the example neuron.
(K) PETHs of the example neuron and vertical paw trajectories (gray), aligned to the time when the paw was at the highest point. Shaded areas indicate 95% confidence intervals of the firing rate.
(L–Q) Same as (F)–(K), but for a different neuron (cluster 2 in D).

total number of sessions—continuity not required—in which it was identified. For n recorded neurons with tracked lengths $\{t_1, t_2, \ldots, t_n\}$, we computed a survival function $S(k)$ as $\frac{1}{n}\sum_{i=1}^{n} 1_{\{t_i \geq k\}}$, describing the probability that a randomly selected neuron appears in k or more sessions. Across datasets, 48.6% ± 24.9% of neurons were tracked for at least seven sessions and 38.1% ± 18.4% for at least 14 sessions (Figure S7D).

To verify DANT's matches, we manually examined PETHs in a subset of datasets. Because neurons typically retain similar functional properties across neighboring days,[8,11,12,30–32] we visualized and inspected the PETHs corresponding to each unit in every cluster identified by DANT using a custom Python graphical user interface (GUI) (Figures S8 and S9). We examined the results of datasets from motor cortex ($n = 6$) and DLS ($n = 2$), as neurons in these regions typically show clear tuning to one or a few task events and relatively stable functional selectivity[8,12,13] (Figure S5F).

In some cases, within a cluster, units showed markedly different functional properties, indicating errors in matching. For example, in Figure S8, 12 units recorded over 12 sessions were clustered by DANT, yielding 66 within-cluster match pairs. However, visual inspection revealed that these units in fact originated from two neurons with distinct functional preferences: the first 4 units fired during lever holding, whereas the remaining 8 units fired before the nose poke. The high similarity in spike waveforms and autocorrelograms caused these units to be incorrectly clustered together. Based on this manual assessment, there were 34 true-positive matches and 32 false-positive matches. For this cluster, the false-positive rate was therefore 32/66 (48.5%). We then split the cluster into two clusters consisting of 4 and 8 units, respectively. In contrast, the majority of DANT-identified clusters contained units with consistent functional properties, representing the same neurons recorded across sessions. For example, in Figure S9, all 57 units were correctly matched and their functional properties remained similar across days despite small drifts.

Across 3,113 manually examined clusters (Figure S10), the false-positive rate was zero in 2,980 clusters (95.7%), with a mean of 1.83% ± 10.43% (mean ± SD), driven by a small number of high-error clusters. While false-positive matches still occur, post hoc curation based on functional properties or other criteria can identify and correct them. A further curation step is to examine whether separate same-neuron groups—for example, groups occupying consecutive blocks of sessions—can be merged according to well-defined criteria. This step reduces false-negative matches, which we analyze and compare with other methods in Figure 5.

Comparison with UnitMatch and EMD

We compared DANT with existing methods. Currently, three approaches exist for tracking the same neurons with Neuropixels probes across sessions. One method performs spike sorting on pairs of consecutive sessions with motion correction at their junction.[3] This approach is computationally challenging for large numbers of sessions, requiring a separate spike-sorting run for every consecutive session pair. Additionally, it requires a continuous chain of matches for longitudinal tracking of a neuron across many sessions, constrained by the joint probability of matching a neuron across all consecutive sessions. If the chain is broken, the same neuron will be assigned to different matching groups and identified as different neurons. We thus focus on comparing our method with EMD[18] and UnitMatch,[7] as these methods, like DANT, take results from spike sorting of individual sessions.

PETH features were excluded from the tracking procedures in all methods and used only to evaluate the tracking results. In an example dataset (an M2 recording), matching results derived from each method are shown in Figures 5A and 5B (the same dataset shown in Figures 2, 3, and 4). DANT identified more matches and tracked units for longer periods (20,057 matches, each neuron tracked for 12.5 ± 5.7 sessions, mean ± SD) compared with UnitMatch (11,848 matches, each neuron tracked for 7.1 ± 5.6 sessions) and EMD (6,857 matches, each neuron tracked for 4.9 ± 3.9 sessions) (Figures 5A and 5B). We observed that UnitMatch and EMD sometimes failed to track the same neurons across extended periods, instead fragmenting them into separate groups. For example, clusters identified by DANT in Figures 4F–4Q and S5H–S5Q were sometimes split into multiple groups by UnitMatch or EMD (Figure 5C).

We applied the three tracking methods to a total of 41,591 units from 12 datasets across ten rats. A linear mixed-effects model (Equation 19) was fitted to quantify neurons' tracked lengths across methods. DANT yielded the longest tracked length compared with UnitMatch and EMD (DANT 8.63 ± 1.80 sessions; UnitMatch 4.50 ± 0.98 sessions; EMD 3.63 ± 0.42 sessions; mean ± SE of estimated marginal means; Figure 5D). Post hoc pairwise comparisons of estimated marginal means (Bonferroni-corrected) confirmed that DANT's tracked lengths were significantly greater than those of both UnitMatch ($p < 0.001$) and EMD ($p = 0.002$). To assess the quality of these matches, we compared PETH similarity for matched unit pairs identified by each method on consecutive recording days. The rationale is that correctly matched units on consecutive days should show high PETH similarity. We excluded unit pairs with low firing rates and datasets with too few remaining matched pairs (4 out of 12 datasets) to ensure stable median estimates. Under these constraints, matched pairs from all three methods showed similarly high PETH similarity ($p = 0.882$, Friedman test; Figure 5E), indicating comparable matching quality.

Whereas the preceding analysis focused on matched pairs, we next examined unmatched unit pairs. In the absence of ground-truth data, examining PETH similarity between unmatched unit pairs helps compare false-negative rates across methods. In neighboring sessions, unmatched units represent either truly different neurons or the same neurons that a method failed to group, i.e., false negatives. If functional properties for a single neuron are relatively stable between two consecutive sessions, false negatives are expected to exhibit higher PETH similarity than pairs drawn from truly different neurons. A baseline distribution of PETH similarity for pairs unlikely to come from the same neuron was established by examining the PETH similarity between units that are widely separated in space along the probe.[18]

We examined the PETH similarity of unmatched unit pairs recorded across consecutive days and observed an increased proportion of pairs with high similarity at short inter-unit distances (Figure 5F), potentially reflecting false negatives. The

CellPress
OPEN ACCESS

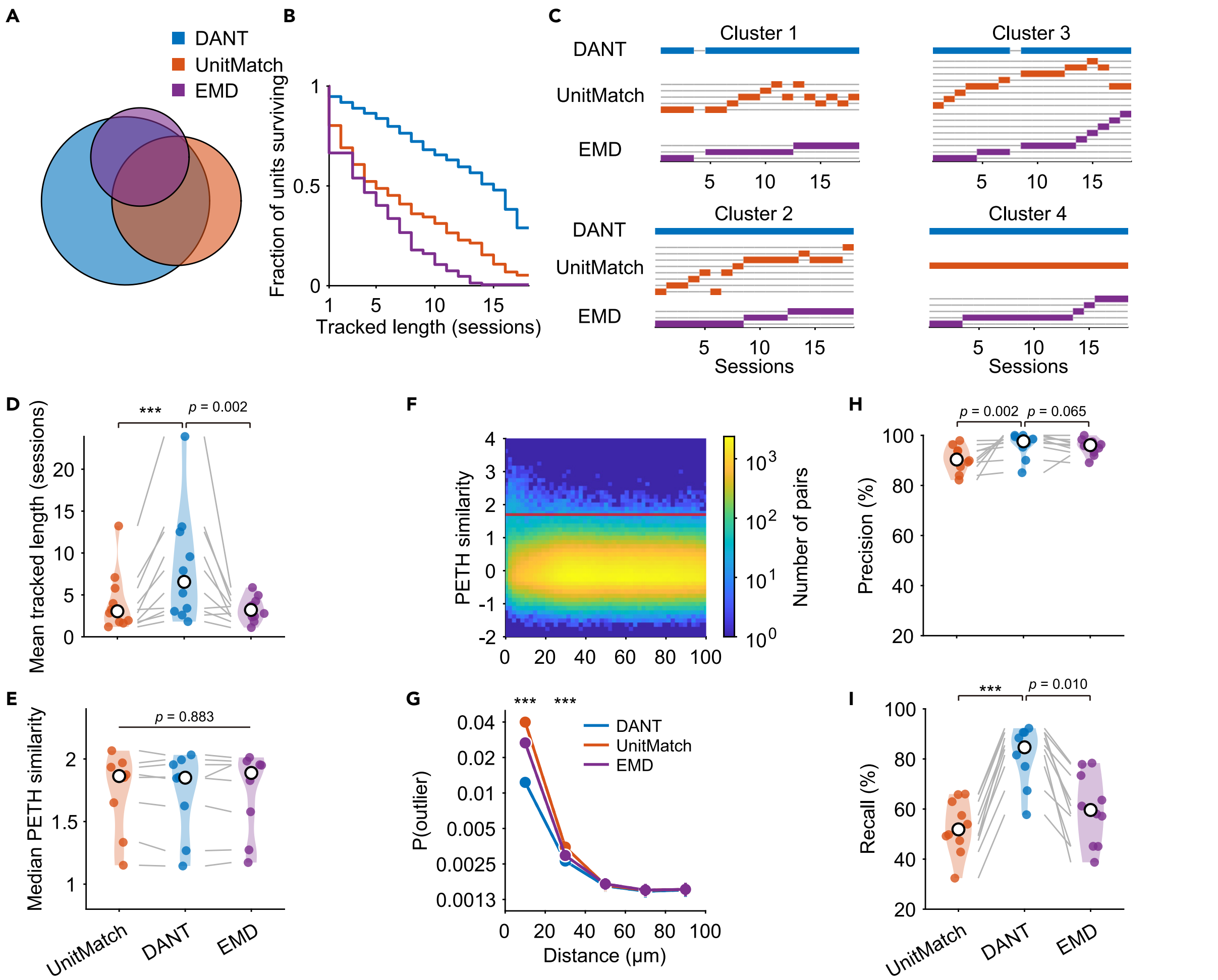

Figure 5. DANT increases matching yields compared with existing methods

(A) Venn diagram showing the overlap of matched units identified by the three different methods in an example rat.

(B) Survival function showing, for each method, the proportion of units that could be tracked for at least *n* sessions in an example rat.

(C) Tracking results for the example neurons shown in Figures 4F–4Q (clusters 1 and 2) and Figure S5 (clusters 3 and 4) obtained with the three methods. Each gray line connects units that were grouped as the same neuron by a given method.

(D) Mean tracked length for each method across 12 datasets. White points indicate median values.

(E) Median PETH similarity between matched units recorded on consecutive days across eight datasets.

(F) Heatmap of PETH similarity for unmatched unit pairs identified by DANT across consecutive days, plotted as a function of inter-unit distance. Pixel intensity reflects pair counts. The red line represents the threshold used to identify outliers.

(G) Proportion of outliers derived for each method, grouped into 20-μm distance bins. Error bars indicate 95% confidence intervals.

(H and I) Precision and recall of each evaluated method compared with Kilosort-derived matches from ten pairs of consecutive-day recordings.

***$p < 0.001$.

PETH similarity for unit pairs separated by over 50 μm was modeled with a Gaussian distribution. A threshold, $PETH_{outlier}$, was defined such that the probability of observing a value as large as or larger than $PETH_{outlier}$ under this model was less than 0.01% (red line in Figure 5F); pairs exceeding this threshold were treated as "outliers." We reasoned that a higher proportion of outliers among spatially close cross-session unit pairs that were unmatched by a method would indicate a higher false-negative rate.

The proportions of outliers were then calculated in 20-μm distance bins for each method. Significant differences in outlier rates among methods were detected in the two closest bins (0–20 μm: $\chi^2(2) = 1119$, $p < 0.001$, chi-squared test; 20–40 μm: $\chi^2(2) = 19.92$, $p < 0.001$; Figure 5G). Subsequent pairwise comparisons revealed that DANT yielded the smallest outlier rate (0.012) in the first bin compared with UnitMatch (0.04, odds ratio = 3.34, $p < 0.001$, Fisher's exact test) and EMD (0.027, odds ratio = 2.20, $p < 0.001$). Together, these results indicate that DANT exhibited the lowest false-negative rate of all three methods, consistent with the examples illustrated in Figure 5C.

The proportions of outliers in DANT's matching results can be further reduced by manual curation. As described above, using a GUI, units can be excluded from a same-neuron group when they fail to meet predefined criteria, and different same-neuron groups covering non-overlapping sessions can also be merged when there is sufficient evidence to conclude that they originate from the same

neuron, for example, when they show close proximity and similar PETHs. In four rats, manually merging separate same-neuron groups further reduced the outlier rate by more than half (from 1.58% among 9,797 unmatched unit pairs before curation to 0.71% among 9,632 unmatched unit pairs after curation) in the smallest distance bin (distance <20 μm; Figures S10C–S10E), suggesting a corresponding reduction in the false-negative rate.

To further compare these three methods against a reference close to ground truth, we selected ten consecutive session pairs from five rats (two session pairs per rat) with high unit yields (267.1 ± 121.7 units per session, mean ± SD) and jointly spike sorted each pair with Kilosort 2.5.[3] These Kilosort-derived matches were used as a reference standard. We then compared the Kilosort-based matching with EMD, UnitMatch, and DANT. Note that EMD, UnitMatch, and DANT produced tracking results using all available sessions ($n \geq 18$ per dataset), whereas the Kilosort reference was obtained from paired sessions only. Precision (for each method, the proportion of its detected matches that were also detected by Kilosort; Equation 17) and recall (the proportion of Kilosort matches that were detected by each method; Equation 18) were calculated to quantify each method's correspondence with Kilosort's results. DANT achieved the highest precision (96.0% ± 4.8%, mean ± SD; $p = 0.002$ versus UnitMatch, and $p = 0.065$ versus EMD; Figure 5H) and recall (81.5% ± 11.2%, mean ± SD; $p < 0.001$ versus UnitMatch, and $p = 0.010$ versus EMD; Figure 5I).

To compare false-positive rates across different matching methods, we took advantage of a multishank Neuropixels 2.0 probe and the fact that units recorded on different shanks cannot originate from the same neuron, using an mPFC recording from rat #3 in Figure S1 (sessions 4–10). We performed stable recordings over 7 days from two adjacent shanks (shanks 2 and 3) positioned at the same cortical depth. Each shank contained 192 channels spanning 1.425 mm. These recordings were characterized by high unit yields (162.1 ± 9.3 units on shank 2; 197.1 ± 12.8 units on shank 3; mean ± SD, $n = 7$ days) and minimal probe drift (maximum displacement of 35 μm, estimated by DANT).

We then generated a synthetic dataset by treating recordings from the two shanks as if they were recorded from the same shank on different "days" (Figure 6A). The x coordinates of corresponding channels on shanks 2 and 3 were aligned to ensure identical channel maps in the synthetic dataset. Because units from different shanks are guaranteed to arise from distinct neurons, any matches between odd and even days in this synthetic dataset are, by construction, false positives and therefore provide an estimate of the false-positive rate. Because the EMD method requires neurons to be continuously present across sessions and was therefore not applicable, we compared only DANT and UnitMatch on this synthetic dataset. Both methods identified a large number of matched unit pairs (4,926 matches, corresponding to 4.9 ± 2.2 sessions per tracked neuron on average for DANT; 3,162 matches, corresponding to 3.5 ± 2.3 sessions per tracked neuron on average for UnitMatch; Figure 6B). Critically, despite DANT finding more total matches, it maintained a lower rate of cross-shank matches (3.82% for DANT and 4.87% for UnitMatch; $\chi^2(1) = 5.02$, $p = 0.025$, chi-squared test; Figure 6C). These results demonstrate that DANT yields more total matches without a corresponding increase in the false-positive rate compared with UnitMatch.

Inspection of the cross-shank matches identified by DANT revealed highly similar waveform distributions across channels (Figures 6D–6G). Such highly similar but false-positive matches are likely to occur for same-shank matches as well, underscoring the inherent difficulty of tracking the same neuron based solely on waveforms and spike-train patterns. For a set of N unit pairs that cannot correspond to the same neuron, assume the algorithm falsely matches any given pair with probability r, yielding rN false matches on average. In our synthetic dataset, all $n_1 = rN_1$ cross-shank matches are false matches by definition. Among n_2 same-shank matches, rN_2 are expected to be false matches, resulting in a false-positive rate $\mathrm{FPR}_{n_2} = rN_2/n_2 = (N_2/N_1)(n_1/n_2)$. for same-shank matches. The numbers of possible cross-shank and same-shank pairs, N_1 and N_2, scale with the number of session pairs, assuming similar unit yields across sessions and shanks; here, $7 \times 7 = 49$ cross-shank session pairs and $2 \times \binom{7}{2} = 42$ same-shank session pairs. We can then estimate the false-positive rate in same-shank matches, FPR_{n_2}, by the following approximation:

$$\mathrm{FPR}_{n_2} \approx \frac{42}{49} \cdot \frac{n_1}{n_2} = \frac{6}{7} \cdot \frac{p}{1-p},$$

where $p = n_1/(n_1 + n_2)$ is the fraction of all matches that are cross-shank (3.8%). This yields an $\mathrm{FPR}_{n_2} \approx 3.4\%$, representing the false-positive rate expected for same-shank matches with DANT in this dataset.

This estimate, however, should not be generalized to other brain regions or recording configurations. The probability that two distinct neurons exhibit highly similar multichannel spike waveforms depends on multiple factors, including neuronal morphology relative to the probe and local cell density. In this particular analysis (Figure 6), recordings were obtained from mPFC, where the probe was oriented approximately orthogonal to the principal axis of pyramidal neuron dendritic arbors. This geometry likely limits the spread of spike waveforms along the soma-dendrite axis.

DANT was the fastest of the three methods in runtime (Figure S11), likely reflecting the efficient implementation of its clustering method, which enabled it to track over 10,000 units within 1 h.

Application of DANT to recordings from mouse visual cortex

To further evaluate the generalizability of DANT across species and recording regions, we applied it to a publicly available dataset of chronic recordings from mouse visual cortex,[3] using a pipeline closely analogous to that used for the rat recordings. For each unit, we extracted multichannel waveforms, ACGs, probe location, and a visual fingerprint defined as the mean spike-count response to each of 112 natural images. During motion estimation and correction, we incorporated the visual fingerprint alongside waveform and ACG features, as these functional responses provide additional information for cross-session alignment. As in the rat datasets, the final clustering step used only waveform and ACG features. With this procedure, DANT successfully tracked neurons across extended timescales in all three mice; examples from two mice are shown in Figure 7A. For recording sessions separated by 1 week, 56.5% ± 17.3%

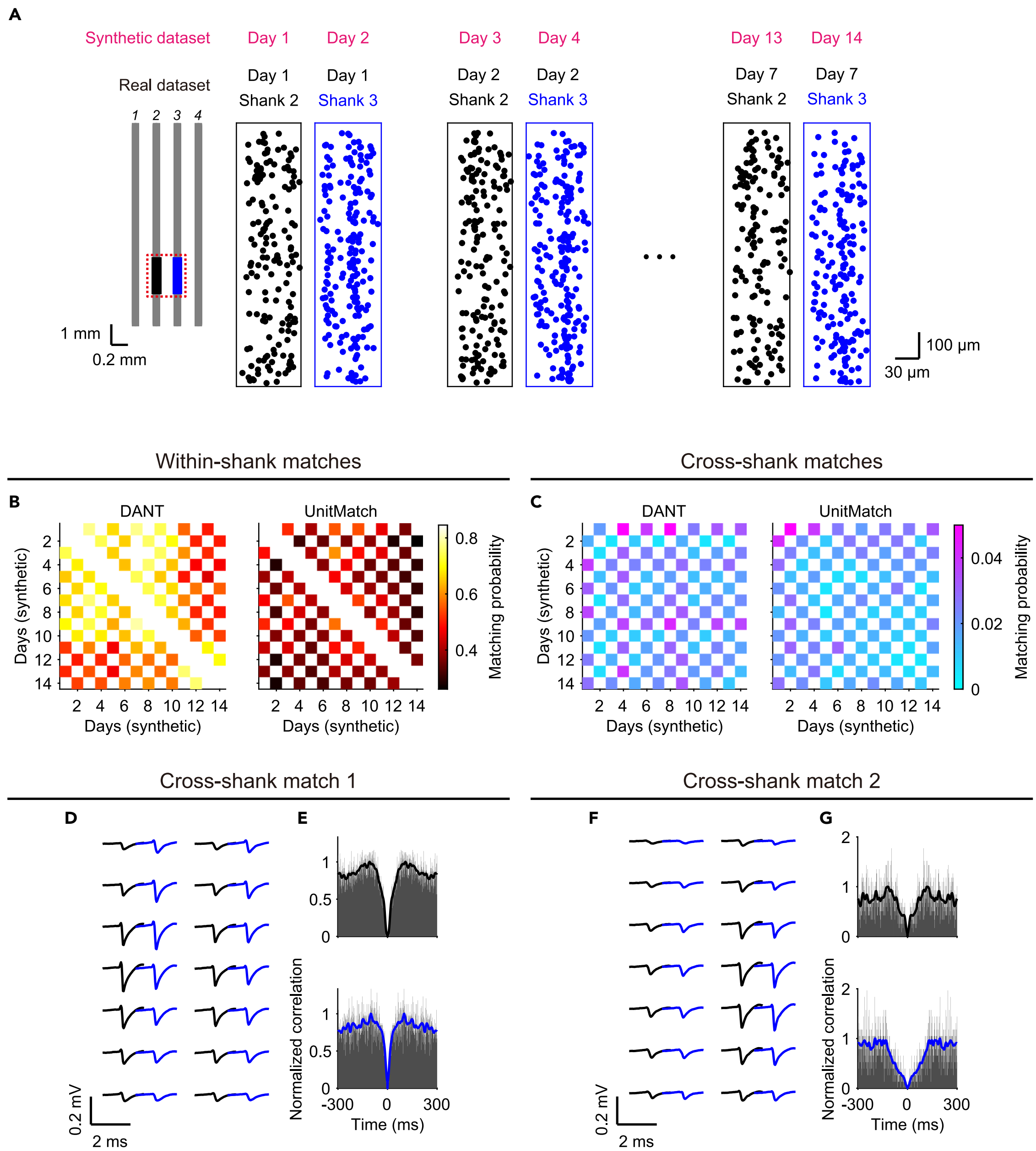

Figure 6. Performance of DANT and UnitMatch on a synthetic dataset

(A) Schematic of the synthetic dataset construction. Units recorded simultaneously from two different shanks (shank 2 and shank 3) in the same session were artificially treated as though they were recorded on separate "days." By construction, matches between units from odd and even days therefore represent false positives. Black and blue points denote the locations of recorded units along shank 2 and shank 3, respectively.

(B) Matching probability matrices showing within-shank tracking results of DANT and UnitMatch on the synthetic dataset. Pixel (i, j) represents the fraction of units from day i that were matched to day j.

(C) Same as (B) for cross-shank tracking results.

(D and E) Example of a cross-shank match identified by DANT. (D) Spike waveforms of the two units across corresponding channels. These units were recorded on shank 2 (black) and shank 3 (blue), respectively. (E) Normalized autocorrelograms of the two units.

(F and G) Same as (D) and (E) for another cross-shank match.

(mean ± SD; n = 13 session pairs from three mice) of the well-isolated units from the earlier session were successfully tracked, and 50.3% ± 14.2% (n = 49 session pairs from three mice) were tracked across sessions separated by 3–9 weeks. Example neurons exhibited stable motion-corrected waveforms, autocorrelograms, and visually evoked response profiles across widely separated recording days (Figures 7B–7E).

Following the protocol of the original study,[3] we further assessed the validity of DANT-identified matches by asking whether a unit's visual fingerprint was more similar to that of

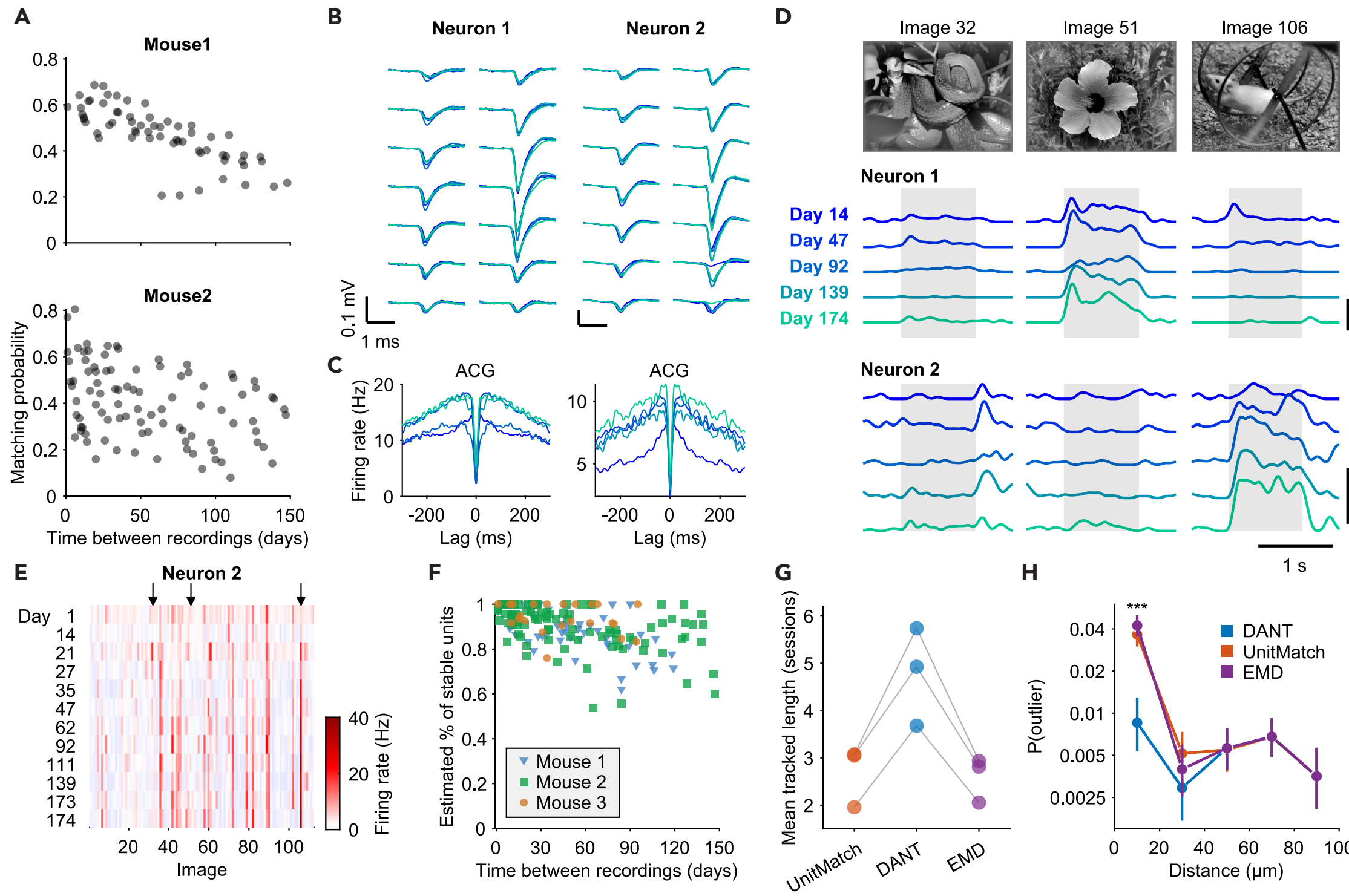

Figure 7. Tracking neurons across days in mouse primary visual cortex

(A) Matching probability for individual pairs of recording sessions plotted as a function of the interval between sessions (in days) shown for two example mice. Matching probability is defined as the number of identified matches divided by the number of units in the earlier session. Each point represents one pair of recording sessions separated by the number of days indicated on the *x* axis.

(B) Motion-corrected waveforms of two example neurons from mouse #2, overlaid across five recording days (days 14, 47, 92, 139, and 174).

(C) Smoothed autocorrelograms of the same example neurons shown in (B).

(D) Peri-stimulus time histograms (PSTHs) of the two example neurons in response to five example images presented for 1 s (gray box), averaged across $n = 5$ repetitions on each of the five recording days.

(E) Average response of a neuron (neuron 2 in D) to the full image set ($n = 112$) across days. Arrows indicate the three example images from (D).

(F) Summary of unit stability across all session pairs ($n = 175$ pairs) in the three mice. In total, 2,905 well-isolated units with visual fingerprints were analyzed. The estimated proportion of stable units was calculated as $2\,\Pr(\text{match}) - 1$, where $\Pr(\text{match})$ is the probability that a unit's visual fingerprint was more similar to that of its matched unit across two sessions than to that of its nearest neighboring unit, following the definition in Steinmetz et al.[3]

(G) Mean tracked length (in number of recording sessions) for each method across the three mouse datasets. Each point represents one mouse. Note that mouse sessions were typically separated by $\geq$ 1 week, unlike the rat datasets.

(H) Proportion of outliers derived for each method, grouped into 20-μm distance bins. Outliers were defined using visual-fingerprint similarity, following the procedure used for the rat-dataset PETH-similarity analysis in Figures 5F and 5G. Error bars indicate 95% confidence intervals.

its matched counterpart in a later session than to that of the nearest neighboring unit recorded in the same session. Matched units showed greater fingerprint similarity to their counterparts than to nearby unmatched units (Figure 7F), consistent with the results obtained using joint spike sorting of recording pairs in the original study.[3] Together, these results provide direct evidence that DANT generalizes beyond our rat motor-related datasets to mouse recordings from a sensory cortical area.

We next compared DANT with UnitMatch and EMD using procedures analogous to those in Figure 5. As in the rat datasets, functional features were excluded from tracking in all methods and used only for evaluation. Across three mouse datasets, DANT yielded the longest tracked lengths (Figure 7G). To assess false negatives, we quantified outlier rates among unmatched unit pairs as a function of inter-unit distance, following the same procedure as in Figures 5F and 5G but replacing PETH similarity with visual-fingerprint similarity and restricting the analysis to session pairs separated by 7 days or less. Because the mouse dataset was not sampled daily, this constraint resulted in a smaller pool of session pairs than for the rat datasets. Outlier rates differed significantly among methods in the closest distance bin (0–20 μm: $\chi^2(2) = 60.2$, $p < 0.001$, chi-squared test; Figure 7H). DANT showed the lowest outlier rate (0.009; 95% confidence interval [CI], 0.005–0.013), compared with UnitMatch (0.037; 95% CI, 0.030–0.044) and EMD (0.042; 95% CI, 0.035–0.050). Pairwise comparisons confirmed significant differences between DANT and both EMD and UnitMatch (both $p < 0.001$, Fisher's exact test), consistent with a lower false-negative rate for DANT. These results indicate that DANT outperformed UnitMatch and EMD in chronic mouse visual cortex recordings, yielding more matches with fewer false negatives.

DISCUSSION

Large-scale neurophysiological recordings have expanded dramatically in recent years, particularly with the introduction of Neuropixels probes.[3,4,33] Chronic Neuropixels implants now routinely yield recordings of hundreds of neurons over weeks, enabling longitudinal studies of how neural activity evolves with learning, experience, and disease. Whether neurons maintain stable functional identities across days, and—when their activity does change—whether such changes can be tied to shifts in behavior or internal state, are central open questions.[9] These questions span sensory, motor, emotional, and motivational circuits. Reliably tracking neurons longitudinally is therefore essential for studying these questions. Here, we present DANT, a fully unsupervised framework that uses density-based clustering as its central component and requires minimal subjective input. Using new datasets from chronic recordings in freely moving task-performing rats spanning brain regions including cortex and striatum, we show that DANT effectively clusters units likely to arise from the same neurons across days. Compared with existing approaches such as EMD and UnitMatch, DANT substantially increases match yield while maintaining a false-positive rate comparable to that of UnitMatch (Figure 6C). In addition, DANT reduces an outlier-based proxy for false negatives, defined as the proportion of high-similarity unmatched pairs at short inter-unit distances, by approximately 50% relative to other methods (Figure 5G), suggesting that DANT produces fewer false-negative matches. Although DANT still produces some false-positive and false-negative matches (Figures S8 and S10), our analyses indicate that, among the methods we evaluated, it provides a favorable trade-off between sensitivity and specificity, with the fastest runtime.

Unsupervised clustering has been a central tool for spike sorting, whereby spikes from the same neuron are grouped using waveform features[34] and, in some cases, their spatial location.[35] A variety of unsupervised clustering algorithms have been applied to spike sorting, including parametric methods such as Gaussian mixture models,[36] distance-based methods such as *k*-means,[37,38] and density-based methods including HDBSCAN.[34,35,39–42]

Identifying the same neurons across days in chronic recordings poses a problem similar to that for spike sorting. In some cases, spikes from multiple days are concatenated and spike sorted jointly, so that units from the same neurons are naturally identified across sessions.[3,15,43] Alternatively, when each session is spike sorted separately, units from different sessions must be compared post hoc. In previous work, similarity between two units across sessions is quantified by combining multiple feature-based scores.[8,12,14] In some work,[8,14] these scores are then modeled as arising from a mixture of "same-neuron" and "different-neuron" Gaussian distributions, with the identity (same-neuron or different-neuron) treated as a latent variable inferred by fitting a two-component Gaussian mixture using the expectation maximization (EM) algorithm. In other work, same-neuron pairs are obtained from partitions of each single unit's spike data within a session and different-neuron pairs from spatially separated electrodes.[12] A classifier is trained on these predetermined identities and then applied to classify cross-session unit pairs. Similarly, in UnitMatch, designed for Neuropixels data,[7] same- or different-neuron labels are obtained using a threshold derived from within-session data, and a naive Bayes classifier is then trained on these labels to assign match probabilities to cross-session unit pairs.

DANT takes a conceptually different approach by using HDBSCAN to group all units from all sessions simultaneously. The clustering yields match labels for all unit pairs: units within the same cluster are treated as matched and units in different clusters as unmatched. We then train an LDA classifier on these labels to learn a decision boundary that separates matched from unmatched pairs. The classifier also yields feature weights, which are used to combine multiple feature-based similarity scores into a single weighted similarity tailored to each dataset. The resulting matches are fed into a probe-motion correction step to improve waveform similarity estimation. HDBSCAN clustering, LDA training, and probe-motion correction are iterated to improve matching; the LDA decision boundary is then applied to curate the final clustering results. In this way, DANT extends unsupervised clustering from spike sorting to cross-day neuron tracking. Notably, the DANT framework is modular, with the similarity metric decoupled from the clustering procedure. This design should make it straightforward to replace the correlation-based waveform similarity used here with learned representations from a neural-network encoder, as in DeepUnitMatch.[44]

Despite its robustness, DANT still produces both false-positive and false-negative matches. First, our analysis of a synthetic dataset (Figure 6) suggests that different neurons may have very similar waveform distributions and autocorrelograms. This type of ambiguity is inherent to extracellular recordings and does not completely vanish even with dense channel layouts, contrary to earlier expectations.[45] In practice, putative same-neuron pairs can be curated post hoc based on additional criteria, such as whether the functional properties of units in a group remain reasonably stable between nearby sessions. In some systems, this assumption appears to hold,[13,43] whereas in other systems, the encoding properties of neurons are highly dynamic and can change rapidly with experience.[46,47] Thus, a general solution remains elusive: in such cases, examining the stability of functional properties is not a good criterion for identifying the same neurons. Overall, a dramatic change in neurons' functional properties is either a genuine discovery or a sign of false-positive matches. Such decisions should be based on sufficient evidence and, where possible, control experiments. Second, because HDBSCAN prioritizes cluster "stability" rather than exact pairwise similarity, units with relatively low similarity may sometimes be grouped together, whereas units with high similarity may sometimes be separated. Third, units in a same-neuron group sometimes show "disappearance-then-reappearance" patterns (e.g., cluster 5 in Figure S6). This pattern likely stems in part from fluctuations in unit quality, whereby units sorted in some sessions may fail to meet the inclusion criteria for the DANT procedure. In many cases, these "missing" units can be recovered through manual inspection with a custom GUI and, when appropriate, integrated into the same-neuron chain.

Given these remaining limitations, researchers should critically assess match quality through manual inspection of DANT's results, for example, using a custom GUI like the one shown in Figures S8 and S9. Manual inspection can help remove units that are incorrectly clustered together, for example, units within

a putative same-neuron group whose functional properties are clearly inconsistent. Conversely, groups identified by DANT as different neurons may be merged if there is sufficient evidence that they originate from the same neuron, for example, if their spike waveforms are still similar and their functional properties are consistent. Ultimately, downstream analyses should be tuned to the requirements of the specific scientific question. For example, when tracking the same neurons over time to study their dynamics, researchers may favor more conservative matching criteria to maximize confidence that all units are indeed from the same neuron; in this context, a short tracking chain of highly confident same-neuron groups is often more useful than a long chain of less confident groups. By contrast, when combining unique neurons to build a pseudopopulation, researchers may instead seek to minimize the false-negative matching rate to maximize confidence that all included units are indeed from different neurons.

A general challenge for longitudinal extracellular recording is that spike sorting is typically performed independently for each session, often using template-matching methods such as Kilosort.[21] Thus, even when the same neuron is recorded across days, the estimated waveform template and recovered spike train may vary between sessions because of differences in the templates inferred and the template-fitting solutions obtained in each session.[3,21] As a result, differences in firing patterns across sessions may partly reflect variability in how spikes are detected, assigned, split, or merged, rather than meaningful changes in the underlying neuron's activity. One possible alternative is to concatenate recordings across many sessions and perform joint spike sorting,[15,43] relying on motion-correction methods such as DREDge[25] for alignment. With sufficient alignment, it is possible for the same neuron to be represented by a common template across sessions. However, whether this can be achieved robustly over long timescales remains to be established. Even with motion correction, waveform changes across sessions may not be confined to simple shifts along the probe axis, and the same neuron may therefore still be assigned different templates in different sessions. When that occurs, additional objective inference is still required to determine whether those templates correspond to the same neuron across days. Our approach does not assume exact template agreement across days but instead seeks to make cross-session matching more robust by combining alignment across sessions with clustering based on spiking features. Nevertheless, interpretations of longitudinal stability or drift should take into account that spike detection and unit assignment remain dependent on the spike-sorting procedure. As a practical strategy, after same-neuron matching with DANT, joint spike sorting could be applied selectively to session pairs showing substantial and potentially biologically meaningful changes in firing patterns to assess whether those apparent changes reflect artifacts arising from independent spike sorting rather than true changes in the neuron's activity.

Finally, reliable longitudinal extracellular neuron tracking depends strongly on the stability of the recording preparation itself. Previous studies using multisite silicon probes rigidly fixed to the skull[31] and sealed with acrylic achieved high preparation stability (<5 μm over weeks, as reported by Schoonover et al.[15]). This stability enabled joint spike sorting of concatenated cross-day recordings without drift correction and supported longitudinal tracking through stable template identity or manual merging of separate templates.[15] In contrast, in our recordings from freely moving rats, we used a drive designed for probe recycling and covered the craniotomy with petroleum jelly (Vaseline) and bone wax.[48] Under these conditions, probe motion was on the order of 100 μm or less over weeks (Figures 2 and S1), which may be further reduced with a fixed (non-recyclable) probe configuration. In the presence of probe motion, recording on consecutive days is also advantageous: dense temporal sampling facilitates probe-motion correction and limits the accumulation of waveform changes between sessions that can hinder matching.[43] It is also important to keep experimental conditions consistent, including the animals' body weight. Dehydration and rehydration can cause brain volume to shrink or expand,[49,50] potentially leading to substantial motion of the brain relative to the probe and exacerbating inflammatory responses. These practical factors are likely to play an essential role in determining the success of long-term neuron tracking.

METHODS

Animals

All animal procedures complied with animal care standards set forth by the US National Institutes of Health and were approved by the Institutional Animal Care and Use Committee of Peking University. Male adult Brown-Norway rats (n = 11), aged at least 3 months and weighing 250–400 g prior to water restriction and behavioral training, were obtained from Vita River (Beijing, China). Rats were housed individually or in groups of 2–3 on a 12-h reverse light/dark cycle, with post-surgery housing limited to individual cages. During water restriction, water was earned through daily behavioral sessions of an SRT or ST task (6 days a week); supplemental water was provided via manual delivery at least 3 h post session if intake fell below 12 mL. Rats' body weights were monitored daily and maintained at approximately 80% of their free-feeding weight.

Surgeries

The implantation of Neuropixels probes was performed in two stages, using 3D-printed recoverable implants designed by the Buzsáki lab.[48] Rats were anesthetized with 4% isoflurane for induction and maintained at 1.5%–2% throughout the procedure, with vital signs monitored. In the first surgery, the dorsal skull surface was exposed and cleaned. Four to six skull screws were inserted to anchor the implant, and two ground screws were placed in the cranium above the cerebellum. Super-Bond C&B cement (Sun Medical, Moriyama, Shiga, Japan) was applied over the skull, except at the implantation site, to attach the 3D-printed headcap. After at least 2 weeks of recovery, the second surgery was conducted.

In the second surgery, a craniotomy was performed, followed by probe implantation targeting the motor cortex, medial prefrontal cortex, and/or the dorsal striatum. The probe was glued to a recyclable drive (R2Drive, 3Dneuro)[48] and lowered into the target areas at 1–2 μm/s using a manipulator. The craniotomy was sealed with Vaseline and bone wax to prevent leakage. Recordings began 3–7 days post implantation, after full recovery and the stabilization of the probe. Unit activity was monitored

in the home cage for 15 min daily from the second day after implantation to examine probe stability and unit yields. A custom-made cap, fitted to the headcap, secured the recording cable without a commutator. Recordings ($n = 11$ rats) were conducted daily for 1-h sessions over weeks in an SRT or ST task.

Neural data processing

Electrophysiological data were acquired using SpikeGLX software (https://billkarsh.github.io/SpikeGLX/). For recordings with Neuropixels 1.0 probes, a 300-Hz hardware high-pass filter was enabled during acquisition. The data from each session underwent preprocessing via CatGT (https://billkarsh.github.io/SpikeGLX/help/catgt_tshift/catgt_tshift/), which applied phase-shift correction and global common average referencing. Subsequently, the preprocessed data were spike sorted using a modified version of Kilosort 2.5 (https://github.com/jiumao2/Kilosort_2_5)[51] designed to bypass redundant preprocessing steps already completed by CatGT. All spike-sorting parameters were set to the original Kilosort 2.5 defaults, except that the spike-detection thresholds (ops.Th) were set to [8, 3]. For the majority of the datasets ($n = 7$ rats), the spike-sorting results were subjected to manual curation using Phy (https://github.com/cortex-lab/phy/) with custom plugins (https://github.com/jiumao2/PhyWaveformPlugin/).[52] During this stage, each putative unit was visually inspected and curated based on its mean waveform, autocorrelogram (ACG), inter-spike interval (ISI) distribution, and within-session spatial drift pattern. Units exhibiting similar waveforms and temporal features were merged if their cross-correlogram demonstrated a clear refractory period. Conversely, a unit was split into distinct subunits if it displayed a bimodal or multimodal amplitude distribution with a clear boundary. Finally, units were classified as "good" and included for subsequent analysis if they exhibited a distinct refractory period in their ACG, maintained a firing rate unconfounded by probe drift, and possessed an amplitude distribution that was not artificially truncated by the spike-detection threshold.

For the remaining large-scale datasets ($n = 4$ rats), an automated screening procedure was used instead of manual curation. Custom software (https://github.com/jiumao2/AutoCurationKilosort)[53] was utilized to identify high-quality units based on standardized quality metrics defined by Siegle et al.[54] Units were included for subsequent analysis in the DANT pipeline only if they satisfied all of the following criteria: (1) ISI violations <0.05, (2) amplitude cutoff <0.05, (3) presence ratio >0.95, and (4) nearest-neighbor hit rate >0.8. Units that failed any of these criteria were excluded from further analysis in DANT. Definitions of the ISI violations, amplitude cutoff, presence ratio, and nearest-neighbor hit-rate metrics are as follows.

- ISI violations: this metric was utilized to quantify the relative firing rate of contaminating spikes, which indicate the presence of multiple neurons within a unit. First, the total number of refractory period violations (defined as ISIs shorter than a 1.5-ms threshold) was counted. This count was then divided by the total time window during which violations could occur, yielding a raw violation rate. Finally, this rate was normalized by the overall firing rate of the unit.
- Amplitude cutoff: this metric represented the fraction of undetected spikes (an approximation of false-negative rate). A 500-bin histogram of spike amplitudes was generated and smoothed with a Gaussian kernel. The height of the histogram at the minimum recorded amplitude was extracted, and the proportion of spikes beyond the equivalent amplitude on the opposite side of the histogram peak was calculated. Relying on the assumption of a symmetrical amplitude distribution and the absence of electrode drift, this integral served as the estimated missing spike fraction.
- Presence ratio: the presence ratio was defined as the proportion of time a unit was active during a recording session. This was calculated by dividing the recording epoch into 100 equal-duration time bins and determining the fraction of bins that contained at least one spike.
- Nearest-neighbor hit rate: this metric was computed to evaluate cluster isolation in principal-component space. A k-nearest-neighbors search ($k = 5$) was performed on a subsample of up to 10,000 spikes per unit, and the hit rate was calculated as the fraction of a unit's spikes whose identified nearest neighbors were assigned to the same originating unit.

Raw waveform extraction

During the spike-sorting process, the whitening matrix was saved to a file. Because the whitening procedure redistributes waveforms across channel sites, an unwhitening step was required to extract the unwhitened raw waveforms from the preprocessed recording (the temp_wh.dat file generated by Kilosort 2.5). Specifically, by defining the whitening process as $x_{whitened} = x_{raw} W_{rot}$, the original data were accurately retrieved through the application of the inverse transformation: $x_{raw} = x_{whitened} W_{rot}^{-1}$. While this algebraic inversion provides a precise method for waveform recovery, alternative approaches exist that bypass the whitened data entirely by extracting waveforms directly from the unprocessed recordings and subsequently applying additional filtering steps.

Behavioral data processing

Rats were initially trained in an SRT task,[20] requiring them to press and hold a lever during a randomized foreperiod (0.75/1.5 s or 0.5/1/1.5 s) before releasing it in response to an auditory tone. Correct trials were rewarded with water accessed via port poking. Animals achieved expert-level performance prior to electrode implantation. After surgery, rats performed either the original task or an ST task variant where the foreperiod duration (0.75 or 1.5 s) remained fixed, requiring internal estimation of the interval and lever release within a 1-s response window. Lever-press, lever-release, and port-nose-poke time stamps from correct trials were used to compute peri-event time histograms (PETHs; ±500 ms, 1-ms bins), smoothed with a Gaussian kernel (standard deviation: 50 ms). The position of the left paw when the rat reached for the lever was tracked with DeepLabCut.[29] About 300 frames were manually labeled, and the DeepLabCut training was done with default parameters in the TensorFlow environment. For each video clip centered on lever press, we manually inspected all trials to verify tracking quality and excluded trials with occlusions or atypical movements (e.g., pressing the lever with the head).

For each unit, we compiled (1) mean waveforms across all recorded channels; (2) spike times; (3) session-specific metadata (date and behavioral context); (4) probe channel maps; and (5) PETHs for lever presses, lever releases, and port nose pokes. These data formed the input feature set for DANT's tracking pipeline.

Unit localization

The method for 3D localization of units adapted a monopolar current source model.[24] Each unit's 3D position is denoted as x, y, z. Channel positions x_c, y_c on the probe plane follow SpikeGLX/Kilosort conventions, defining the position of a channel relative to the probe tip, with $z_c = 0$ (fixed at the probe's two-dimensional [2D] plane). Using a monopolar current source model, each unit's position was inferred via

$$\underset{x,y,z,\alpha}{\text{argmin}} \sum_{c \in \mathcal{C}} \left(\text{ptt}_c - \frac{\alpha}{\sqrt{(x - x_c)^2 + (y - y_c)^2 + z^2}} \right)^2, \quad \text{(Equation 1)}$$

where ptt_c is the peak-to-trough amplitude of the spike waveform on channel c, $\mathcal{C}$ represents the indices of the $n = 20$ nearest channels to the peak channel (with maximum peak-to-trough amplitude), and α is the signal magnitude scaling factor. The optimization was performed using MATLAB's "lsqcurvefit" function. The resulting position (x, y, z) denotes the relative position of each unit to the probe tip and was used to estimate probe motion, informing the motion correction algorithm described next.

Probe-motion estimation and correction

The method estimated probe motion across recording sessions using the location data of matched unit pairs, identified by the clustering algorithm described below. Let N_s be the total number of sessions, N_p the number of matched unit pairs, and p_s the scalar probe position (depth of the probe) in session s ($s = 1, \ldots, N_s$; Figure 2A). For the ith matched pair ($i = 1, \ldots, N_p$), let s_A^i and s_B^i denote the sessions from which the two matched units were recorded, with y_A^i and y_B^i representing their spatial positions along the probe's primary axis (relative to the probe tip, with $x_A^i, x_B^i, z_A^i, z_B^i$ ignored; Figure 2A). The probe position vector across sessions $\mathbf{p} = (p_1, \ldots, p_{N_s})$ was estimated by solving

$$\mathbf{p}^* = \underset{\mathbf{p}}{\text{argmin}} \sum_{i=1}^{N_p} \left[\left(y_A^i - y_B^i \right) - \left(p_{s_A^i} - p_{s_B^i} \right) \right]^2. \quad \text{(Equation 2)}$$

This optimization minimizes the mean squared relative displacement of matched units in the brain if the probe position is corrected using $\mathbf{p}^*$. The optimization was performed using the "fminunc" function in MATLAB Optimization Toolbox. Estimated probe positions were then converted to relative values by subtracting either the first session's position ($\mathbf{p}^* - p_1^*$) or the mean position across sessions ($\mathbf{p}^* - \overline{\mathbf{p}^*}$), which were used for waveform correction and refined waveform correlation calculation.

DREDge,[25] a robust algorithm for registering extracellular electrophysiology recordings, was utilized to validate the probe motion. For the example rat shown in Figure 2, recordings from all sessions were concatenated and processed within the SpikeInterface framework,[55] which implements the DREDge algorithm. For the other example rats shown in Figure S2, which had longer recording sequences, only the first 10 min of each session was concatenated instead of the full recordings. Probe positions for each time point and spatial bin were estimated using the SpikeInterface "compute_motion" function with the "dredge" preset. The median of these estimated positions across all time and spatial bins was taken as the single, representative probe position for that session. To assess spike alignment, spikes detected by DREDge were binned in 5-s time intervals and 5-μm vertical spatial bins. The mean spike amplitude in each spatiotemporal bin was calculated. These values were plotted as a heatmap (Figures S2B–S2D and S2F–S2H) to visualize the distribution of spike activity along the probe shank over time.

Waveform correction

Let $\mathbf{W} \in \mathbb{R}^{n_c \times n_t}$ denote the matrix of the original average waveforms, where n_c is the number of channels and n_t is the number of time samples per waveform. Channel positions were represented in a 2D coordinate system, with x denoting the lateral position and y denoting the depth along the probe shank. The original channel positions were collected as $\mathbf{v}_1 = \{[x_{1,i}, y_{1,i}]\}, i = 1, \ldots, n_c$, where $\mathbf{x}_1$ and $\mathbf{y}_1$ are two vectors containing the x and y coordinates for each channel. Motion-corrected channel positions are defined as $\mathbf{v}_2 = \{[x_{2,i}, y_{2,i}]\}, i = 1, \ldots, n_c$. Here, the lateral positions remain unchanged ($x_{2,i} = x_{1,i}$), while the depth coordinates are corrected as $y_{2,i} = y_{1,i} - p_j^*$, where p_j^* denotes the estimated vertical displacement in session j. Corrected waveforms, denoted $\tilde{\mathbf{W}}$, were estimated on these motion-corrected channel positions $\mathbf{v}_2$ using Kriging interpolation.[56] To quantify spatial proximity, distance matrices were defined as

$$\mathbf{D}_x(\mathbf{v}_1, \mathbf{v}_2) = \left| \mathbf{x}_1 - \mathbf{x}_2^T \right|, \quad \text{(Equation 3)}$$

$$\mathbf{D}_y(\mathbf{v}_1, \mathbf{v}_2) = \left| \mathbf{y}_1 - \mathbf{y}_2^T \right|, \quad \text{(Equation 4)}$$

where $\mathbf{D}_x(\mathbf{v}_1, \mathbf{v}_2)$ and $\mathbf{D}_y(\mathbf{v}_1, \mathbf{v}_2)$ represent absolute differences in lateral (x) and depth (y) coordinates, respectively. Spatial similarity was modeled using an exponential kernel,

$$\mathcal{K}(\mathbf{v}_1, \mathbf{v}_2) = \exp\left(-\frac{\mathbf{D}_x(\mathbf{v}_1, \mathbf{v}_2)}{\sigma_x} - \frac{\mathbf{D}_y(\mathbf{v}_1, \mathbf{v}_2)}{\sigma_y} \right), \quad \text{(Equation 5)}$$

with spatial scale parameters $\sigma_x = 20$ and $\sigma_y = 30$ in μm. Finally, motion-corrected waveforms $\tilde{\mathbf{W}}$ at channel positions $\mathbf{v}_2$ were obtained via

$$\tilde{\mathbf{W}} = \mathcal{K}(\mathbf{v}_2, \mathbf{v}_1) \mathcal{K}(\mathbf{v}_1, \mathbf{v}_1)^{-1} \mathbf{W}. \quad \text{(Equation 6)}$$

Similarity scores

Waveforms

Waveform similarity between units was computed using raw or motion-corrected waveforms $\mathbf{W}$ from the n nearest channels (default $n = 38$) to the channel with the largest amplitude for each unit. The similarity was quantified using Pearson's correlation coefficient, followed by Fisher's z-transformation. For units i and j, the n nearest channels to the largest-amplitude channel are denoted $\mathcal{C}_i$ and $\mathcal{C}_j$, respectively, based on their 2D spatial

coordinates on the probe. The z-transformed correlations were computed as

$$\mathcal{Z}_{i,j} = \tanh^{-1}\left(\text{corrcoef}\left(\mathbf{W}^i_{\mathcal{C}_i}, \mathbf{W}^j_{\mathcal{C}_i}\right)\right), \quad \text{(Equation 7)}$$

$$\mathcal{Z}_{j,i} = \tanh^{-1}\left(\text{corrcoef}\left(\mathbf{W}^i_{\mathcal{C}_j}, \mathbf{W}^j_{\mathcal{C}_j}\right)\right), \quad \text{(Equation 8)}$$

where $\mathbf{W}^l_{\mathcal{C}_k}$ ($l,k \in \{i,j\}$) represents the waveforms of unit l on the channels indexed by $\mathcal{C}_k$. A symmetric similarity score was obtained by

$$\mathbf{S}^{i,j}_{\text{WF}} = \max\left(\mathcal{Z}_{i,j}, \mathcal{Z}_{j,i}\right). \quad \text{(Equation 9)}$$

Autocorrelogram

The autocorrelogram for each unit was calculated with a maximum lag of 300 ms and a bin width of 1 ms. The resulting distribution was smoothed using a Gaussian kernel with a standard deviation of 5 ms and set to zero at lag 0. The autocorrelogram similarity score between units i and j was given by

$$\mathbf{S}^{i,j}_{\text{ACG}} = \tanh^{-1}\left(\text{corrcoef}\left(\mathbf{ACG}_i, \mathbf{ACG}_j\right)\right). \quad \text{(Equation 10)}$$

The usefulness of ACG-based features depends on firing rate, recording duration, and the temporal scale at which the ACG is evaluated. Under a simple stationary Poisson approximation, the expected number of spike pairs contributing to an effective lag window of width w scales as Nrw, where N is spike count and r is mean firing rate. If the ACG rate is estimated from these counts, its variance scales as $r/(Nw)$, giving a standard error of $\sqrt{r/(Nw)}$. Using a normal approximation, the corresponding 95% confidence interval is approximately $r \pm 1.96\sqrt{r/(Nw)}$. Thus, low firing rates and short recordings lead to noisy ACG estimates, especially for fine-timescale features. For example, at a firing rate of 1 Hz and an ACG bin width of 1 ms, obtaining a 95% confidence interval of approximately 1 ± 0.25 Hz would require on the order of 6.2×10^4 spikes, corresponding to about 17 h of recording. In comparison, at a firing rate of 10 Hz, obtaining a 95% confidence interval of approximately 10 ± 2.5 Hz would require about 6.2×10^3 spikes, corresponding to about 10 min of active spiking. Furthermore, smoothing or using broader-timescale ACG features effectively increases the lag-window width and can substantially reduce this requirement. Thus, ACG-based features are most informative for moderately to highly active neurons or for features evaluated at broader timescales, whereas very low firing rates combined with short recordings may limit their contribution to matching.

PETH

PETHs for each unit were precomputed during data processing, aligned to specific events in a forelimb-dependent SRT task studied here. Three PETHs, corresponding to press, release, and poke events, were calculated with a time window of ± 500 ms around each event, using a bin width of 1 ms, yielding 1,000 bins per PETH. The feature vector for unit i, denoted PETH_i, was formed by concatenating these three PETHs into a vector of length 3,000. The PETH similarity score between units i and j was given by

$$\mathbf{S}^{i,j}_{\text{PETH}} = \tanh^{-1}\left(\text{corrcoef}\left(\mathbf{PETH}_i, \mathbf{PETH}_j\right)\right). \quad \text{(Equation 11)}$$

Weighted similarity score

The weighted similarity score between units i and j combined waveform ($\mathbf{S}_{\text{WF}}$), autocorrelogram ($\mathbf{S}_{\text{ACG}}$), and PETH ($\mathbf{S}_{\text{PETH}}$) similarity scores as a weighted average:

$$\mathbf{S} = w_{\text{WF}}\mathbf{S}_{\text{WF}} + w_{\text{ACG}}\mathbf{S}_{\text{ACG}} + w_{\text{PETH}}\mathbf{S}_{\text{PETH}}, \quad \text{(Equation 12)}$$

where weights $w_{\text{WF}}, w_{\text{ACG}}$, and w_{PETH} summed to 1, were initially equal (1/3), and were optimized for clustering accuracy. If PETH features were omitted due to task absence or significant response drift, the score simplified to

$$\mathbf{S} = w_{\text{WF}}\mathbf{S}_{\text{WF}} + w_{\text{ACG}}\mathbf{S}_{\text{ACG}}, \quad \text{(Equation 13)}$$

with $w_{\text{WF}} + w_{\text{ACG}} = 1$.

HDBSCAN

HDBSCAN is an unsupervised, hierarchical clustering algorithm based on the density of data.[19,26] This algorithm identifies groups of data based on local density profiles. To build a minimal spanning tree, HDBSCAN relies on the "mutual reachability distance," defined as

$$d_{\text{mreach}}(x_i, x_j) = \max\left\{\mathbf{core}_k(x_i), \mathbf{core}_k(x_j), d(x_i, x_j)\right\}, \quad \text{(Equation 14)}$$

where $\text{core}_k(x_i)$ is the distance to the kth nearest neighbor of unit x_i, and $d(x_i, x_j)$ is a base distance (e.g., Euclidean). This metric ensures a density-dependent lower bound. To maximize sensitivity to small dense groups of units (including units from only two sessions), DANT sets $k = 1$.

In this study, the weighted similarity score $\mathbf{S}$ was transformed into a distance metric for HDBSCAN using the following heuristic formula:

$$\mathbf{D}_{i,j} = \begin{cases} 0 & \text{if } i = j, \\ \dfrac{1}{1 + \tanh\left(\mathbf{S}_{i,j}\right)} & \text{otherwise} \end{cases}. \quad \text{(Equation 15)}$$

Here, $\mathbf{S}_{i,j} \in \mathbb{R}$ maps to $\tanh(\mathbf{S}_{i,j}) \in (-1, 1)$, yielding $\mathbf{D}_{i,j} \in (0.5, \infty)$ for $i \neq j$, with smaller distances approaching 0.5 for higher similarities. Chosen for its practical effectiveness in producing meaningful clusters in the context of chronic Neuropixels data, this heuristic transformation maps similarity scores to a distance metric suitable for HDBSCAN. The use of the hyperbolic tangent function ensures a smooth, bounded transformation, effectively capturing the relative differences in similarity for clustering units based on waveform or functional features.

The Python implementation of HDBSCAN[27,57] was used, with parameters set to a minimum cluster size of 2 to allow small unit groups, a maximum cluster size equal to the total number of recording sessions, and minimum samples of 1 to maximize sensitivity to dense regions.

The resulting single-linkage tree was optimized using MATLAB's "optimalleaforder" function to reorder units for visualization (Figures 3F and 3G). For further visualization, UMAP was applied to the precomputed distance matrix $\mathbf{D}_{i,j}$, producing a 2D embedding that preserves local structure (Figure 3H). UMAP parameters were set to a minimum distance of 0.9, spread of 1, and 15 neighbors to optimize separation of clusters.

LDA

The separation boundary between matched and unmatched unit pairs in the feature space was determined using LDA with MATLAB's "fitcdiscr" function. Only unit pairs within 100 μm along the probe's *y* axis were included in this analysis. We assumed that the feature vectors comprising waveform, autocorrelogram, and PETH similarity scores were drawn from multivariate Gaussian distributions with equal covariance matrices for the matched and unmatched classes. LDA yields a hyperplane $\mathbf{w}^{\top}\mathbf{x} + b = 0$ that maximizes separation between these two classes. The discriminant vector $\mathbf{w}$ was normalized such that $\sum_i w_i = 1$, and the bias term b was scaled accordingly. The normalized coefficients of w were then used as weights to compute an optimized similarity score, integrating contributions from waveform, autocorrelogram, and PETH similarities (Equation 12). Similarity scores were projected onto this discriminant axis to distinguish matched from unmatched pairs. The scalar decision threshold, $s_{\text{thres}} = -b$, defined by the scaled hyperplane intercept, was subsequently applied during autocuration of DANT results.

Iterative clustering algorithm

An iterative algorithm was developed to cluster Neuropixels units over n_{iter} iterations (10 iterations by default; Algorithm 1). A connectivity matrix $\mathbf{C}$ ($N_u \times N_u$) was generated by HDBSCAN, with $\mathbf{C}_{a,b} = 1$ indicating matched units. Similarity scores (Equation 12) were computed using default weights, transformed into a distance matrix, and clustered. HDBSCAN's clustering results were used to identify matched and unmatched pair indices, which were then used by LDA to refine the weights. Updated weights were applied to recompute similarity scores for the next iteration, enhancing clustering accuracy.

For each dataset, three steps of iterative clustering (m3–m5), motion correction (m6–m7), and similarity score calculation (m2) were performed (Figure 1). Different feature subsets were selected for each step.

- Step 1: $\mathbf{S}_{\text{ACG}} + \mathbf{S}_{\text{PETH}}$. This mitigates the impact of large probe drift between sessions, which could otherwise yield too few matched units.
- Steps 2 and 3: $\mathbf{S}_{\text{ACG}} + \mathbf{S}_{\text{PETH}} + \mathbf{S}_{\text{WF}}$. This incorporates all features to optimize motion correction.

For final clustering, similarity scores were based only on $\mathbf{S}_{\text{ACG}} + \mathbf{S}_{\text{WF}}$, omitting $\mathbf{S}_{\text{PETH}}$ to improve matching of units with significant functional drift.

Steps 1–3 were designed to infer probe motion and correct spike waveforms accordingly. The underlying assumption was that some units exhibited stable functional properties, reflected in $\mathbf{S}_{\text{PETH}}$ and $\mathbf{S}_{\text{ACG}}$, enabling robust motion correction in step 1 to prevent large probe drift in a handful of sessions from disrupting clustering of continuously recorded neurons. Units with significant functional drift or minimal behavioral modulation contributed less to motion correction but were still likely matched in the final clustering step.

Autocuration

Clustering results occasionally violated the constraint that units within a cluster must originate from distinct recording sessions. Units within each cluster were therefore inspected for session uniqueness, and if multiple units from the same session were detected, those with lower pairwise similarity scores to other units were removed.

Additionally, as the clustering algorithm prioritizes cluster stability over strict unit similarity, it can group units with low similarity. To ensure high-quality matching, pairwise similarity scores between all unit pairs within each cluster were evaluated, and the similarity threshold derived from LDA was applied. A connection was established between two units if their similarity score exceeded this threshold. Treating the resulting structure as a graph, its connected components were identified. If more than one component was detected within a cluster, it was split into subclusters corresponding to these components.

Manual curation

To facilitate visualization and curation of clusters identified by DANT, we developed a Python-based program with a GUI (available at https://github.com/jiumao2/DANT_UI). This tool extracts multiple features for each unit to enable systematic comparison, including motion-corrected waveforms, ACGs, PETHs, weighted similarity scores between units, and a custom figure illustrating the unit's functional tuning within a given session (rasters and PETHs aligned to several events and separated by trial outcomes). The interface allows users to identify outlier units within clusters and reassign them to subclusters. In addition, it enables inspection of each cluster alongside its most similar clusters, based on the maximum weighted similarity score across units, to guide decisions on cluster merging.

Using this program, we manually curated clusters from eight datasets, comprising recordings from motor cortex ($n = 6$) and dorsolateral striatum ($n = 2$). Assuming functional properties remain consistent across neighboring days, units exhibiting clearly different functional characteristics were excluded from their original clusters.

Neural activity stability across days

To assess the stability of lift-related neural activity across multiple days (Figure 4E), PETHs were computed for individual neurons. For the example rat presented in Figure 4, PETHs were generated for neural activity around the peak of paw lift (from −1 s to 0.5 s relative to peak lift). These PETHs were constructed with 10-ms bins and smoothed with a Gaussian kernel with a standard deviation of 50 ms. Only neurons reliably tracked across a minimum of ten experimental sessions were included in this analysis ($n = 152$). To include only neurons significantly modulated around paw lifting, first, neurons with a mean firing rate below 5 Hz during the specified window were excluded. Second, a shuffling-based modulation test was performed for each neuron. Spikes were binned at 50-ms resolution around the lift-highest points (−1 s to +0.5 s) for each trial, and a PETH was computed by averaging spike counts across trials. The mean spike count across all bins and trials served as the baseline. Next, a random circular shuffling of spike counts was applied independently to each trial. A new PETH was computed from the shuffled data, and the summed squared difference from the baseline was calculated. This procedure was repeated 1,000

Algorithm 1. Iterative HDBSCAN with step-specific feature selection and autocuration

Hyperparameters: $n_{step} = 3, n_{iter} = 10$ ▷ Set default iteration limits
Initialize default weights: $w_{WF} = 0, w_{ACG} = \frac{1}{2}, w_{PETH} = \frac{1}{2}$ for Step 1; $w_{WF} = \frac{1}{3}, w_{ACG} = \frac{1}{3}, w_{PETH} = \frac{1}{3}$ for Steps 2–3 ▷ Set step-specific weights
for $i = 1$ to n_{step} **do**
  if $i = 1$ **then**
    $\mathbf{S} = w_{ACG}\mathbf{S}_{ACG} + w_{PETH}\mathbf{S}_{PETH}$ ▷ Compute similarity score for Step 1 (Equation 12)
  else
    $\mathbf{S} = w_{WF}\mathbf{S}_{WF} + w_{ACG}\mathbf{S}_{ACG} + w_{PETH}\mathbf{S}_{PETH}$ ▷ Compute similarity score for Steps 2–3 (Equation 12)
  end if
  for $j = 1$ to n_{iter} **do**
    $\mathbf{D} = 1/(1 + \tanh(\mathbf{S}))$ ▷ Transform similarity scores to distance matrix
    $\mathbf{C} = \mathrm{HDBSCAN}(\mathbf{D})$ ▷ Cluster units using HDBSCAN
    $\mathbf{I}_{matched}$ = indices where $\mathbf{C}_{a,b} = 1$ and $a < b$ ▷ Identify matched pair indices
    $\mathbf{I}_{unmatched}$ = indices where $\mathbf{C}_{a,b} = 0$ and $a < b$ ▷ Identify unmatched pair indices
    if $i = 1$ **then**
      $[w_{ACG}, w_{PETH}] = \mathrm{LDA}(\mathbf{S}_{ACG}, \mathbf{S}_{PETH}, \mathbf{I}_{matched}, \mathbf{I}_{unmatched})$ ▷ Update weights for ACG, PETH in Step 1
      $\mathbf{S} = w_{ACG}\mathbf{S}_{ACG} + w_{PETH}\mathbf{S}_{PETH}$ ▷ Recompute similarity score for Step 1
    else
      $[w_{WF}, w_{ACG}, w_{PETH}] = \mathrm{LDA}(\mathbf{S}_{WF}, \mathbf{S}_{ACG}, \mathbf{S}_{PETH}, \mathbf{I}_{matched}, \mathbf{I}_{unmatched})$ ▷ Update weights for WF, ACG, PETH in Steps 2–3
      $\mathbf{S} = w_{WF}\mathbf{S}_{WF} + w_{ACG}\mathbf{S}_{ACG} + w_{PETH}\mathbf{S}_{PETH}$ ▷ Recompute similarity score for Steps 2–3
    end if
  end for
  Estimate probe motion across sessions using matched unit positions ▷ Motion correction (Equation 2)
  Remap waveforms to probe sites based on estimated positions and motion ▷ Waveform correction (Equation 6)
end for
$\mathbf{S} = \frac{1}{2}\mathbf{S}_{WF} + \frac{1}{2}\mathbf{S}_{ACG}$ ▷ Compute final similarity score, omitting $\mathbf{S}_{PETH}$
for $j = 1$ to n_{iter} **do**
  $\mathbf{D} = 1/(1 + \tanh(\mathbf{S}))$ ▷ Transform to distance matrix
  $\mathbf{C} = \mathrm{HDBSCAN}(\mathbf{D})$ ▷ Cluster units using HDBSCAN
  $\mathbf{I}_{matched}$ = indices where $\mathbf{C}_{a,b} = 1$ and $a < b$ ▷ Identify matched pair indices
  $\mathbf{I}_{unmatched}$ = indices where $\mathbf{C}_{a,b} = 0$ and $a < b$ ▷ Identify unmatched pair indices
  $[w_{WF}, w_{ACG}] = \mathrm{LDA}(\mathbf{S}_{WF}, \mathbf{S}_{ACG}, \mathbf{I}_{matched}, \mathbf{I}_{unmatched})$ ▷ Update weights for WF, ACG
  $\mathbf{S} = w_{WF}\mathbf{S}_{WF} + w_{ACG}\mathbf{S}_{ACG}$ ▷ Recompute similarity score
end for
for each cluster in $\mathbf{C}$ **do**
  Remove units from the same session, keeping the unit with the highest average pairwise similarity score ▷ Ensure session uniqueness (Figure S4A)
  Split cluster if subsets have higher pairwise similarity scores ▷ Ensure high-quality matching (Figure S4H)
end for
Assign cluster IDs to matched units ▷ Output final matched units

times to generate a null distribution of modulation scores. The p value for each session was defined as the fraction of shuffled scores that were greater than or equal to the observed score. To combine p values across n sessions for each neuron, Fisher's method was applied. Under the null hypothesis that PETHs from all n sessions are unmodulated (i.e., all p values are random variables uniformly distributed on [0, 1]), the Fisher statistic $-2\sum \ln(p)$ follows a chi-squared distribution with $2 \times n$ degrees of freedom.[58] Clusters with combined p values below 0.01 were included (60 out of 152).

For each included neuron, pairwise Pearson correlation coefficients were calculated between its PETHs from every possible pair of sessions as a function of the temporal separation (in days) between those sessions. The mean and standard deviation of these correlation coefficients, aggregated across the remaining neurons ($n = 60$), are presented in Figure 4E.

Time warping

Piecewise time warping was applied to align PETHs for lever press, lever release, and port nose poke on a common time axis (Figure S5F). For each trial i, let t_1^i, t_2^i, and t_3^i be the time stamps of the three behavioral events relative to lever press (so that $t_1^i = 0$). The median latencies of lever release and port nose poke relative to lever press are denoted as $\bar{t}_2$ and $\bar{t}_3$, and $\bar{t}_1$ is set to 0.

Spikes were binned at 1-ms resolution, producing spike counts $x_{i,j}$ at raw times $\tau_{i,j}$ in trial i and bin j. Within each segment

(k = 1 for press to release, k = 2 for release to poke), any raw time $\tau_{i,j} \in [t^i_k, t^i_{k+1}]$ was linearly mapped to a warped time $\tau'_{i,j}$ via

$$\tau'_{i,j} = \bar{t}_k + (\tau_{i,j} - t^i_k)\frac{\bar{t}_{k+1} - \bar{t}_k}{t^i_{k+1} - t^i_k}. \quad \text{(Equation 16)}$$

The warped spike pairs $(\tau'_{i,j}, x_{i,j})$ were linearly interpolated onto a common time grid using MATLAB's "interp1" function. The time-warped PETH was then obtained by averaging the interpolated spike counts across trials and subsequently smoothing with a Gaussian kernel with a standard deviation of 50 ms.

Comparison with UnitMatch and EMD

The example scripts provided in the GitHub repositories for UnitMatch (https://github.com/EnnyvanBeest/UnitMatch; MATLAB version) and EMD (https://github.com/janelia-TDHarrisLab/Yuan-Neuron_Tracking) were used for neuron tracking. Analyses were restricted to the same populations of well-isolated units across all methods. Default parameters for each method were applied across datasets without fine-tuning. For DANT, PETH features were excluded throughout the pipeline to ensure fair comparison. For UnitMatch, average waveforms for each unit were additionally extracted from the first and second halves of each session, as required by the algorithm. The "default" version of the UnitMatch algorithm was used to track the neurons over many recordings. UnitMatch failed in rat #2 (16,607 single units) and rat #4 (14,748 single units) due to an out-of-memory error, and these datasets were truncated to the first 31 sessions for successful processing. EMD encountered an error in rat #6, and this dataset was excluded from further comparison. In total, 12 datasets were used for comparative analysis. All computations were performed on a computer with a 20-core Intel Xeon Silver 4114 CPU and 64 GB RAM.

To compare the PETH similarity of matched unit pairs recorded on consecutive days, similarity scores were calculated for matched pairs (Equation 11). Units with a mean firing rate below 1 Hz or a peak firing rate below 5 Hz in the PETH were excluded, since such pairs were more likely to yield artificially low similarity. Datasets with fewer than 100 valid matched unit pairs detected by any of the three methods were also excluded to ensure stable estimation of median similarity. The Friedman test was then applied to assess significant differences in PETH similarity across methods.

PETH similarity for unmatched unit pairs was evaluated as an indicator of potential false negatives. To establish a null distribution, we calculated PETH similarity for unit pairs recorded across consecutive days that were separated by more than 50 μm, which were assumed to represent correctly unmatched pairs. A Gaussian function was then fitted to this null distribution, yielding parameters μ = 0.025 and σ = 0.450. A one-sided threshold was then defined such that less than 0.01% of the null distribution exceeded it, corresponding to a similarity score of 1.70. Unit pairs with similarity above this threshold were classified as outliers. Subsequently, unmatched pairs were grouped into 20-μm distance bins. For each method, the proportion of outlier unmatched pairs within each bin was computed. Statistical comparisons of these proportions across methods were performed using chi-squared tests and, when a significant difference in distribution was found in a bin, pairwise Fisher's exact tests were performed to compare different methods.

To further validate the tracking results, a subset of ten high-quality, two-session recordings was selected from five rats. Data from these sessions were concatenated and jointly spike sorted using Kilosort 2.5. For each unit identified in the individual session sortings, a corresponding unit in the combined dataset was sought. Units were considered "included" if more than 50% of their spike times (difference ≤1 ms) overlapped with a unit from the combined sorting. The majority of single units (87.9% ± 14.2%, mean ± SD) were successfully detected in this combined sorting output. For these identified overlapping units, those assigned to the same cluster by Kilosort were defined as a "match." These Kilosort-derived matches served as the reference standard for validating matches found by DANT, UnitMatch, and EMD. Precision and recall were then used to quantify the agreement with Kilosort-derived matches. Precision was the proportion of detected matches that were also detected by Kilosort, whereas recall was the proportion of Kilosort matches that were detected by each method:

$$\text{Precision} = \frac{TP}{TP + FP}, \quad \text{(Equation 17)}$$

$$\text{Recall} = \frac{TP}{TP + FN}, \quad \text{(Equation 18)}$$

where TP is the number of matches identified by both Kilosort and the evaluated method, FP is the number of matches found exclusively by the evaluated method, and FN is the number of matches found exclusively by Kilosort.

To evaluate false-positive match rates, a synthetic dataset was constructed from a 7-day subset of data from rat #3. These data were recorded using a Neuropixels 2.0 probe during a period of minimal probe motion. The same regions on two of the probe's four shanks were consistently recorded across the 7-day experiment. These two shanks were 250 μm apart, thus minimizing the likelihood of recording from identical neurons. To simulate false-positive matches, units recorded from these two shanks on the same day were artificially assigned to separate "days" within the synthetic dataset, resulting in a 14-day dataset. DANT and UnitMatch were then applied to the synthetic dataset. False-positive matches were defined as pairs of units originating from different shanks.

Tracking neurons across days in mouse visual cortex

A reanalysis of a publicly available dataset from Lebedeva et al.[3,59] was performed for this study. In contrast to the aforementioned rat datasets, recordings in this mouse dataset were not conducted daily but rather at typical weekly intervals. During each recording session, mice were head-fixed in front of three visual displays (left, center, and right). Visual stimuli were presented for 1 s on the center and right screens, whereas the left screen remained gray. During the inter-stimulus interval of at least 2 s, all screens were maintained at gray. Each session included the presentation of 112 natural images, with each image repeated five times, yielding a total of 560 stimulus presentations per session.

This dataset includes pyKilosort spike-sorting output (https://github.com/int-brain-lab/pykilosort) from chronic recordings in the primary visual cortex of three mice. One session from mouse #2 (20200219) was excluded because the channel map utilized for that recording differed from those of the other sessions. Only units labeled as "good" by the spike sorter were included in the subsequent analysis. Following the same general pipeline established for the rat datasets, we compiled for each unit (1) mean waveforms across all recorded channels; (2) spike times; (3) session-specific metadata, including recording date and behavioral context; (4) probe channel maps; and (5) a visual fingerprint, defined as a 112-dimensional vector containing the mean spike-count response to each image. These variables constituted the input to the DANT tracking pipeline. DANT was then applied to track neurons across days, using the visual fingerprint together with waveform and ACG features during non-rigid motion correction. During the final clustering step, only waveform and ACG features were used.

To evaluate the stability of DANT-identified clusters across recording sessions, the analytical framework previously established by Steinmetz et al.[3] was applied. In brief, we selected units with significant visual responses across the 112 natural images ($p < 0.01$, Kruskal-Wallis test) and DANT-identified matched unit pairs whose baseline firing rates differed by no more than 4-fold between the two sessions. Unit pairs satisfying both criteria were considered to have reliable visual fingerprints. Finally, session pairs with fewer than 20 valid matches were excluded to ensure reliable estimation. The application of these criteria resulted in the final inclusion of 7,284 matched pairs derived from three animals, 25 sessions, and 2,905 units.

The visual fingerprint for this analysis consisted of two components: the mean spike-count response to each image, calculated within the 1-s stimulus-presentation window, and the mean temporal response profile across all images. This temporal profile was computed from PSTHs spanning 0.5 s before stimulus onset to 0.5 s after stimulus offset, smoothed with a 50-ms Gaussian kernel. For each unit, the visual fingerprint from the earlier session was compared both with its own visual fingerprint from the later session and with that of another unit recorded in the later session whose location was closest to that of the original unit. Similarity between two visual fingerprints was quantified as the average of two Pearson correlation coefficients: one computed from the image-evoked spike counts across the 112 images and the other from the corresponding PSTHs.

A comparison was classified as a match when the fingerprint from the earlier session was more similar to that of the same unit in the later session than to that of the nearest neighboring unit; otherwise, it was classified as a mismatch. To account for chance-level matching probabilities, the estimated probability of stability was calculated as $2\,\mathrm{Pr}(\mathrm{match}) - 1$, where $\mathrm{Pr}(\mathrm{match})$ represents the proportion of matches.

Visual fingerprint similarity for unmatched unit pairs was similarly evaluated as in the rat datasets to evaluate false negatives. The null distribution was established using fingerprint similarity for unit pairs from sessions separated by 7 days or less and spatially separated by more than 50 μm. A Gaussian function was fitted with parameters $\mu = 0.177$ and $\sigma = 0.373$. Because fewer pairs were available for this analysis, we used a less stringent threshold ($\alpha = 0.1\%$, versus 0.01% for the rat datasets), corresponding to a similarity score of 1.33, above which less than 0.1% of the null distribution fell. The subsequent analyses for the outlier rates and statistical tests were the same as for the rat datasets.

Statistics

All statistical analyses were performed in R. When comparing units' tracked lengths across methods, a linear mixed-effects model was fitted using the "lme4" package with the formula

$$\mathrm{TrackedLength} \sim \mathrm{MethodNames} + (1 + \mathrm{MethodNames}|\mathrm{DatasetID}) + (1|\mathrm{UnitID}), \quad \text{(Equation 19)}$$

where DatasetID accounted for variability across datasets (including differences in session numbers and unit yields) by allowing both random intercepts and slopes, and UnitID accounted for repeated measures within units. Estimated marginal means were obtained using the "emmeans" package, and pairwise comparisons against DANT were performed with Bonferroni correction. For non-parametric comparisons of PETH similarity, precision, and recall across methods, the Friedman test was conducted using the "PMCMRplus" package followed by Nemenyi post hoc pairwise analysis when appropriate.

RESOURCE AVAILABILITY

Lead contact

Requests for further information and resources should be directed to and will be fulfilled by the lead contact, Jianing Yu (jianing.yu@gmail.com).

Materials availability

This study did not generate new unique reagents.

Data and code availability

Example data for the rat presented in Figures 2, 3, and 4 are available as part of the software demo at figshare: https://doi.org/10.6084/m9.figshare.30596258.v2.[60,61] All additional data and code are available at Zenodo: https://doi.org/10.5281/ZENODO.20019488.[62]

DANT is available as both a MATLAB implementation on GitHub at https://github.com/jiumao2/DANT (GPL-3.0 license)[63] and a Python implementation at https://github.com/jiumao2/pyDANT (GPL-3.0 license).[64] It utilizes the HDBSCAN Python package for clustering[27] (BSD-3-Clause license). Neuropixels data acquisition was performed using SpikeGLX at http://billkarsh.github.io/SpikeGLX/ (Janelia Research Campus Software Copyright 1.2). The Python application for manual curation of DANT results is available at https://github.com/jiumao2/DANT_UI (GPL-3.0 license).[65] Spike sorting was conducted with a modified version of Kilosort 2.5 at https://github.com/jiumao2/Kilosort_2_5 (GPL-2.0 license),[51] followed by manual curation using Phy2 at https://github.com/cortex-lab/phy (BSD-3-Clause license), with custom plugins available at https://github.com/jiumao2/PhyWaveformPlugin (GPL-3.0 license).[52] For a subset of datasets, automated curation was performed using AutoCurationKilosort at https://github.com/jiumao2/AutoCurationKilosort (GPL-3.0 license).[53]

ACKNOWLEDGMENTS

We thank Dr. Jennifer Colonell for discussions and feedback on an early version of the manuscript. Funding for this study was provided by the National Natural Science Foundation of China (32070983, 32271052, and 32571183) (J.Y.), the Natural Science Foundation of Beijing Municipality (5212007)

(J.Y.), the Peking-Tsinghua Center for Life Sciences (J.Y.), and the State Key Laboratory of Membrane Biology (J.Y.).

AUTHOR CONTRIBUTIONS

Conceptualization, Y.H. and J.Y.; methodology, Y.H. and J.Y.; investigation, Y.H., H.W., J.C., Y.C., X.W., Y.Z., H.R., Q.Z., and J.Y.; visualization, Y.H. and J.Y.; supervision, J.Y.; writing – original draft, Y.H. and J.Y.

DECLARATION OF INTERESTS

The authors declare no competing interests.

DECLARATION OF GENERATIVE AI AND AI-ASSISTED TECHNOLOGIES IN THE WRITING PROCESS

The authors used ChatGPT and Claude to improve the readability and clarity of portions of the manuscript. After using these tools, the authors reviewed and edited the content as needed and take full responsibility for the content of the publication.

SUPPLEMENTAL INFORMATION

Supplemental information can be found online at https://doi.org/10.1016/j.patter.2026.101590.

Received: December 8, 2025
Revised: March 25, 2026
Accepted: May 20, 2026
Published: June 17, 2026

REFERENCES

1. Luo, T.Z., Bondy, A.G., Gupta, D., Elliott, V.A., Kopec, C.D., and Brody, C.D. (2020). An approach for long-term, multi-probe Neuropixels recordings in unrestrained rats. eLife *9*, e59716.
2. Juavinett, A.L., Bekheet, G., and Churchland, A.K. (2019). Chronically implanted Neuropixels probes enable high-yield recordings in freely moving mice. eLife *8*, e47188.
3. Steinmetz, N.A., Aydin, C., Lebedeva, A., Okun, M., Pachitariu, M., Bauza, M., Beau, M., Bhagat, J., Böhm, C., Broux, M., et al. (2021). Neuropixels 2.0: A miniaturized high-density probe for stable, long-term brain recordings. Science *372*, eabf4588.
4. Jun, J.J., Steinmetz, N.A., Siegle, J.H., Denman, D.J., Bauza, M., Barbarits, B., Lee, A.K., Anastassiou, C.A., Andrei, A., Aydın, Ç., et al. (2017a). Fully integrated silicon probes for high-density recording of neural activity. Nature *551*, 232–236.
5. Polikov, V.S., Tresco, P.A., and Reichert, W.M. (2005). Response of brain tissue to chronically implanted neural electrodes. J. Neurosci. Methods *148*, 1–18.
6. Böhm, C., and Lee, A.K. (2020). Canonical goal-selective representations are absent from prefrontal cortex in a spatial working memory task requiring behavioral flexibility. eLife *9*, e63035.
7. van Beest, E.H., Bimbard, C., Fabre, J.M.J., Dodgson, S.W., Takács, F., Coen, P., Lebedeva, A., Harris, K.D., and Carandini, M. (2025). Tracking neurons across days with high-density probes. Nat. Methods *22*, 778–787.
8. Fraser, G.W., and Schwartz, A.B. (2012). Recording from the same neurons chronically in motor cortex. J. Neurophysiol. *107*, 1970–1978.
9. Micou, C., and O'Leary, T. (2023). Representational drift as a window into neural and behavioural plasticity. Curr. Opin. Neurobiol. *81*, 102746.
10. Georgopoulos, A.P., Schwartz, A.B., and Kettner, R.E. (1986). Neuronal Population Coding of Movement Direction. Science *233*, 1416–1419.
11. McMahon, D.B.T., Bondar, I.V., Afuwape, O.A.T., Ide, D.C., and Leopold, D.A. (2014). One month in the life of a neuron: Longitudinal single-unit electrophysiology in the monkey visual system. J. Neurophysiol. *112*, 1748–1762.
12. Dickey, A.S., Suminski, A., Amit, Y., and Hatsopoulos, N.G. (2009). Single-Unit Stability Using Chronically Implanted Multielectrode Arrays. J. Neurophysiol. *102*, 1331–1339.
13. Jensen, K.T., Kadmon Harpaz, N., Dhawale, A.K., Wolff, S.B.E., and Ölveczky, B.P. (2022). Long-term stability of single neuron activity in the motor system. Nat. Neurosci. *25*, 1664–1674.
14. Tolias, A.S., Ecker, A.S., Siapas, A.G., Hoenselaar, A., Keliris, G.A., and Logothetis, N.K. (2007). Recording Chronically From the Same Neurons in Awake, Behaving Primates. J. Neurophysiol. *98*, 3780–3790.
15. Schoonover, C.E., Ohashi, S.N., Axel, R., and Fink, A.J.P. (2021). Representational drift in primary olfactory cortex. Nature *594*, 541–546.
16. Chung, J.E., Joo, H.R., Fan, J.L., Liu, D.F., Barnett, A.H., Chen, S., Geaghan-Breiner, C., Karlsson, M.P., Karlsson, M., Lee, K.Y., et al. (2019). High-Density, Long-Lasting, and Multi-region Electrophysiological Recordings Using Polymer Electrode Arrays. Neuron *101*, 21–31.e5.
17. Zhu, H., He, F., Zolotavin, P., Patel, S., Tolias, A.S., Luan, L., and Xie, C. (2025). Temporal coding carries more stable cortical visual representations than firing rate over time. Nat. Commun. *16*, 7162.
18. Yuan, A.X., Colonell, J., Lebedeva, A., Okun, M., Charles, A.S., and Harris, T.D. (2024). Multi-day neuron tracking in high-density electrophysiology recordings using earth mover's distance. eLife *12*, RP92495.
19. Campello, R.J.G.B., Moulavi, D., and Sander, J. (2013). Density-Based Clustering Based on Hierarchical Density Estimates. In Advances in Knowledge Discovery and Data Mining, *7819*, J. Pei, V.S. Tseng, L. Cao, H. Motoda, and G. Xu, eds. (Springer), pp. 160–172.
20. Zheng, Q., Liu, Y., Huang, Y., Cao, J., Wang, X., and Yu, J. (2025). The Role of Striatum in Controlling Waiting during Reactive and Self-Timed Behaviors. J. Neurosci. *45*, e1820242025.
21. Pachitariu, M., Sridhar, S., Pennington, J., and Stringer, C. (2024). Spike sorting with Kilosort4. Nat. Methods *21*, 914–921.
22. Prasad, A., Xue, Q.-S., Sankar, V., Nishida, T., Shaw, G., Streit, W.J., and Sanchez, J.C. (2012). Comprehensive characterization and failure modes of tungsten microwire arrays in chronic neural implants. J. Neural. Eng. *9*, 056015.
23. Prodanov, D., and Delbeke, J. (2016). Mechanical and Biological Interactions of Implants with the Brain and Their Impact on Implant Design. Front. Neurosci. *10*, 11.
24. Boussard, J., Varol, E., Lee, H.D., Dethe, N., and Paninski, L. (2021). Three-dimensional spike localization and improved motion correction for Neuropixels recordings. In Advances in Neural Information Processing Systems, *34* (Curran Associates), pp. 22095–22105.
25. Windolf, C., Yu, H., Paulk, A.C., Meszéna, D., Muñoz, W., Boussard, J., Hardstone, R., Caprara, I., Jamali, M., Kfir, Y., et al. (2025). DREDge: Robust motion correction for high-density extracellular recordings across species. Nat. Methods *22*, 788–800.
26. Campello, R.J.G.B., Kröger, P., Sander, J., and Zimek, A. (2020). Density-based clustering. WIREs Data Min. &. Knowl. *10*, e1343.
27. McInnes, L., Healy, J., and Astels, S. (2017). Hdbscan: Hierarchical density based clustering. J. Open Source Softw. *2*, 205.
28. McInnes, L., Healy, J., and Melville, J. (2020). UMAP: Uniform Manifold Approximation and Projection for Dimension Reduction. Preprint at arXiv. https://doi.org/10.48550/arXiv.1802.03426.
29. Mathis, A., Mamidanna, P., Cury, K.M., Abe, T., Murthy, V.N., Mathis, M.W., and Bethge, M. (2018). DeepLabCut: Markerless pose estimation of user-defined body parts with deep learning. Nat. Neurosci. *21*, 1281–1289.
30. Chestek, C.A., Batista, A.P., Santhanam, G., Yu, B.M., Afshar, A., Cunningham, J.P., Gilja, V., Ryu, S.I., Churchland, M.M., and Shenoy, K.V. (2007). Single-Neuron Stability during Repeated Reaching in Macaque Premotor Cortex. J. Neurosci. *27*, 10742–10750.
31. Okun, M., Lak, A., Carandini, M., and Harris, K.D. (2016). Long Term Recordings with Immobile Silicon Probes in the Mouse Cortex. PLoS One *11*, e0151180.

CellPress
OPEN ACCESS

32. Greenberg, P.A., and Wilson, F.A.W. (2004). Functional Stability of Dorsolateral Prefrontal Neurons. J. Neurophysiol. *92*, 1042–1055.
33. Stevenson, I.H., and Kording, K.P. (2011). How advances in neural recording affect data analysis. Nat. Neurosci. *14*, 139–142.
34. Chaure, F.J., Rey, H.G., and Quian Quiroga, R. (2018). A novel and fully automatic spike-sorting implementation with variable number of features. J. Neurophysiol. *120*, 1859–1871.
35. Hilgen, G., Sorbaro, M., Pirmoradian, S., Muthmann, J.-O., Kepiro, I.E., Ullo, S., Ramirez, C.J., Puente Encinas, A., Maccione, A., Berdondini, L., et al. (2017). Unsupervised Spike Sorting for Large-Scale, High-Density Multielectrode Arrays. Cell Rep. *18*, 2521–2532.
36. Kadir, S.N., Goodman, D.F.M., and Harris, K.D. (2014). High-dimensional cluster analysis with the masked EM algorithm. Neural Comput. *26*, 2379–2394.
37. Saif-ur Rehman, M., Ali, O., Dyck, S., Lienkämper, R., Metzler, M., Parpaley, Y., Wellmer, J., Liu, C., Lee, B., Kellis, S., et al. (2021). Spikedeep-classifier: a deep-learning based fully automatic offline spike sorting algorithm. J. Neural. Eng. *18*, 016009.
38. Toosi, R., Akhaee, M.A., and Dehaqani, M.-R.A. (2021). An automatic spike sorting algorithm based on adaptive spike detection and a mixture of skew-t distributions. Sci. Rep. *11*, 13925.
39. Quiroga, R.Q., Nadasdy, Z., and Ben-Shaul, Y. (2004). Unsupervised Spike Detection and Sorting with Wavelets and Superparamagnetic Clustering. Neural Comput. *16*, 1661–1687.
40. Yger, P., Spampinato, G.L., Esposito, E., Lefebvre, B., Deny, S., Gardella, C., Stimberg, M., Jetter, F., Zeck, G., Picaud, S., et al. (2018). A spike sorting toolbox for up to thousands of electrodes validated with ground truth recordings in vitro and in vivo. eLife *7*, e34518.
41. Jun, J.J., Mitelut, C., Lai, C., Gratiy, S.L., Anastassiou, C.A., and Harris, T.D. (2017b). Real-time spike sorting platform for high-density extracellular probes with ground-truth validation and drift correction. Preprint at bioRxiv. https://doi.org/10.1101/101030.
42. Suárez-Barrera, D., Bayones, L., Encinas-Rodríguez, N., Parra, S., Monroy, V., Pujalte, S., Andrade-Ortega, B., Díaz, H., Alvarez, M., Zainos, A., et al. (2025). Efficient and reliable spike sorting from neural recordings with UMAP-based unsupervised nonlinear dimensionality reduction. PLoS Biol. *23*, e3003527.
43. Dhawale, A.K., Poddar, R., Wolff, S.B., Normand, V.A., Kopelowitz, E., and Ölveczky, B.P. (2017). Automated long-term recording and analysis of neural activity in behaving animals. eLife *6*, e27702.
44. Agarwal, S., Qiu, W., Harris, K.D., Van Beest, E., and Bimbard, C. (2026). DeepUnitMatch: Tracking neurons across days in electrophysiology using Deep Neural Networks. Preprint at bioRxiv. https://doi.org/10.64898/2026.01.30.702777.
45. Lewicki, M.S. (1998). A review of methods for spike sorting: The detection and classification of neural action potentials. Network *9*, R53–R78.
46. Muller, R.U., and Kubie, J.L. (1987). The effects of changes in the environment on the spatial firing of hippocampal complex-spike cells. J. Neurosci. *7*, 1951–1968.
47. Wilson, M.A., and McNaughton, B.L. (1993). Dynamics of the Hippocampal Ensemble Code for Space. Science *261*, 1055–1058.
48. Vöröslakos, M., Petersen, P.C., Vöröslakos, B., and Buzsáki, G. (2021). Metal microdrive and head cap system for silicon probe recovery in freely moving rodent. eLife *10*, e65859.
49. Duning, T., Kloska, S., Steinsträter, O., Kugel, H., Heindel, W., and Knecht, S. (2005). Dehydration confounds the assessment of brain atrophy. Neurology *64*, 548–550.
50. Verbalis, J.G. (2010). Brain volume regulation in response to changes in osmolality. Neuroscience *168*, 862–870.
51. Huang, Y., and Yu, J. (2026a). Kilosort 2.5 (Modified Fork): Preprocessing Modifications. Zenodo. https://doi.org/10.5281/ZENODO.19973060.
52. Huang, Y., and Yu, J. (2026b). PhyWaveformPlugin: Custom phy2 plugins for manual curation of spike-sorting results. Zenodo. https://doi.org/10.5281/ZENODO.20021262.
53. Huang, Y., and Yu, J. (2026c). AutoCurationKilosort: Automated Curation for Kilosort Output. Zenodo. https://doi.org/10.5281/ZENODO.19973259.
54. Siegle, J.H., Jia, X., Durand, S., Gale, S., Bennett, C., Graddis, N., Heller, G., Ramirez, T.K., Choi, H., Luviano, J.A., et al. (2021). Survey of spiking in the mouse visual system reveals functional hierarchy. Nature *592*, 86–92.
55. Buccino, A.P., Hurwitz, C.L., Garcia, S., Magland, J., Siegle, J.H., Hurwitz, R., and Hennig, M.H. (2020). SpikeInterface, a unified framework for spike sorting. eLife *9*, e61834.
56. Matheron, G. (1963). Principles of geostatistics. Econ. Geol. *58*, 1246–1266.
57. McInnes, L., and Healy, J. (2017). Accelerated hierarchical density based clustering. In 2017 IEEE International Conference on Data Mining Workshops (ICDMW) (IEEE), pp. 33–42.
58. Fisher, R.A. (1992). Statistical Methods for Research Workers. In Breakthroughs in Statistics, S. Kotz and N.L. Johnson, eds. (Springer), pp. 66–70.
59. Lebedeva, A., Okun, M., Krumins, M.K., and Carandini, M. (2026). Chronic Recordings from Neuropixels 2.0 Probes in Mice (University College London). https://doi.org/10.5522/04/24411841.V2.
60. Huang, Y., and Yu, J. (2025a). Example Dataset for DANT Figshare. https://doi.org/10.6084/M9.FIGSHARE.30596258.V2.
61. Huang, Y., and Yu, J. (2025b). Example Dataset for pyDANT Figshare. https://doi.org/10.6084/M9.FIGSHARE.30596303.V2.
62. Huang, Y., and Yu, J. (2026e). Longitudinal chronic Neuropixels recordings from rats during reaction-time and self-timed behaviors. Zenodo. https://doi.org/10.5281/ZENODO.20019488.
63. Huang, Y., and Yu, J. (2026f). DANT: Density-based Across-day Neuron Tracking. Zenodo. https://doi.org/10.5281/ZENODO.19972266.
64. Huang, Y., and Yu, J. (2026g). pyDANT: Python Toolkit for DANT. Zenodo. https://doi.org/10.5281/ZENODO.19972858.
65. Huang, Y., and Yu, J. (2026d). DANT_UI: Manual Curation Tool for DANT Output. Zenodo. https://doi.org/10.5281/ZENODO.19973154.

Patterns, Volume *7*

Supplemental information

Density-based longitudinal neuron tracking in high-density electrophysiological recordings

Yue Huang, Hanbo Wang, Jiaming Cao, Yu Chen, Xuanning Wang, Yujie Zhao, Hengkun Ren, Qiang Zheng, and Jianing Yu

Supplemental Methods

Non-rigid motion correction

Rigid motion correction is often insufficient to capture the full extent of across-session probe displacement. In several datasets, displacement varied markedly with depth along the probe, indicating clear non-rigid motion (Figure S3A,B). For example, in Rat #3, the matched unit pairs identified by DANT exhibited a strong linear relationship between recording depth and relative displacement across sessions (Pearson's r = 0.999; Figure S3B). The estimated displacement difference between the superficial and deep regions exceeded 160 μm (Figure S3C), corresponding to 16.2% tissue expansion. Similar expansion or shrinkage patterns were also observed in other rat and mouse datasets (Figure S3G,H).

Such non-rigid motion can substantially impair neuron tracking because the waveform footprint cannot be accurately corrected with a rigid motion model. In these cases, non-rigid motion correction is therefore required. During spike sorting, a common strategy is to divide the probe into multiple spatial blocks and estimate motion separately within each block. However, this strategy is less suitable for unit-based longitudinal tracking. In contrast to spike-sorting approaches that exploit dense spike activity, cross-session tracking relies on smaller number of matched units, which are often distributed unevenly along the probe. Consequently, block-wise estimation may be unstable in sparsely sampled regions and may not accurately capture smooth depth-dependent motion.

To address these limitations, we developed a modified algorithm for non-rigid motion correction. Following the definition used in rigid motion estimation, we additionally define the depth d^i for the i-th matched unit pair as

$$d^i = \frac{y_A^i + y_B^i}{2}. \tag{1}$$

where y_A^i and y_B^i denote the depths of the matched units in sessions s_A^i and s_B^i, respectively. We then introduce two vectors, $\mathbf{k}$ and $\mathbf{b}$, each of length N_s (the number of sessions), representing depth-dependent motion (slope) and overall probe displacement (offset) across sessions. These parameters are estimated by solving

$$\mathbf{k}^*, \mathbf{b}^* = \arg\min_{\mathbf{k},\mathbf{b}} \sum_{i=1}^{N_p} [(y_A^i - y_B^i) - [(k_{s_A^i}\, d^i + b_{s_A^i}) - (k_{s_B^i}\, d^i + b_{s_B^i})]]^2. \tag{2}$$

This formulation reduces to rigid correction when all elements of $\mathbf{k}$ are zero. To ensure proper minimization, k_1 is fixed at zero, assuming no non-rigid motion in the first session. The solution

$\mathbf{b}^*$ is then re-centered by subtracting the mean of $\mathbf{k}^*\,\bar{d}+\mathbf{b}^*$, ensuring that the mean predicted motion at the average depth is zero across sessions:

$$\tilde{\mathbf{b}}^* = \mathbf{b}^* - \text{mean}(\mathbf{k}^*\,\bar{d} + \mathbf{b}^*), \tag{3}$$

where

$$\bar{d} = \frac{1}{N_p}\sum_{i}^{N_p} d^i. \tag{4}$$

Unlike the rigid case, in which probe position is represented by a single value per session, the estimated probe position $p^i(d)$ at the depth d in session i is given by

$$p^i(d) = k_i^*\,d + \tilde{b}_i^*. \tag{5}$$

The subsequent waveform correction step proceeds as in the rigid case. Applied to Rat #3, this method revealed greater magnitude of motion in regions distal to the probe tip (Figure S3C). Compared with rigid motion correction, the non-rigid algorithm identified 42.2% more matched unit pairs (rigid: 23121 matches, with each neuron tracked for 8.1 ± 5.2 sessions; non-rigid: 32895 matches, with each neuron tracked for 11.1 ± 5.5 sessions, mean ± SD; Figure S3D–F). The matching probability from session 1 to session 18 increased from 0.17 to 0.38 (Figure S3E,F). Together, these results show that non-rigid motion correction substantially improves tracking performance in datasets with depth-dependent probe motion.

Supplemental Figures

Supplementary Figure 1 Probe motions across animals.

Supplementary Figure 2 Comparison of DREDge and DANT for probe motion estimation.

Supplementary Figure 3 Non-rigid motion correction.

Supplementary Figure 4 Autocuration for quality control of HDBSCAN clustering results.

Supplementary Figure 5 Behavioral paradigm, performance, and example neurons recorded across sessions in an M2 recording.

Supplementary Figure 6 Example neurons recorded across sessions in a DLS recording.

Supplementary Figure 7 Tracking performance across animals.

Supplementary Figure 8 Manual curation of DANT results: an example cluster split into two subclusters with different functional properties.

Supplementary Figure 9 Manual curation of DANT results: an example cluster with consistent functional properties.

Supplementary Figure 10 False positives and false negatives identified by manual inspection.

Supplementary Figure 11 Evaluating runtime performance.

Supplementary Figure 1

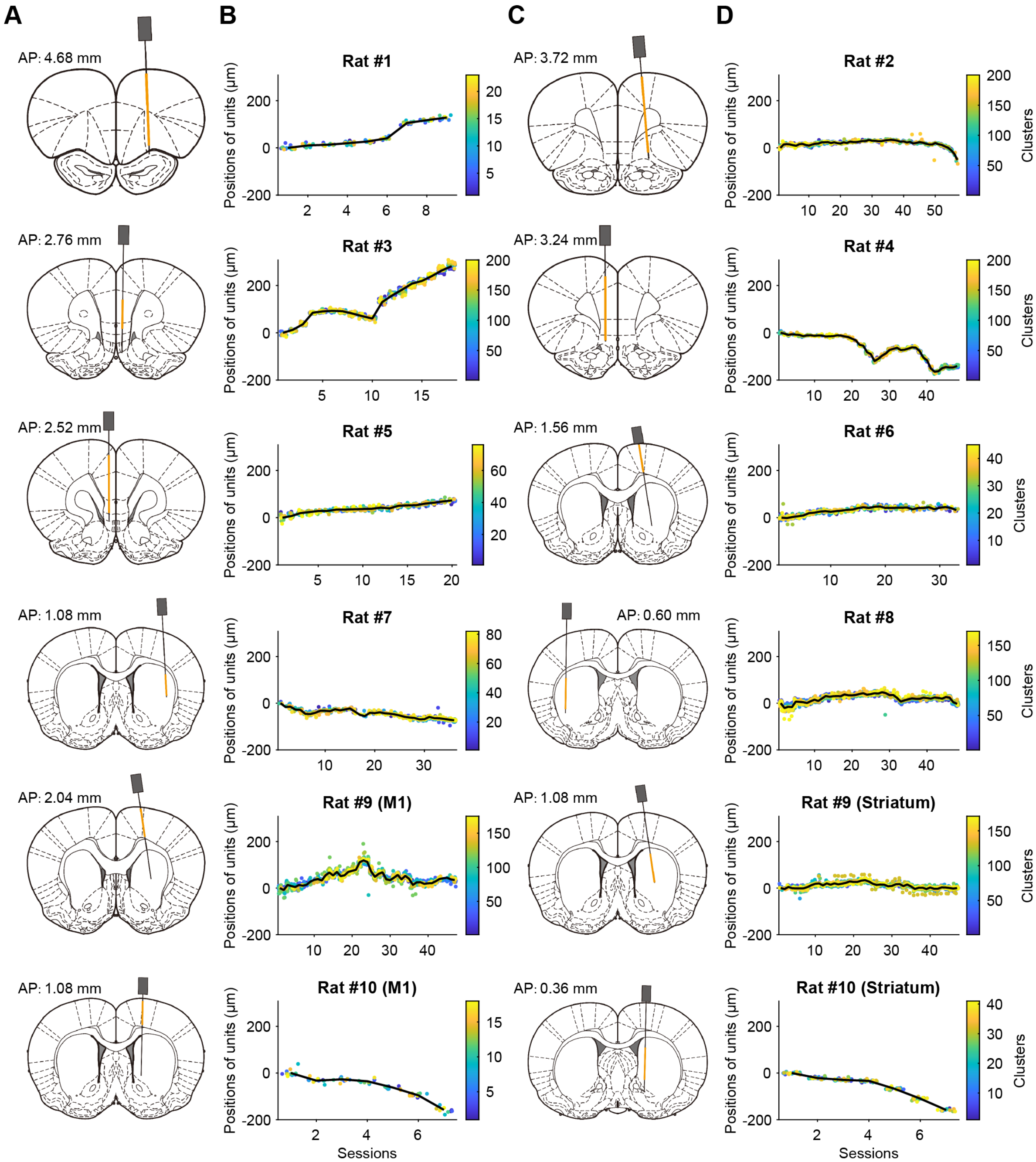

Figure S1. Probe motion across animals. (A, C) Schematics of the recording sites for each dataset. For multi-shank Neuropixels 2.0 probes, only one shank's sites are shown and the other shanks targeted similar regions. Yellow shading denotes the span of recording sites included in the DANT analysis. In two rats (#9 and #10), cortical and striatal recordings were analyzed as separate datasets since they exhibit different relative probe drifts. (B, D) Relative positions of units along the probe across sessions. The black line shows the estimated probe displacement relative to the first session. The positions of units in each detected cluster (up to 200 clusters shown) were normalized to the probe's position in the first session. Colors indicate cluster identity.

59 ## Supplementary Figure 2

60

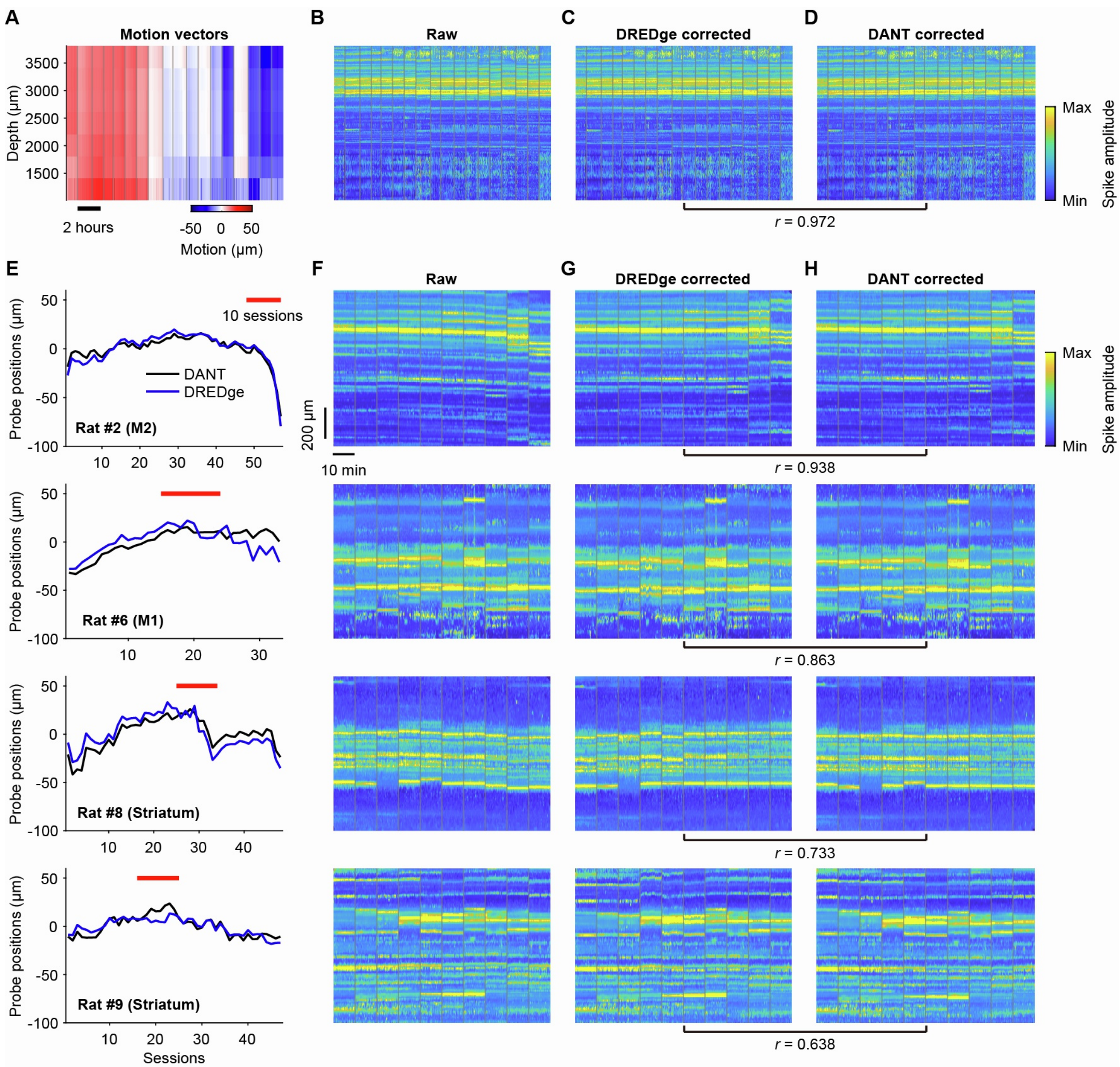

Figure S2. Comparison of DREDge and DANT for probe motion estimation. (A) Heatmap of probe motion estimated by DREDge across depth and time in an example recording spanning 18 sessions (same dataset as in Figure 2C–E), with color indicating displacement magnitude. Gray lines mark session boundaries. Probe position estimates from DREDge and DANT for this dataset are shown in Figure 2D. (B–D) Spike amplitude maps along the probe over time: before motion correction (B), after correction by DREDge (C), and after correction by DANT (D). Colors represent the mean spike amplitude within each spatiotemporal bin (min–max normalized across all sessions); gray lines mark session boundaries. Corrected spike amplitude maps are highly similar between C and D (the Pearson correlation, r, between the two maps is indicated below these panels). (E) Estimated probe positions from DANT (black) and DREDge (blue) in four additional example rats with recordings in cortex or striatum. For DREDge, median values of motion across depths within each session are shown. Red lines indicate the sessions displayed in (F–H). Probe motion is shown relative to the mean position across sessions. (F–H) Same as (B–D) for these four additional rats. Each session consists of a 10-minute recording segment used for motion correction with DREDge.

Supplementary Figure 3

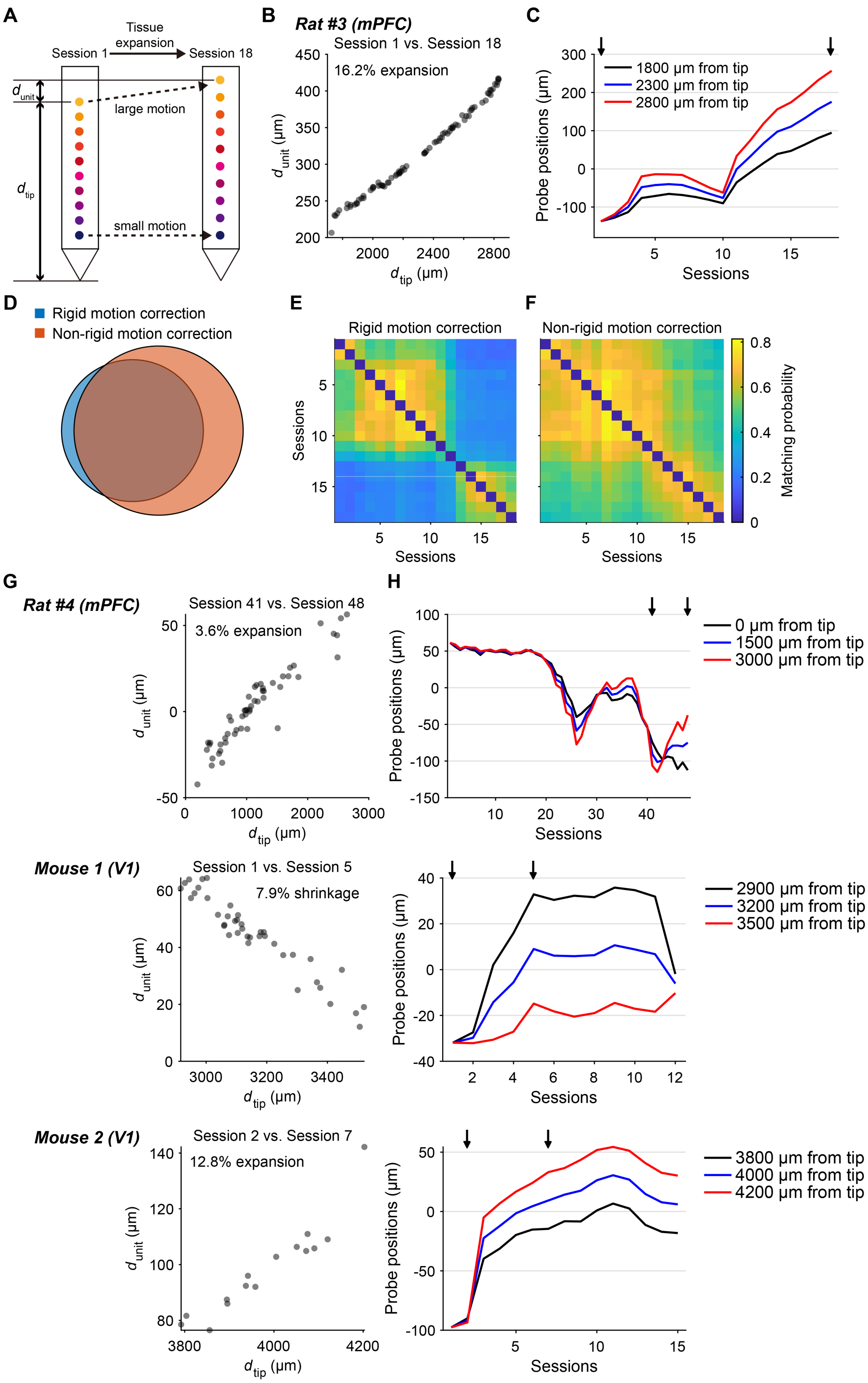

Figure S3. Non-rigid motion correction.

Figure S3 (continued). (A) Schematic illustration of depth-dependent non-rigid motion. (B) Relative motion (y-axis) between session 1 and session 18 for matched unit pairs (each dot is one pair), plotted against the average depth of each pair (x-axis) along a Neuropixels 2.0 probe shank (Rat #3). (C) Estimated depth-dependent probe motion obtained by DANT. (D) Venn diagram showing the overlap of matched units identified by DANT with rigid versus non-rigid motion correction. (E–F) Matching probability matrices showing tracking results of DANT with rigid (E) and non-rigid (F) motion correction. (G, H) Same as (B, C), but for three additional animals. mPFC, medial prefrontal cortex; V1, primary visual cortex.

Supplementary Figure 4

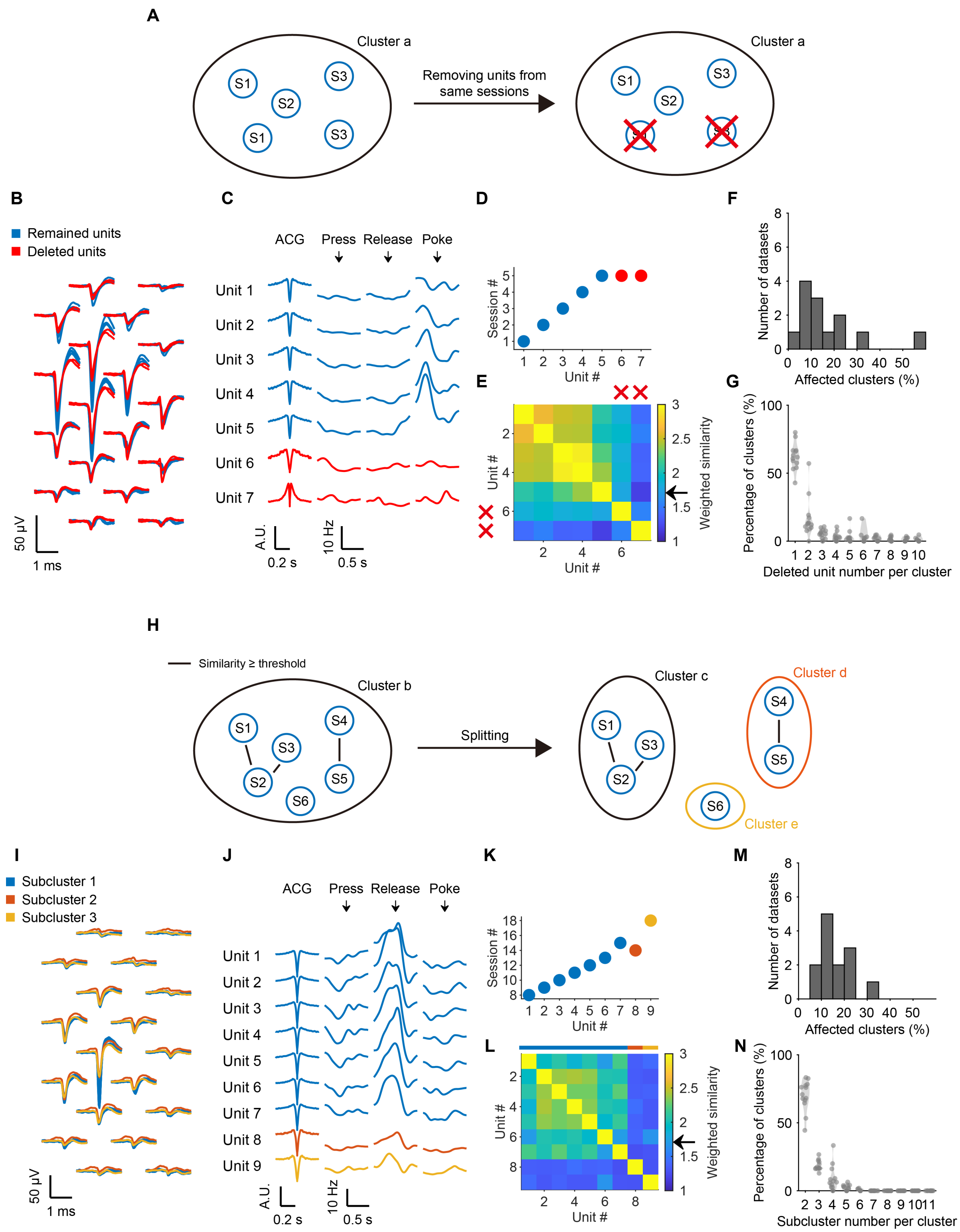

Figure S4. Autocuration for quality control of HDBSCAN clustering results.

Figure S4 (continued). (A) Schematic of Step 1, in which each cluster is constrained to one unit per recording session. When more than one unit from the same session appears, the units with the lowest average similarity to the other units are removed. (B–E) Example cluster containing multiple units from the same session. (B) Overlaid waveforms, with removed units highlighted in red. (C) Smoothed autocorrelograms and PETHs for all units in the cluster. (D) Session origins for each unit. (E) Pairwise similarity matrix; the arrow on the colorbar indicates the threshold derived from LDA. (F) Distribution across datasets of the proportion of clusters affected in Step 1. (G) Distribution across datasets of the number of units deleted per cluster in Step 1. (H) Schematic of Step 2, in which clusters are split into multiple subclusters if subsets of units exhibit higher mutual similarity than to the remaining units, based on the LDA-derived threshold. (I–L) An example cluster that is split into three subclusters in Step 2; panels follow the same layout and notation as in (B–E). (M) Distribution across datasets of the proportion of clusters affected in Step 2. (N) Distribution across datasets of the number of subclusters per original cluster in Step 2.

Supplementary Figure 5

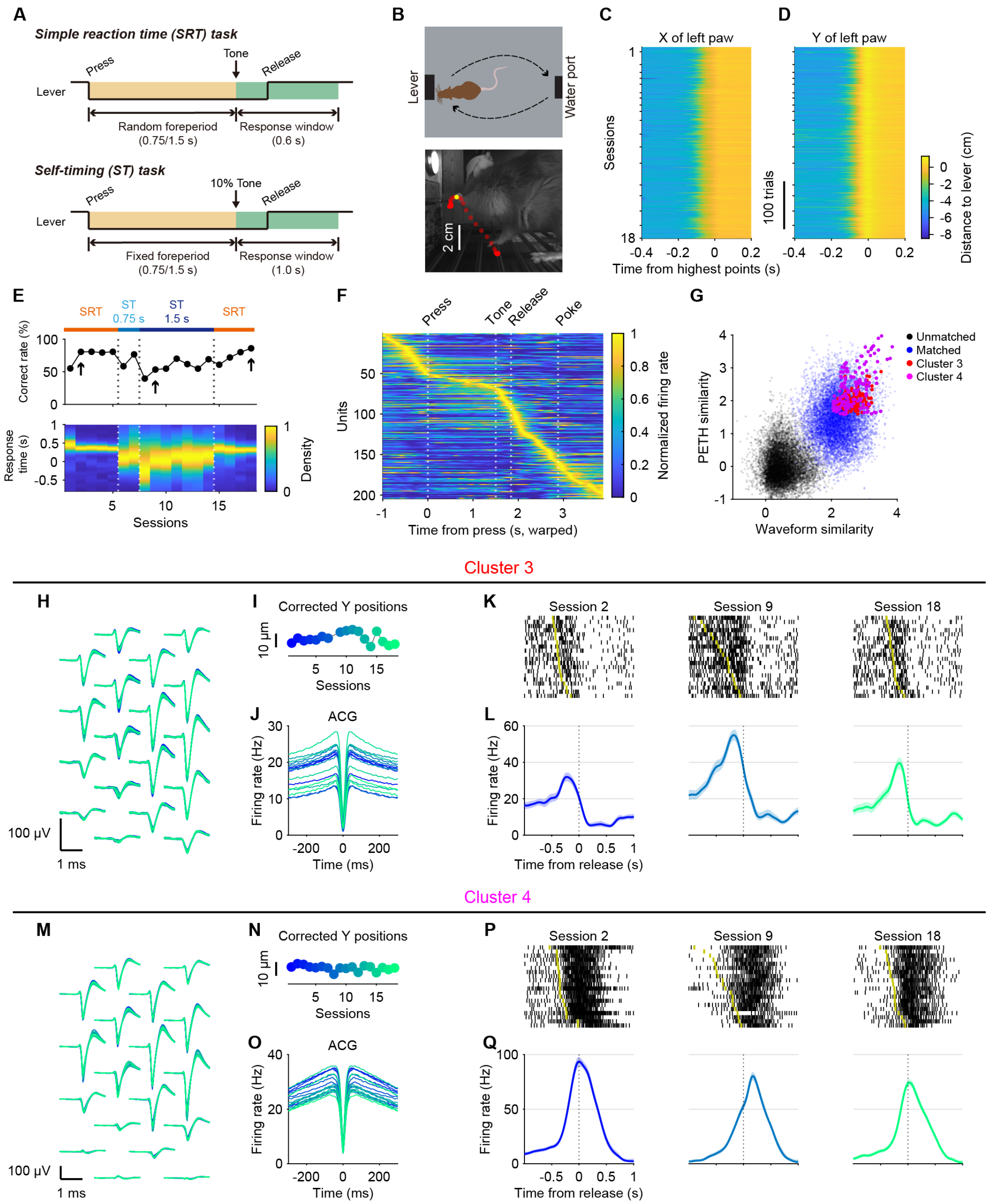

Figure S5. Behavioral paradigm, performance, and example neurons recorded across sessions in an M2 recording. (A) In the simple reaction time (SRT) task, the rat pressed and held a lever for a randomly selected foreperiod (0.75 s or 1.5 s, chosen on each trial) before releasing it within a 0.6-s response window following a brief tone (0.25 s). In the self-timing (ST) task, the rat released the lever after a fixed foreperiod (0.75 s or 1.5 s, constant within a session) within a 1-s response window, with a tone presented in only 10% of trials. (B) Top panel: schematic of the behavioral box in top view. Bottom panel: trajectory of the rat's forepaw during a lever press, with red points indicating left paw positions and a yellow point marking the peak position. The interval between successive points is 10 ms.

Figure S5 (continued). (C, D) Left paw tracking across trials and sessions, with color denoting horizontal (X) or vertical (Y) position relative to the lever. (E) Training protocol and behavioral performance across 18 sessions. Top panel shows the sequence of training phases, with the number beneath "ST" indicating the fixed foreperiod used in the ST task. Middle panel plots the proportion of correct trials across sessions. Bottom panel displays the distribution of response times, defined as the duration from the end of the foreperiod to lever release (for premature releases, this value is negative). (F) Time-warped population PETHs for 1.5-s foreperiod trials in a single session (session 2 in the SRT task). PETHs were aligned and linearly warped between lever press, lever release, and nose poke, and were min-max normalized to 0 and 1. Units were ordered by the positions of their peak modulation. (G) PETH similarity versus waveform similarity for all pairs of units across 18 sessions, with red and purple dots highlighting unit pairs from the example neurons (153 pairwise comparisons per neuron). (H–J) Same layout as in Figure 4F–H for a different neuron (Cluster 3). Spiking activity is aligned to the time of lever release. (K) Spike raster for 1.5-s foreperiod trials aligned to the lever release. Yellow ticks indicate the end of the foreperiod. (L) PETHs aligned to the time of lever release. Shaded areas indicate the 95% confidence intervals. (M–Q) Same layout as (H–L) for a different neuron (Cluster 4).

67 ## Supplementary Figure 6

68

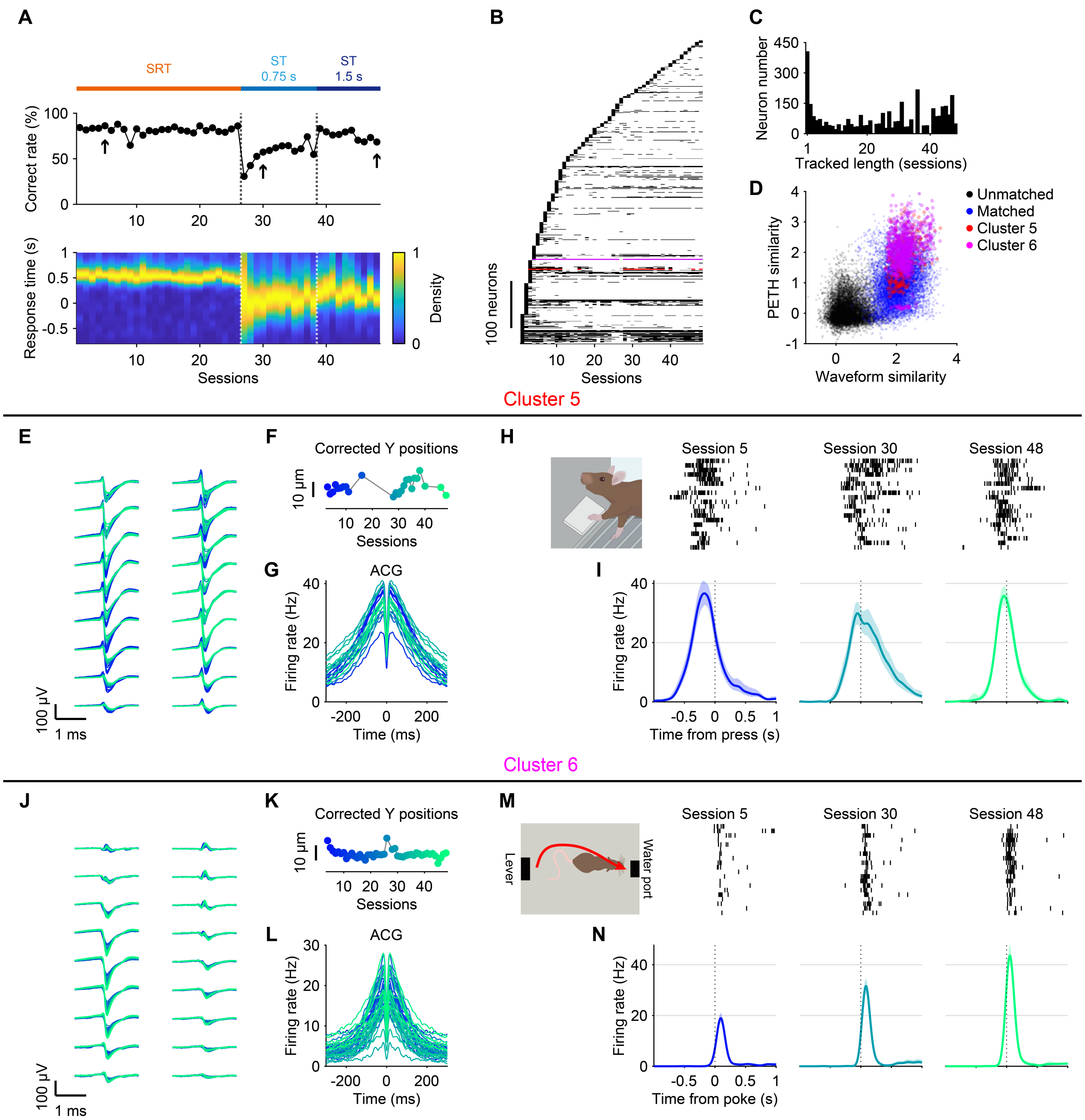

Figure S6. Example neurons recorded across sessions in a DLS recording. (A) Same layout as in Figure S5E for a different animal. (B) Presence of unique neurons across sessions, ordered by first appearance. Red and purple lines indicate the example neurons. (C) Distribution of tracked length (number of sessions in which each neuron was recorded) for all neurons. (D) PETH similarity versus waveform similarity for all pairs of units across 48 sessions, with red and purple dots highlighting unit pairs from the example neurons. (E–I) Same layout as Figure S5H–L for a different neuron (Cluster 5), with spike rasters and PETHs aligned to the time of lever press. (J–N) Same layout as (E–I) for a different neuron (Cluster 6), with spike rasters and PETHs aligned to the time of nose poke.

Supplementary Figure 7

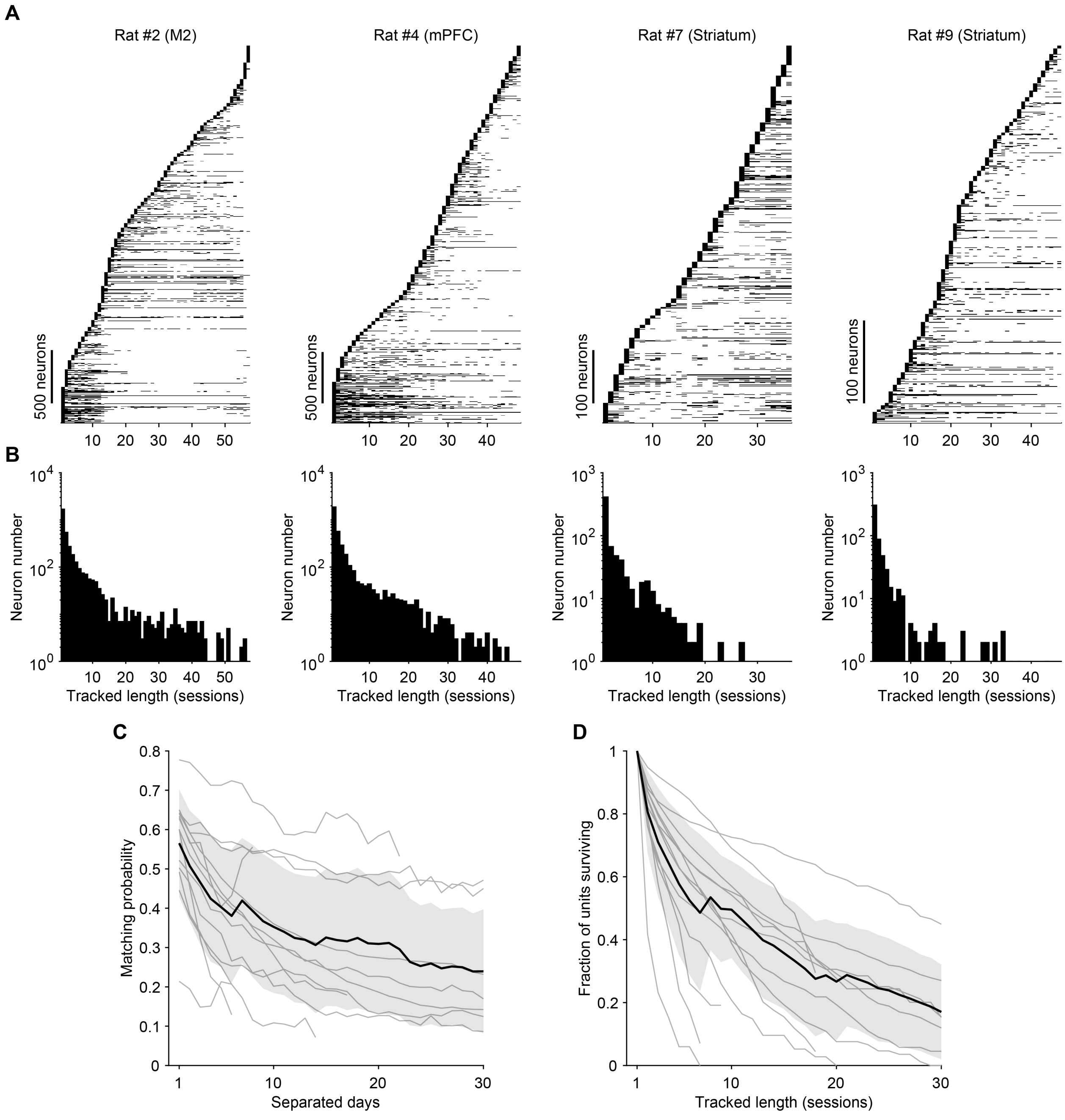

Figure S7. Tracking performance across animals. (A) Presence of unique neurons across sessions detected by DANT in four additional animals. (B) Distribution of tracked length for detected neurons. (C) Matching probability of identifying the same unit between two recordings separated by *n* days across all datasets. Black lines represent mean values, and shaded areas represent standard deviations. (D) Proportion of units that could be tracked for at least *n* sessions across all datasets. Black lines represent mean values, and shaded areas represent standard deviations.

Supplementary Figure 8

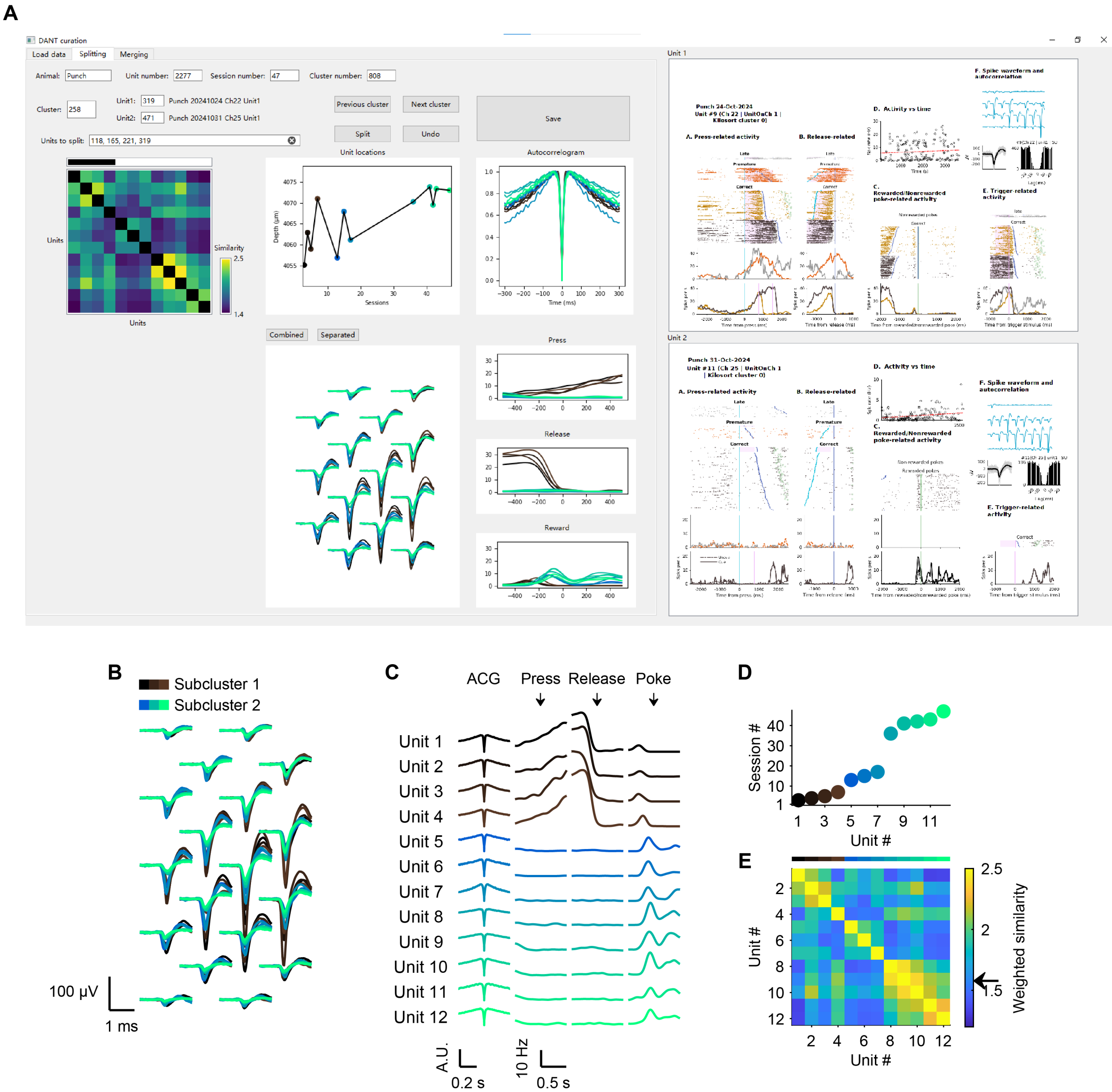

Figure S8. Manual curation of DANT results: an example cluster split into two subclusters with different functional properties. (A) Graphical user interface (GUI) used for manual curation of DANT results. The GUI allows users to visualize and curate the clusters identified by DANT. Displayed features include a similarity matrix between units, unit locations across sessions, motion-corrected waveforms, autocorrelograms, PETHs for three task events, and custom figures illustrating each unit's functional tuning. The example cluster from Rat #9 was manually divided into two subclusters. Subcluster 1 (the first four units displayed in black) exhibited ramping activity after lever press and before lever release, in clear contrast to subcluster 2 (units displayed in a blue-to-green colormap), which was primarily activated prior to nose poke for reward. The two custom figures on the right correspond to the last unit of subcluster 1 and the first unit of subcluster 2. (B–E) The example cluster from panel (A) is shown using the same layout as in Figure S4B–E.

Supplementary Figure 9

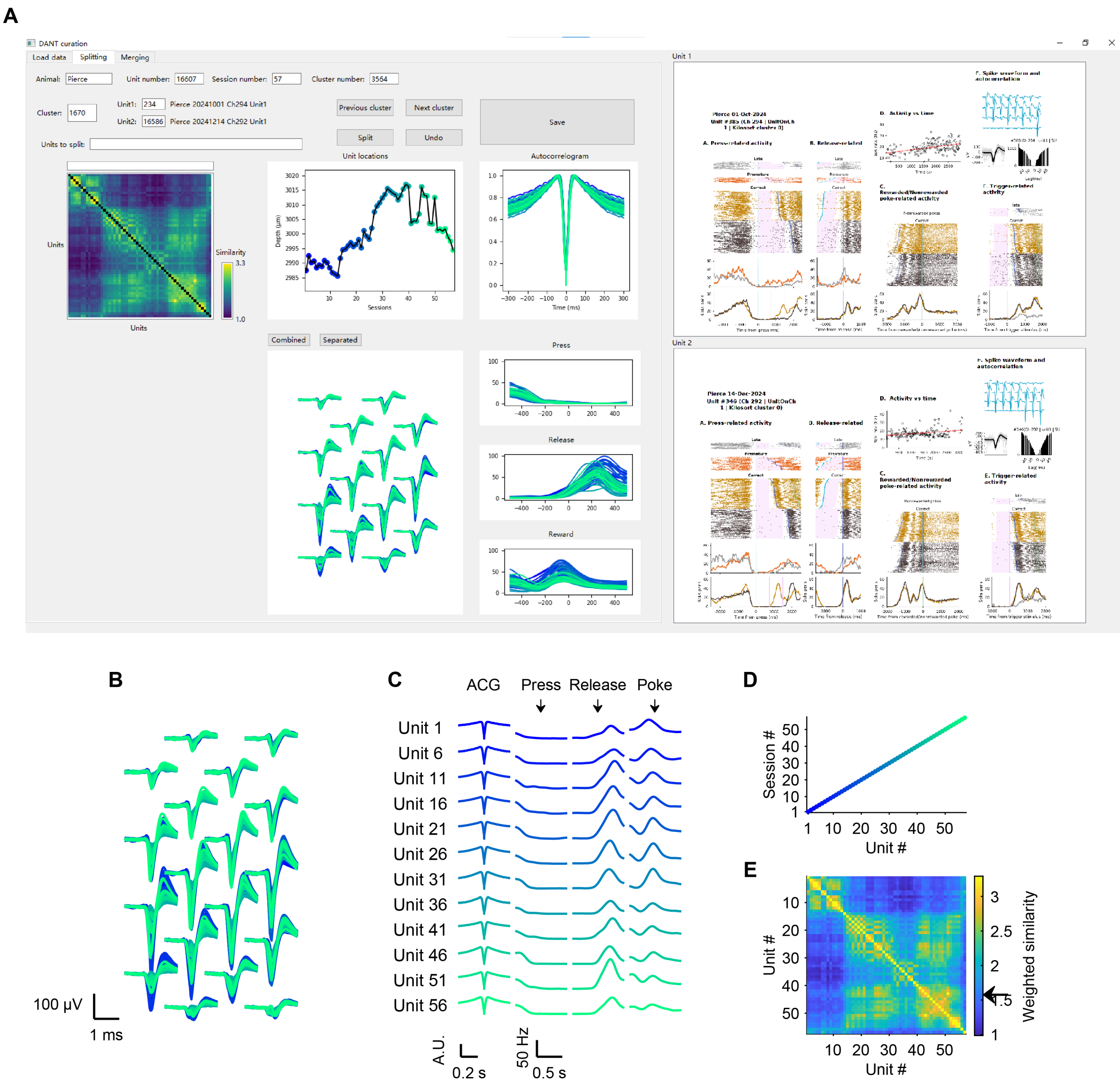

Figure S9. Manual curation of DANT results: an example cluster with consistent functional properties. Same layout as in Figure S8, but showing a cluster from Rat #2 across 57 sessions in which all units passed manual inspection, and no units were excluded.

Supplementary Figure 10

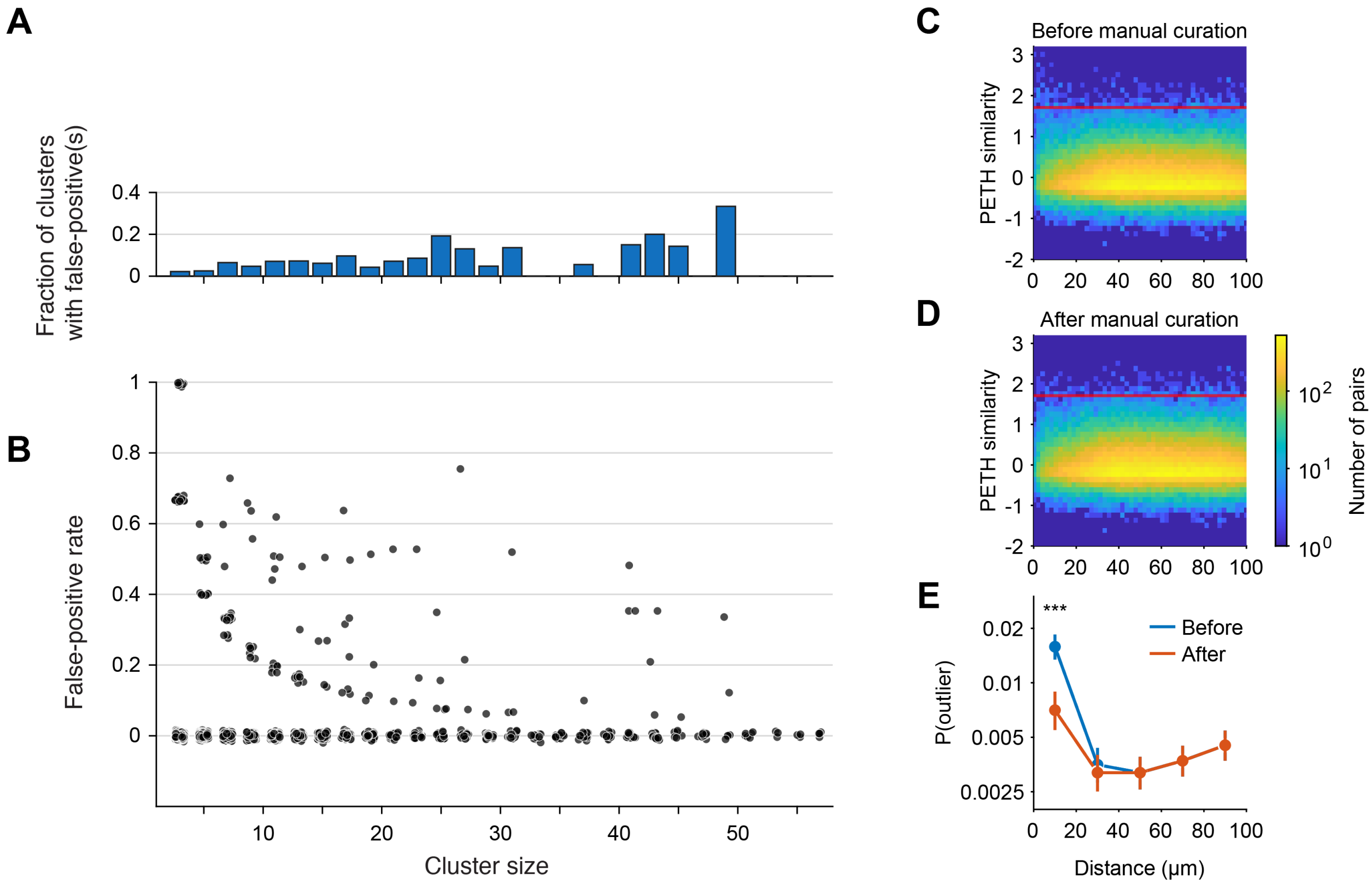

Figure S10. False positives and false negatives identified by manual inspection. (A) Fraction (in each bin) of clusters containing at least one false positive, plotted against cluster size. (B) False-positive rate for each inspected cluster, plotted against cluster size across all datasets. (C) Heatmap of PETH similarity for unmatched unit pairs identified by DANT across consecutive days, plotted as a function of inter-unit distance. Four datasets from motor cortex and dorsolateral striatum recordings were included. Pixel intensity reflects pair counts. The red line represents the threshold used to identify outliers. (D) Same as (C), but after manual curation, including merging clusters that were presumed to be the same neurons. (E) Proportion of outliers among unmatched pairs before and after manual curation, grouped into 20-μm distance bins. Error bars indicate 95% confidence intervals. ***: $p < 0.001$.

Supplementary Figure 11

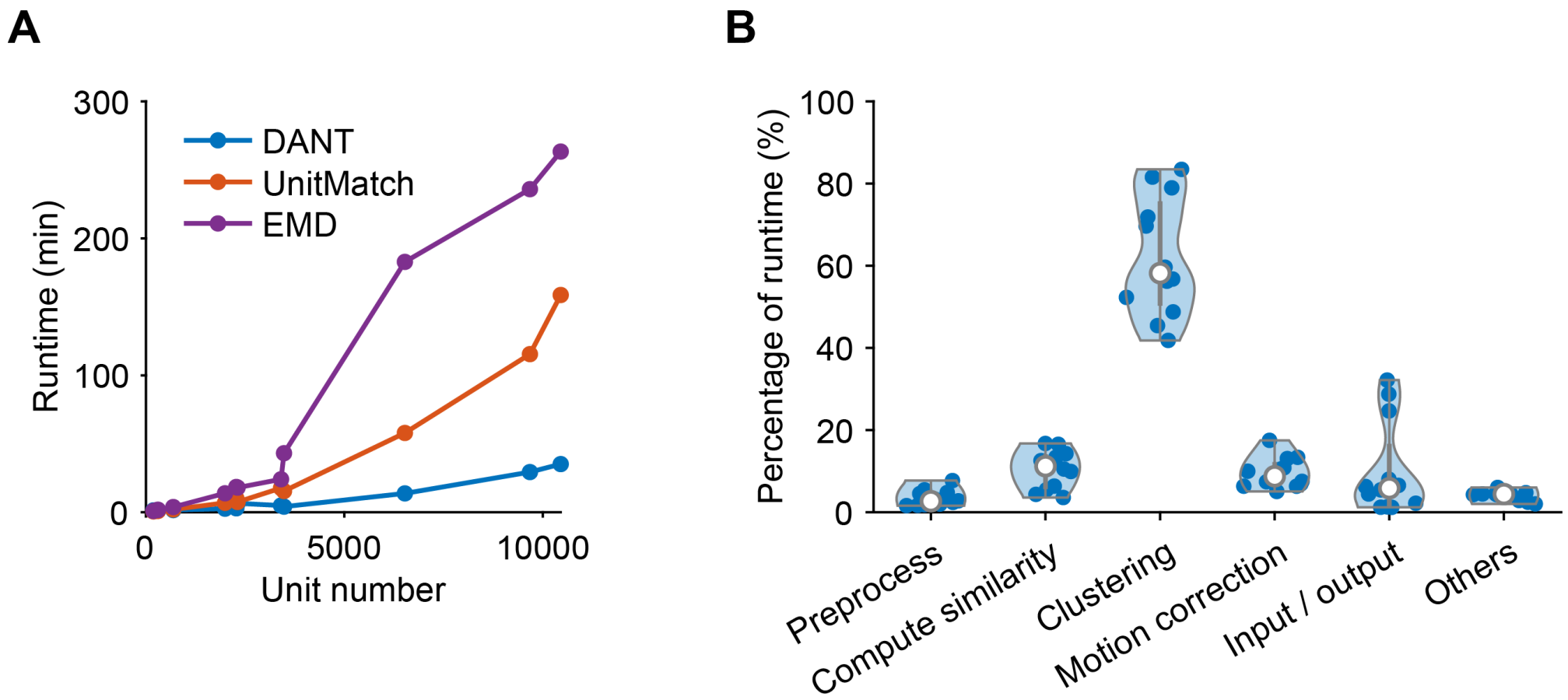

Figure S11. Evaluating runtime performance. (A) Total runtime across 11 datasets for different methods. (B) Breakdown of runtime proportions for individual processes within DANT.
